# Photochemical Fingerprinting Is a Sensitive Probe for the Detection of Synthetic Cannabinoid Receptor Agonists; toward Robust Point-of-Care Detection

**DOI:** 10.1021/acs.analchem.2c02529

**Published:** 2023-01-04

**Authors:** Rachael C. Andrews, Benedict May, Federico J. Hernández, Gyles E. Cozier, Piers A. Townsend, Oliver B. Sutcliffe, Tom S. F. Haines, Tom P. Freeman, Jennifer Scott, Stephen M. Husbands, Ian S. Blagbrough, Richard W. Bowman, Simon E. Lewis, Matthew N. Grayson, Rachel Crespo-Otero, David R. Carbery, Christopher R. Pudney

**Affiliations:** † Department of Chemistry, University of Bath, BathBA2 7AY, U.K.; ‡ Centre for Sustainable Chemical Technology, University of Bath, BathBA2 7AY, U.K.; § Department of Biology and Biochemistry, University of Bath, BathBA2 7AY, U.K.; ∥ Department of Chemistry, Queen Mary University of London, LondonE1 4NS, U.K.; ⊥ School of Applied Sciences, University of the West of England, BristolBS16 1QY, U.K.; # MANchester DRug Analysis & Knowledge Exchange (MANDRAKE), Department of Natural Sciences, Manchester Metropolitan University, ManchesterM15 5GD, U.K.; ¶ Department of Computer Science, University of Glasgow, GlasgowG12 8QQ, U.K.; ∇ Department of Psychology. University of Glasgow, GlasgowG12 8QQ, U.K.; ○ Department of Pharmacy and Pharmacology, University of Glasgow, GlasgowG12 8QQ, U.K.; ⧫ School of Physics and Astronomy, University of Glasgow, GlasgowG12 8QQ, U.K.; †† Centre for Therapeutic Innovation, University of Bath, BathBA2 7AY, U.K.

## Abstract

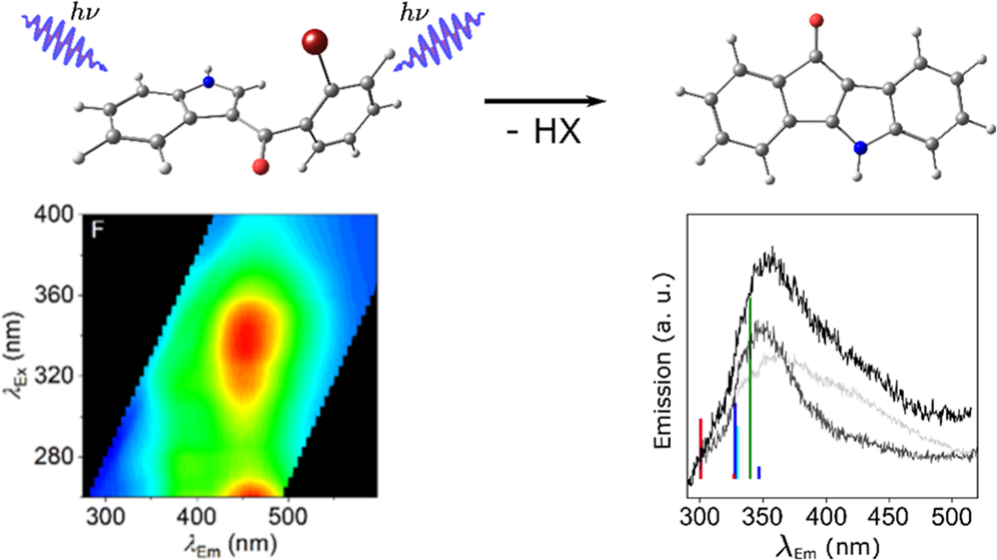

With synthetic cannabinoid receptor agonist (SCRA) use still prevalent across Europe and structurally advanced generations emerging, it is imperative that drug detection methods advance in parallel. SCRAs are a chemically diverse and evolving group, which makes rapid detection challenging. We have previously shown that fluorescence spectral fingerprinting (FSF) has the potential to provide rapid assessment of SCRA presence directly from street material with minimal processing and in saliva. Enhancing the sensitivity and discriminatory ability of this approach has high potential to accelerate the delivery of a point-of-care technology that can be used confidently by a range of stakeholders, from medical to prison staff. We demonstrate that a range of structurally distinct SCRAs are photochemically active and give rise to distinct FSFs after irradiation. To explore this in detail, we have synthesized a model series of compounds which mimic specific structural features of AM-694. Our data show that FSFs are sensitive to chemically conservative changes, with evidence that this relates to shifts in the electronic structure and cross-conjugation. Crucially, we find that the photochemical degradation rate is sensitive to individual structures and gives rise to a specific major product, the mechanism and identification of which we elucidate through density-functional theory (DFT) and time-dependent DFT. We test the potential of our hybrid “photochemical fingerprinting” approach to discriminate SCRAs by demonstrating SCRA detection from a simulated smoking apparatus in saliva. Our study shows the potential of tracking photochemical reactivity via FSFs for enhanced discrimination of SCRAs, with successful integration into a portable device.

Synthetic cannabinoid receptor agonists (SCRAs), colloquially known as ‘spice,’ are a class of designer recreational drugs, commonly taken to mimic the effects of tetrahydrocannabinol (THC). At the outset, these drugs were synthesized as cannabimimetics with potential pain-relieving symptoms; however, the additional psychoactive side effects have rendered these compounds unsuitable for pharmaceutical use.^1^ At the end of 2021, there were 224 SCRA compounds formally notified by the European Monitoring Centre for Drugs and Drug Detection (EMCDDA), with the first new SC compound identified in seized drug samples in 2008.^2^ In order to circumvent legislation that bans novel psychoactive substances (NPS), more structurally diverse compounds are released into circulation every year.^3^ With newer SCRA varieties exhibiting higher affinity for CB_1_ and CB_2_ receptors, these drugs pose an increasing threat to users with fatal side effects, including, but not limited to, coronary artery thrombosis, ischemic stroke, and psychosis.^4^

Importantly, detection of these compounds is challenging, with routine NPS testing methods unable to identify the presence of many SCRAs.^3^ Point-of-care drug testing is an important modality to support users, and it is vital that the detection method is both fast and portable. There are a variety of currently implemented methods, including screening, colorimetric detection, immunochemical assays, and chromatographic methods. Although there are advantages and disadvantages to each method, colorimetric detection is favored in a point-of-care setting due to it being both rapid and portable. However, these tests tend to be specific to individual structures and fail to detect newer SCRAs such as Cumyl-PEGACLONE.^5^ In contrast, chromatographic methods, including LC–MS/MS, are more advanced detection methods that can resolve a large range of compounds with a low limit of detection.^6^ However, the high associated costs and lack of portability render this method unsuitable for mobile drug testing.

We have recently demonstrated that fluorescence spectral fingerprinting (FSF) has potential as a rapid point-of-care test for SCRAs.^7^ For example, to act as a probe of SCRA use. We have shown that common SCRA compounds, in both pure samples and oral fluid, produced individual FSFs, with the possibility to extract information about the structure and concentration of these substances.^7^ We have previously suggested that part of the sensitivity of SCRA FSFs to different, structurally similar molecules may arise from differences in cross conjugation and associated effects on electronic transitions related to fluorescence.^7^

We hypothesize that the rapid detection of SCRAs could be enhanced by augmenting the FSF detection methodology through photochemical reactivity tracking. SCRAs are typically built on a scaffold that includes a central ‘core’ group. There are over 10 different moieties that have been identified as core groups in SCRA compounds, including pyrrole, carbazole and, more recently, oxoindole, found in the emerging “OXIZID” SCRA group.^8−10^ However, indole and indazole are by far the most commonly identified core in SCRA compounds, found in over 75% of SCRAs notified by the EMCDDA.^8^ They are both photochemically active and sensitive to substituents on the ring system.^11^

Herein, we use a model SCRA homologue series to explore the molecular determinants of SCRA FSF sensitivity and the potential for tracking photochemical reactivity of SCRAs via changes in FSF for enhanced detection. Density-functional theory (DFT) and time-dependent DFT (TDDFT) calculations suggest a molecular rationale for the detection sensitivity. Finally, we demonstrate the feasibility of using the combined photochemical/FSF “photochemical fingerprinting” approach to detect street material in saliva.

## Results and Discussion

### Exploring the Sensitivity of SCRA FSFs

**Figure 1:**
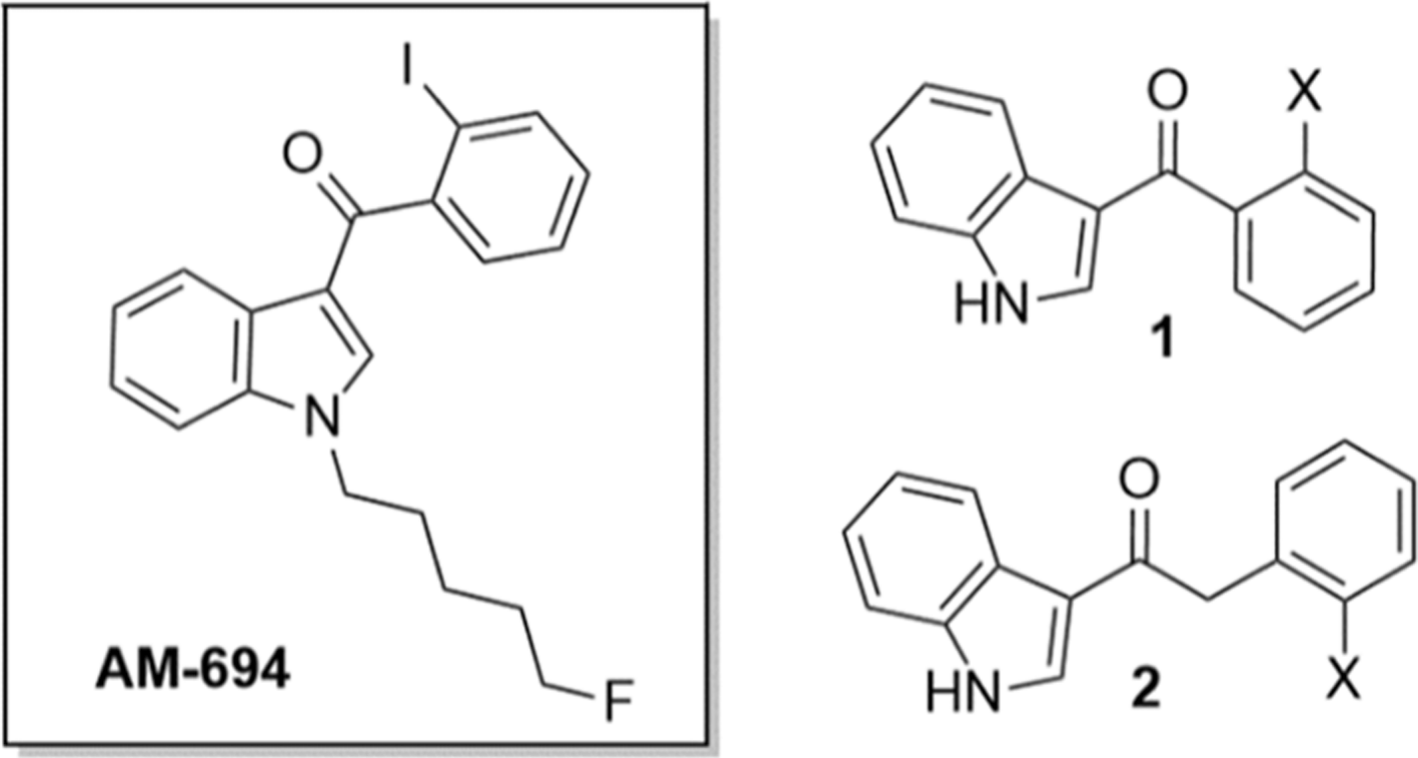
Model series of 8 compounds, based on the core, linker, and ring section of AM-694. Compounds **1a–d** contain a methanone linker and **2a–d** contain an ethanone linker group, all with varied halogen substitution at the 2-position.

**Scheme 1:**
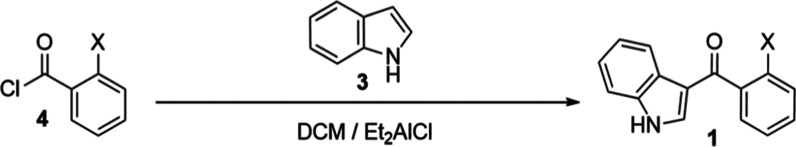
Synthesis of Compounds **1a–d**; Atom X Denotes Halogen Substitution (X = F, Cl, Br, and I).

**Scheme 2:**
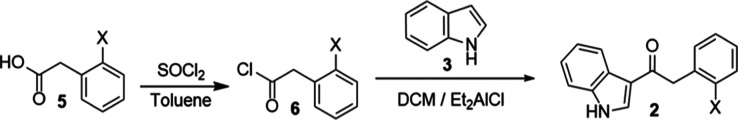
Synthesis of Compounds **2a–d**; Atom X Denotes Halogen Substitution (X = F, Cl, Br, and I)

Our previous work suggests that SCRA FSFs are highly sensitive to chemical substitution, which we posited may be due to changes in electronic structure and the degree of cross-conjugation. Therefore, we have designed a model chemical series to explore these perturbations. The model series are shown in Figure 1. These compounds are based on the SCRA, AM-694, although they exclude the fluoropentyl “tail” on the indole nitrogen for synthetic simplicity. Moreover, “tail-less” SCRAs have recently been reported (e.g., MDMB-5Br-INACA) and so the model system is both experimentally tractable and also relevant as an example SCRA.^12^ We note that all eight of the model series were successfully synthesized via a selective acylation of indole (**3**) at the 3-position in the presence of Lewis acid, diethylaluminum chloride (Figure 1, Schemes 1 and 2). All eight compounds vary by their halogen substitution at the 2-position on the benzene ring and whether the indole is attached to the halogenated phenyl with a methanone or ethanone linker group. All eight compounds were characterized using ^1^H, ^13^C, and ^19^F (where applicable) NMR, mass spectrometry, IR spectroscopy, and melting point (data in Supporting Information).

**Figure 2:**
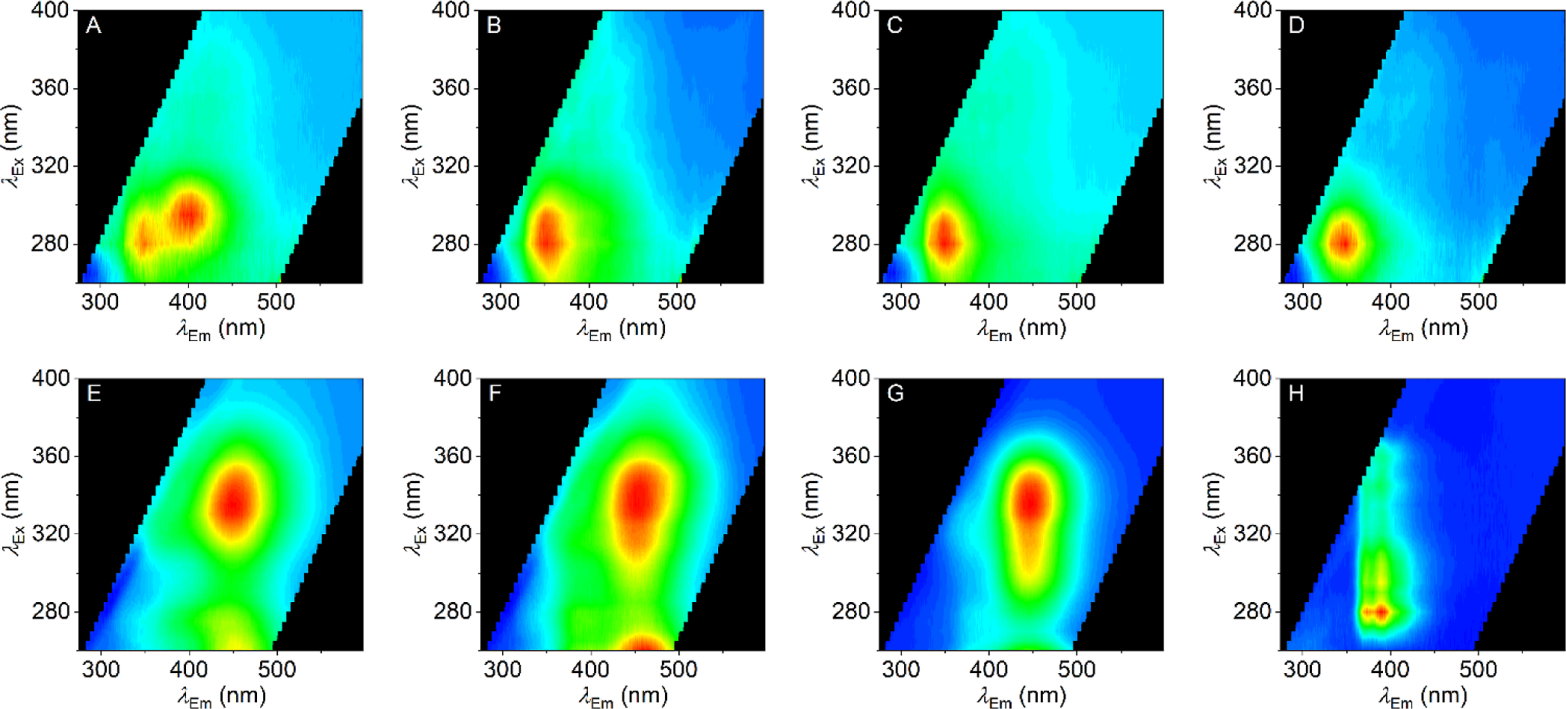
(A–D) FSFs for methanone-linked series of SCRA analogues **1a–d** (A = **1a**, B = **1b**, C = **1c**, and D = **1d**), collected prior to sample irradiation. (E–H) FSFs for ethanone-linked series of SCRA analogues **2a–d** (E = **2a**, F = **2b**, G = **2c**, and H = **2d**), collected prior to sample irradiation. Red coloration represents emission with a relative intensity of 1, and blue represents an intensity of 0. Conditions: 0.01 mg/mL in methanol, 20 °C, and 1 cm path length.

Figure 2 shows the resulting FSFs for all eight compounds, **1a–d** and **2a–d**. These data show that each FSF varies between linker groups and halogen substitution, and that these shifts are complex. Below, we describe the broad trends.

We have previously found that fitting SCRA FSFs with a modified Fraser-Suzuki function allows accurate quantification of relatively complex spectral fingerprints.^7^Equation 1
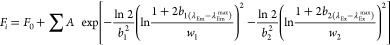
1 is a sum of two-dimensionally skewed Gaussian functions, where *A* is the amplitude, *w* is the full width at half maximum (fwhm), and *b* is a skewness parameter. In practice, we find that the FSFs for **1a–d** and **2a–d** are accurately captured with either one or two components, and the resulting fit parameters are given in Table S1.

Compounds **1a–d** (Figure 2A–D) each show a major spectral feature at λ_Ex_^max^ ≈ 280 nm and λ_Em_^max^ ≈ 350 nm, whereas **2a–d** (Figure 2E–H) each have a major component at λ_Ex_^max^ ≈ 325 nm and λ_Em_^max^ ≈ 450 nm, with the notable exception of **2d**. That is, the addition of a methylene-bridge carbon at the linker region is sufficient to shift the λ_Ex_^max^ by ∼80 nm but without a similar dramatic change in Stokes shift, ∼85 nm. Indeed, the addition of this extra carbon to the linker region drives the FSFs to be effectively unrecognizable as typical indole fluorescence. We have previously observed similar “complex” FSFs for SCRAs, and have suggested that these large shifts might arise from changes in cross-conjugation between the two ring systems.^7^

Moreover, there are clear differences with varying the halogen substitution, both with methanone- and ethenone-linked compounds. Broadly, increasing the electronegativity of the halogen substitution from iodine to fluorine causes a blue shift in the λ_Em_^max^ for all compounds **1a–d** and **2a–d**; though this is accompanied by a nonobvious trend in λ_Ex_^max^ (Table S1). Most notably, the observation of measurable shifts in the FSFs on halogen substitution suggests the fluorescence of the indole is sensitive to electronic/structural shifts at sites remote from the core ring system but still part of the conjugated system.

These data show that FSFs are extraordinarily sensitive to subtle changes in chemical structure and point to the rationale for their sensitivity toward different SCRAs that we have observed previously.^7^ More specifically, we envisage shifts in the distribution of conformational states and consequently electronic structures. Therefore, shifts in cross-conjugation may be the drivers of the observed differences in the FSFs. We consider this in detail below.

### UV Irradiation Produces a Molecule-Specific FSF

Observations of FSF measurements taken over extended time periods suggested that degradation of the study compounds was occurring under UV irradiation. Indeed, indoles and indazoles are known to be photochemically reactive on UV irradiation.^13^ Therefore, we have explored the potential for tracking photochemical reactivity as an additional discriminatory probe of SCRAs and SCRA homologues.

Figure S1 shows FSFs for five SCRA compounds, MDMB-4en-PICA, MDMB-4en-PINACA, MDMB-FUBICA, MDMB-FUBINACA and BZO-HEXOXIZID (also known as MDA-19) before and after 2 h of irradiation (irradiation at 300 nm, corresponding to a peak in the absorption spectra as discussed below; continuous wave irradiation ∼0.2 mW). Corresponding pre-/postirradiation difference maps are shown in Figure S1. These SCRA compounds contain a range of structural groups including indole, indazole and oxoindole cores, and amino acid-derived linked groups.

All five compounds are affected by UV irradiation, with the evolution of new products evident in all five difference maps. Although the majority of predegradation FSFs appear remarkably similar (with exceptions such as BZO-HEXOXIZID), the postdegradation FSFs are highly distinct, indicating potential for discrimination following degradation. For example, despite the similarity of the predegradation FSF, we find completely different postdegradation products for different “tails” for example, MDMB-4en-PINACA and MDMB-FUBINACA, Figure S1B,D, respectively. In addition, some SCRAs, such as BZO-HEXOXIZID, show minimal features in their FSFs but can be easily observed postirradiation.

**Figure 3:**
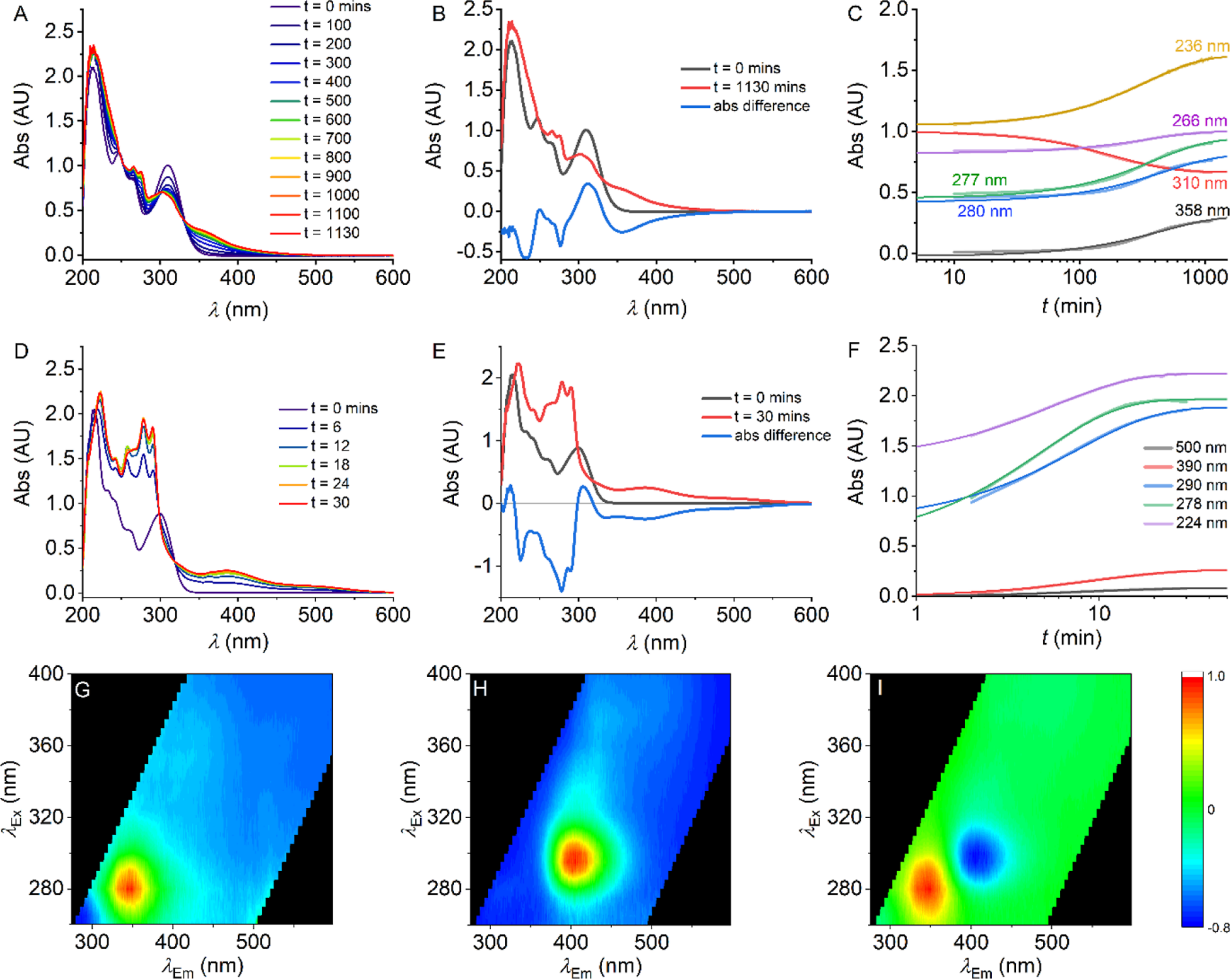
(A–C) Time series of absorbance scans of **1d** as it was degraded (A), a plot comparing the initial and final absorbance spectra (B), the changes in absorbance at wavelengths of interest (C). (D–F) Time series of absorbance scans of **2d** as it was degraded (D), a plot comparing the initial and final absorbance spectra (E), the changes in absorbance at wavelengths of interest (F). (G–I) FSFs for **1d** collected prior to sample irradiation (G) and post degradation (H); red coloration represents emission with a relative intensity of 1, and blue represents emission with a relative intensity of 0. A heat map (I) showing the differences in emission intensity.

We wished to use our model series of SCRA analogues to study the observed degradation in detail. Figure 3 shows the changes in the absorption spectra with respect to time upon irradiation for **1d** (Figure 3A–C) and **2d** (Figure 3D–F). We observe similar spectral changes for compounds **1a–c** and **2a–c**, respectively. For compounds **1a–d**, prior to irradiation, the spectra have defined absorption peaks at λ ∼310, ∼265, and ∼245 nm. Figure 3B shows the difference absorption spectrum pre- and postirradiation. From this, we find that the spectral changes for **1d** can be defined by absorbance changes at 6 defined wavelengths. Figure 3C shows the time dependence of the spectral changes for **1d** at these wavelengths. For each of these spectral features, we find that the rate can be adequately defined by a single-exponential function
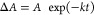
2where *A* is the amplitude, *k* is the rate constant obtained from the time-dependent absorption change trace, and Δ*A* is the total absorbance change. That these data can be adequately fit with a single exponential function is suggestive of a single (photochemical) process. We note that the negative amplitude associated with the 310 nm peak convolves nearby peaks with an associated kinetic component, but the extracted rate constants are effectively the same. Moreover, these spectral changes all proceed with a similar rate constant [average *k* = 0.18 ± 0.07 (SD) s^–1^] for **1d**, suggesting that the absorption changes are reflecting the same rate-limiting process.

The data for **2d** are similar to those for **1d**, in that irradiation causes a gain of a similar new spectral feature (Figure 3D,E), but red shifted by ∼10 nm compared to **1d**. However, from Figure 3F, the rate constant for the observed shifts on irradiation are ∼50 times faster for **2d** versus **1d**, (*k* = 8.6 ± 2.9 (SD) s^–1^ for **2d**) and this trend is similar for the remaining members of the halogen series (**1** and **2**), as discussed below. These data show that the presence of an ethanone linker gives a dramatically increased rate of photochemical activity to produce a similar product. Moreover, we find that for compound series **1** and **2**, the kinetics are similar for the fluoro- and chloro-analogues, and an order of magnitude larger (and similar) for the bromo- and iodo-analogues (Figure S2). That is, we observe a trend of increasing photochemical sensitivity with increasing electronegativity of the halogen group.

Although we cannot definitively identify kinetically distinct species (we anticipate a range of photochemical products as we discuss below), we are able to at least select for a similar ‘end point’ of a photochemical step(s) with respect to time as a distinct exponential phase. For example, in the case of **1d**, at ∼1000 min and for **2d** at ∼30 min, given the irradiance of the light source used. Figure 3G,H shows FSFs collected before and after the irradiation of **1d**, respectively. Figure 3I maps the differences in the relative intensities of the features present in the FSF of **1d**. Equivalent plots are shown in Figure S3 for compounds **2a–d**. Fitting these data to eq 1 shows a single major species present in both the pre- and postdegradation spectra of **1d**. These species are distinct (λ_ex_ ∼ 281 nm, λ_em_ ∼ 346 nm and λ_ex_ ∼ 296 nm, and λ_em_ ∼ 404 nm). That is, irradiation of **1d** to the kinetic endpoint causes an effective complete loss of the parent fluorophore with formation of a single distinct fluorescent species. Moreover, the observed changes suggest mechanistic information on the photochemical breakdown. That is, the emergence of a new distinct fluorophore (Figure 3H) suggests that the photochemical reaction mechanism involves the production of a new fluorescent species or a shift in the equilibrium of a specific electronic structure. Indeed, each of **1a–d** give a distinct shift in the FSF on irradiation (Figure S3). These data are evidence that even highly structurally similar SCRA analogues can be discriminated based on photochemical reactivity, which can be tractably monitored via shifts in their FSF.

### Computational Modeling

Our data suggests that subtle shifts in the degree of cross conjugation (through varying linker length), and electronegativity at sites separate from the fluorophore, is sufficient to measurably alter the molecular FSFs and photochemical reactivity. To investigate the origin of this sensitivity and the photochemical reactivity, a range of in silico methods were used. Calculations were performed on compounds **1a–d** and **2a–d** at the B3LYP-D3(BJ)/def2SVP level of theory. All calculations were performed under the integral equation formalism polarizable continuum model (IEF-PCM) solvation model for methanol. Due to the flexible nature of the linker groups, conformational searches were performed for each molecule using the OPLS3e force field in Schrodinger’s Macromodel (Ver. 12.6),^14^ and the resultant conformers were taken forward to DFT. Optimizations were performed in Gaussian 16 (Rev. A.03),^15^ and the quasiharmonic free energies were obtained at a constant temperature of 298.15 K and a concentration of 1 mol dm^–3^.^16^Table S2 shows a range of data obtained for **1a–d** and **2a–d**, including the Boltzmann weighting of each conformer. Since cross-conjugation was suspected to influence the FSFs produced, the planarity of two ring systems either side of the linker group in compounds **1a–d** was investigated by measuring the dihedral angle over carbon-3, -10, -11, and -16 (Table S2).

**Scheme 3:**
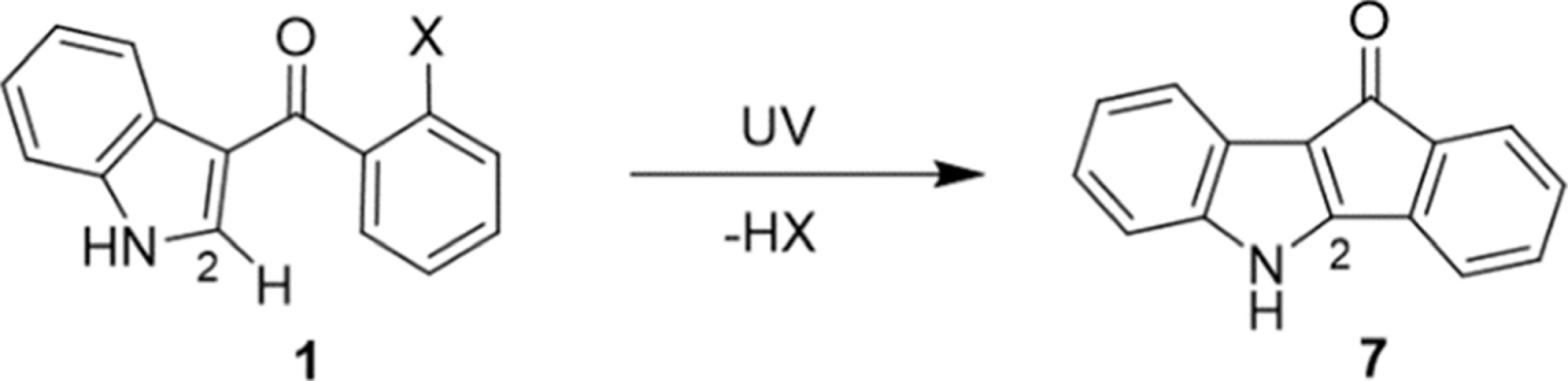
Hypothesized Degradation Mechanism for Halogenated Compounds **1a–d** Based on Previous Work Reported by Carruthers and Evans^17^

**Scheme 4:**
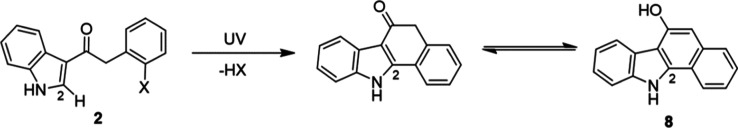
Hypothesized Degradation Mechanism for Halogenated Compounds **2a–d**

From Scheme 3 and Table S2, we find that the two ring systems become increasingly perpendicular to one another as the halogen size increases, with compound **1d** having the largest dihedral angle for both conformers. This observation can be attributed to the increased steric bulk of the larger substituted iodine atom. In all but two compounds, it is noted that the halogen atom is situated in closest proximity to the hydrogen atom bonded to carbon-2 in the lowest energy conformers (Table S2; “measured distance between halogen X and hydrogen H”). Previous work suggests that irradiation of these compounds can cause degradation into conjugated ring structure **7**, with the elimination of compound HX (Scheme 3).^17^ The close proximity of these atoms in the modeled structures suggests that elimination of molecule HX would be possible with the limited energy required for molecular rotation, lowering the energy barrier for elimination. Similarly, the elimination of compound HX from **2a–d** would produce the aromatic four-ring compound **8** (Scheme 4).

**Figure 4:**
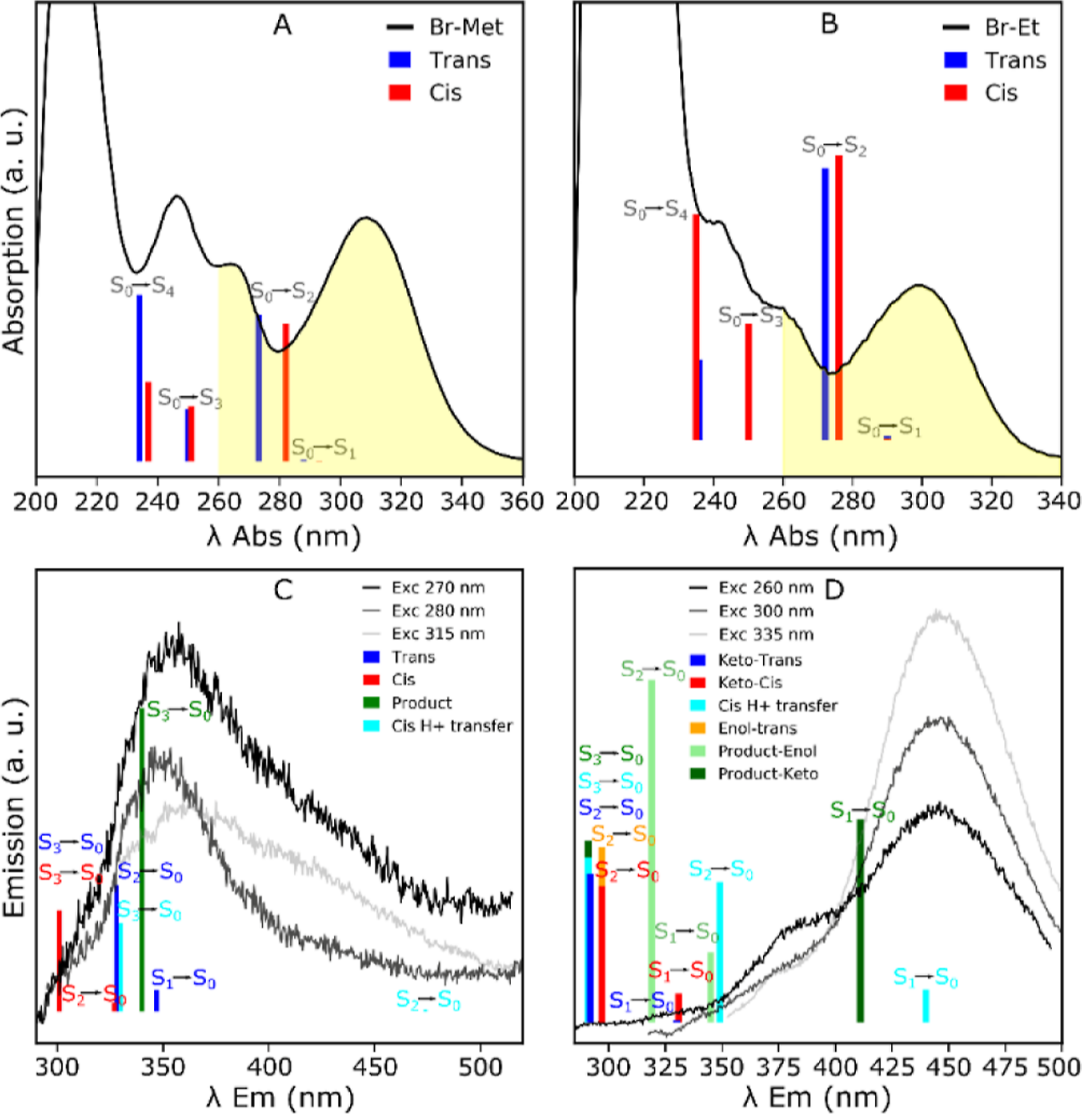
(A,B) Experimental absorption spectra of **1c** and **2c** including the assignment of the main bands. The bars represent the computed vertical excitation energies and their computed oscillator strengths. The yellow area represents the experimental excitation wavelength range used in this work. (C,D) Experimental fluorescence spectra measured in different excitation conditions. The bars represent the vertical emission energies depicted in Scheme 5, and their heights are equal to the corresponding oscillator strengths.

**Scheme 5:**
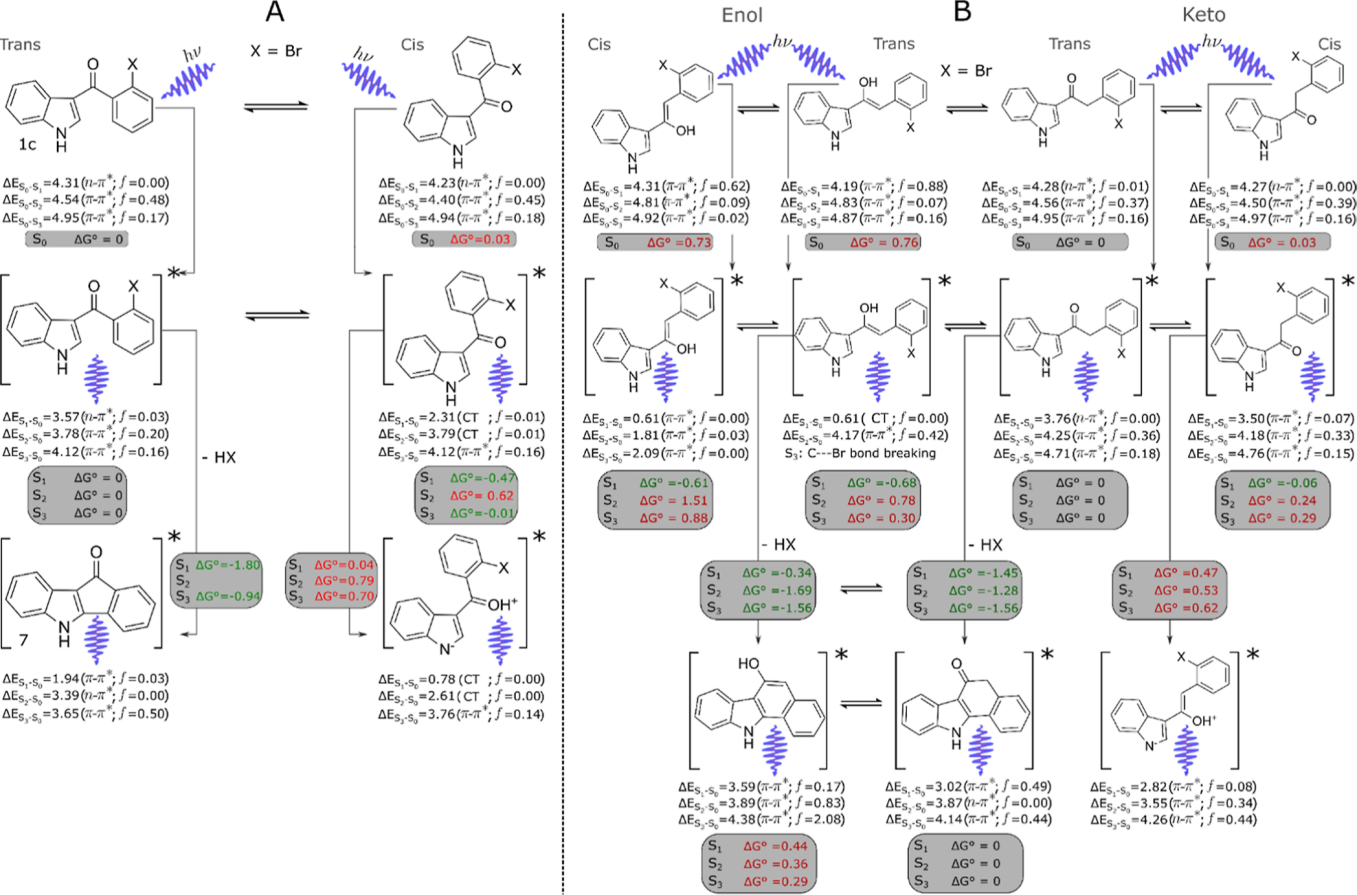
Degradation Processes for **1c** (A) and **2c** (B) in the Ground and Excited States; the Absorption, Emission Energies, and the Nature of the Corresponding Electronic Transitions Are Shown; the Relative Stabilities Were Calculated with Respect to the Most Stable Isomer; the Δ*G* Values for Each Process Connected with Grey Arrows Are Also Displayed; the Asterisk Symbolizes an Electronically Excited Species

Subsequently, we investigated different degradation pathways using DFT and TDDFT calculations. We started optimizing the most stable conformers of **1c** and **2c** in the ground (S_0_) and excited (S_1_, S_2_, and S_3_) electronic states. For these calculations, we considered the cis and trans conformations (with respect to the position of C=O and N–H) at the (TD)-ωB97XD/6-311+G(d) level of theory. We summarized the main processes relevant for this study in Scheme 5A,B. In Figure 4, we show the assignment of the main transitions in the absorption and emission spectra of the brominated molecules **1c** and **2c** and their derivatives. Similar assignments can be done for the other halogenated systems. In both systems, the first three absorption bands are assigned to π–π* S_0_ → S_2,3,4_ transitions (Figure 4A,B).

Transitions to S_1_ in all the energetically accessible structures (Δ*G* ≲ 0.06 eV) show very low probability due to its n−π* character (Scheme 5A,B). The predicted absorption energies are within a range of 0.2–0.5 eV with respect to the experimental values (Scheme 5A,B). Such overestimation is systematically observed in conjugated systems computed with long-range corrected functionals with the default value of the range-separation parameter γ (in this case γ = 0.2*a*_0_^–1^). This is because the HOMO energies tend to be lower, whereas the LUMO’s higher conductivity to a bigger HOMO–LUMO gap energy.^18,19^

Provided the excitation wavelengths used in this work, molecules can be excited up to S_3_ (Figure 4A,B); thus, radiative, reactive and nonradiative deactivation processes to lower states also play a role in the photochemistry. Herein, we analyze the stability of the different species in their excited states. Based on our calculations, we assigned the emission spectra of **1c** and **2c** to the electron transitions of different species (Figure 4C,D).

There are at least four species that can contribute to the emission spectrum of **1c** (Figure 4C). Emission from the **1c** cis species is unlikely as it originates from highly excited electronic states (Kasha’s rule). The cis H^+^ transfer species are not accessible (Scheme 5). Emission from **7** involves the state S_3_, while the process is energetically favorable, relaxation to lower energy states will be faster. Therefore, fluorescence is expected to occur mostly from S_1_ → S_0_ transition of the **1c** trans species.

There are at least six different species that could contribute to emissions after of **2c** (Figure 4D). Nonetheless, in this case, the degradation products (molecule **8**) seem to be the main contributor, with the keto product being the main one based on its greater stability and higher emission oscillator strength (Scheme 5B and Figure 4D). In this case, fluorescence of **8** is predicted to occur from S_1_, as expected from Kasha’s rule. In fact, these are the only two species for which fluorescence from S_1_ is predicted near the observed emission bands. Besides, degradation products are the most stable species in the excited state.

### Characterization of Degradation Products

**Figure 5:**
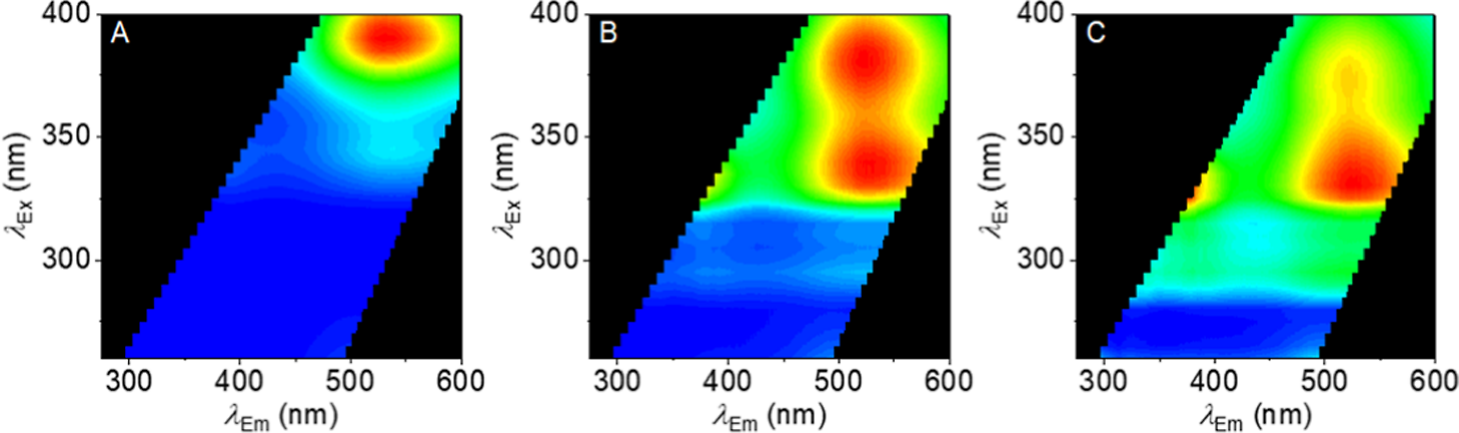
(A–C) FSFs for compound **7** (0.2, 0.05, and 0.025 mg/mL, respectively). Red coloration represents emission with a relative intensity of 1, and blue represents an intensity of 0. Conditions: samples in methanol, 20 °C, 1 cm path length. Data has not had a methanol blank subtracted due to uneven quenching of the methanol signal across the excitation wavelength and sample concentration ranges.

Initial investigation of these degradation systems confirmed the elimination of molecule HX during degradation, as predicted in Scheme 3. Further analysis of the degradation material by mass spectrometry confirmed the presence of **7** in the postdegradation mixture of **1d** and **8** in the degradation mixture of **2d**. To further confirm the degradation product of **1d**, compound **7** was successfully synthesized. By taking FSFs of **7** at 0.2, 0.05, and 0.025 mg/mL (Figure 5), it is evident that the fluorescent nature of this compound is concentration dependent. At a concentration of 0.2 mg/mL, a spectral feature can be identified at λ_Ex_^max^ ≈ 380 nm and λ_Em_^max^ ≈ 500 nm. This spectral feature can also be recognized in the difference map for the degradation of **1d** (Figure 3, panel I), suggesting the evolution of **7** during degradation, among other products.

### Potential for Photochemical Detection in Saliva

SCRA use cannot always be inferred from possession. We have previously shown that SCRA FSFs can be distinguished in saliva, which suggests utility in detecting SCRA use from oral fluid samples. Given the excellent discriminatory potential of UV degradation described above, we sought to explore if enhanced SCRA discrimination via UV irradiation could be similarly useful in samples present in oral fluid. We have elected to use **1d** and **2d** as exemplars owing to these molecules showing the most rapid rate of photochemical degradation.

**Figure 6:**
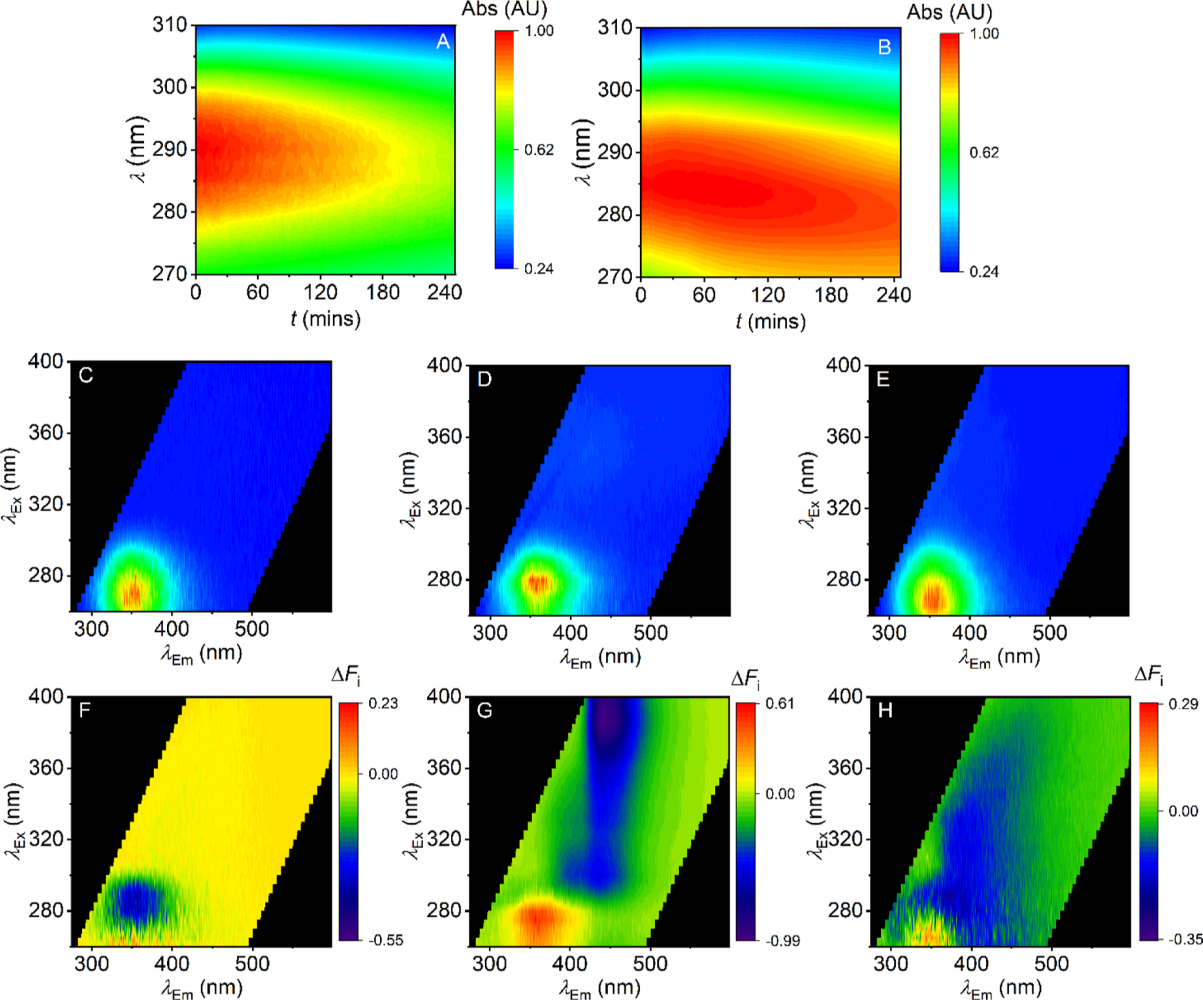
(A,B) Changes in intensity of the absorbance peak of a control saliva sample (A) and a saliva sample with **2d** (B; 1 μg/mL). Red coloration represents emission with a relative intensity of 1. (C–E) FSFs for a control saliva sample (C), a saliva sample with **1d** (D; 1 μg/mL), and a saliva sample with **2d** (E; 1 μg/mL), all taken prior to sample irradiation. Red coloration represents emission with a relative intensity of 1, and blue represents an intensity of 0. (F–H) Differences maps displaying the changes in fluorescent behavior of samples exposed to intense UV light; a control saliva sample (F), a saliva sample containing **1d** (G), and a saliva sample containing **2d** (H). Δ*F*_*i*_ represents the change in signal intensity.

Figure 6 shows the change in absorption for saliva alone (Figure 6A) and saliva with the addition of **2d** (Figure 6B). The major absorption band present at ∼289 nm for saliva is attributable to the high concentration of protein present in human saliva and is typical.^20^ On addition of **2d** (1 μg/mL), the major absorption band appears blue shifted, with a maximum at 285 nm. On irradiation, the absorption of the saliva control sample shows little variance with respect to time. However, on addition of **2d** (Figure 6B), the data show a time-dependent blue-shift of the major absorption band to an absorption maximum of ∼279 nm, over 240 min. These data suggest that there are photochemically driven changes to chromophores (presumably **2d**) that can be captured by absorption in a saliva matrix, but not that are observable in saliva alone. These data are then a positive indicator that FSFs might be able to capture the fluorescence signatures of photochemical degradation of SCRAs in saliva.

Figure 6C–E shows the FSFs for saliva (6C), saliva containing **1d** (6D), and saliva containing **2d** (6E). Figure 5F–H show the difference maps after irradiation for 30 min. These data show a loss in emission attributable to protein aromatic amino acids at λ_Em_ ≈ 350 nm for the saliva only sample (Figure 6F), potentially reflecting degradation of these chromophores, which is as expected from an abundance of photochemical degradation studies on proteins.^21−24^ Conversely, the presence of both **1d** and **2d** show an increase in emission around λ_Em_ ≈ 350 nm (Figure 6G,H) accompanied by a diffuse loss in emission elsewhere in the FSF.

A range of concentrations have been reported for relevant molecular concentrations in saliva postsmoking, including a maximum of 22,370 μg/L for THC^25^ and 35 μg/L for JWH-018.^26^ Given the huge potential range of biologically meaningful concentrations, we have opted to use ∼1 μg/mL. Figure S4 shows the FSFs for MDMB-4en-PINACA in saliva/methanol at concentrations of 10, 50, and 250 ng/mL. Variations observed in the 330–400 nm excitation region are likely due to differences in diet preceding saliva collection on those days. We note the potential for convolving species from diet/other consumption. However, we find that FSFs arising from human saliva are remarkably consistent, dominated almost entirely by the peak at λ_Em_ ∼ 280 nm.^7^ Although detection at a concentration of 50 ng/mL produces a clear fingerprint, at 10 ng/mL, the SCRA signal is obscured. This can be overcome by subtracting the specific saliva sample used on that day, indicating the potential for optimization of the data subtraction method for increased sensitivity. These data show the potential for SCRA detection below 50 ng/mL in a saliva/methanol solution.

**Figure 7:**
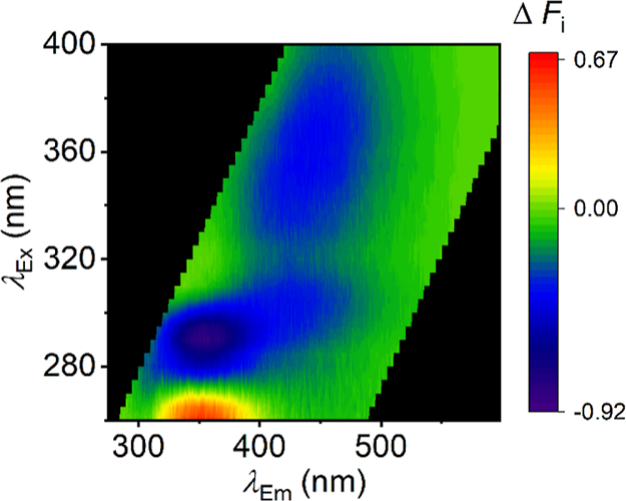
Difference map of FSFs (pre- and postirradiation) for saliva containing combusted SCRA, AM-694. Δ*F*_*i*_ represents the change in signal intensity.

These data show the potential of photochemical degradation combined with FSF detection for SCRA analogues at a physiologically anticipated concentration. Given these findings, we wished to explore whether combusted SCRA (mimicking the effects of smoking SCRA material) could be similarly detected from a saliva-only control. We have previously developed a smoking simulator for generating realistically combusted material.^27^ For the purposes of this study, we have generated combusted AM-694 (Figure 1), the SCRA on which the analogues used in this study are based. Figure 7 shows the FSF difference map of pre- and postirradiated combusted AM-694 in saliva, as in Figure 6. This difference map shows spectral changes on irradiation similar to the analogues discussed above (Figure 6F–H), with an increase in emission located around λ_Em_ ≈ 350 nm and a diffuse decrease in emission across the rest of the FSF. We note that the decrease in emission (blue coloration in Figure 7) is highly reminiscent of the FSF of combusted AM-694 recorded previously,^7^ which suggests, similar to our photochemical studies mentioned above, that irradiation leads to the loss of the parent SCRA FSF. Combined with our model series data, these data imply that the presence of SCRA in saliva ‘protects’ the emission centered at λ_Em_ ≈ 350 nm, versus in the absence of the SCRA. This might be a definable characteristic of the presence of SCRA in saliva.

## Conclusions

The vast majority of SCRAs are built on a similar scaffold, with a high-quantum yield fluorophore at the “core” position. This is based on the ready availability of indole/indazole precursor materials and the chemical tractability of chemical substitution. We have previously taken advantage of the sensitivity of indole/indazole fluorescence to show that the excitation-emission matrices (FSFs in the manuscript) of these compounds are both distinctive of SCRAs and also of different SCRAs. Our present work highlights that this sensitivity arises not just due to immediate substituents on the chromophore but also at positions remote from the chromophore. Moreover, our DFT calculations suggest this arises from a shift in the distribution of conformational and excited states (electronic, emissive states) that each SCRA can access.

Our data shows that, at least for SCRAs, FSFs are a powerful analytical detection methodology. However, for application in the field, one requires extreme sensitivity and robustness of detection, not least because detection of SCRAs has profound legal and social consequences. We therefore sought to combine the FSF detection approach with monitoring of the photochemical reactivity of SCRAs. We find that photochemical discrimination is specific for individual SCRA analogues and that this can even be achieved from street material in saliva. We term this hybrid approach, which distinguishes the SCRA from a pre- and postirradiation FSF difference map (such as Figure 6), photochemical fingerprinting.

**Figure 8:**
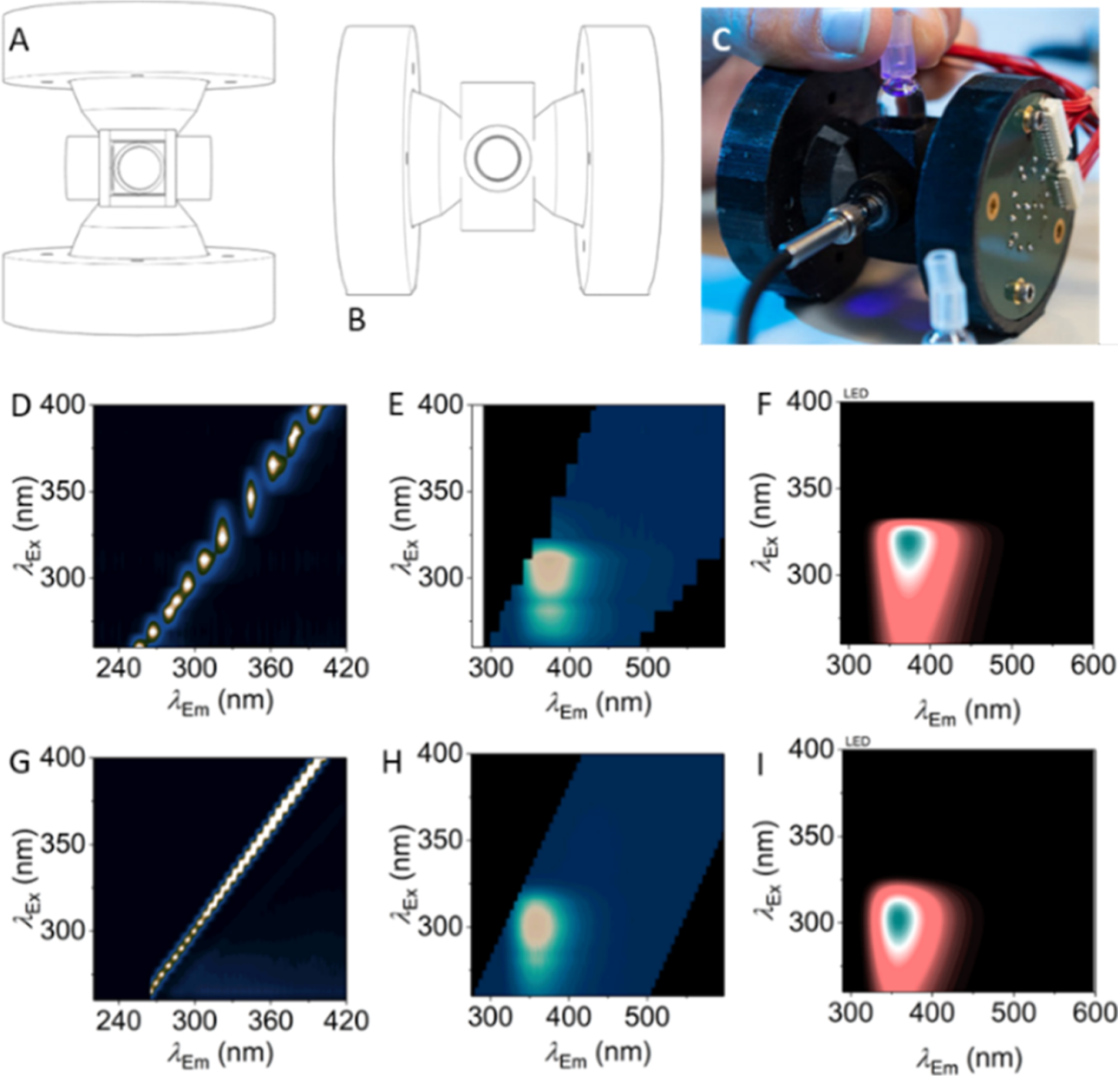
Design and performance of a prototype portable multiexcitation wavelength fluorimeter. (A,B) Top and side on views of a 3D printed housing for a custom cuvette holder, light collecting optics, and LED array’s mounted on custom PCBs. Emitted light collected via a commercially available portable spectrometer. (C) Photo of the prototype in operation. (D) Characterization of LEDs showing the peak positions and spectral bandwidths compared to (E), the excitation from a monochromator-based, bench-top fluorimeter. FSF of a SCRA (1 μg/mL 5F-MDMB-PINACA) using (F) the LED-based device and (G) the bench-top fluorimeter. (H,I) Are the resultant numerical models (eq 1) arising from (F,G), respectively. These data shown very close agreement between the parameters extracted from eq 1.

Photochemical fingerprinting has the advantage that it can be readily incorporated into a portable detection system. We have previously pointed to the success of portable fluorimeters built using UV LEDs as the excitation source. Recent advances in LEDs in this spectral region (<400 nm) mean they are bright (∼mW tunable), stable (thousands of hours), and have low spectral bandwidths (∼12 nm). Figure 8 shows a portable device built for point-of-care SCRA detection via FSFs. It would be trivial to irradiate the sample with one of the LEDs (300 nm in this case) to drive photochemical degradation. We envisage that with bright enough or multiple LED sources, a photochemical fingerprint could be rapidly produced at a range of different excitation wavelengths, further enhancing the detection potential. Moreover, as we have shown with BZO-HEXOXIZID, this approach could enable FSF detection with molecules that, prior to irradiation, have low quantum yields, expanding the detection scope to other drugs of abuse.

## Materials and Methods

All glassware was flame-dried under vacuum, and all moisture sensitive reactions and reagent transfers were carried out under nitrogen.

### Synthesis of Compounds **1a–d**

In a 250 mL two-necked round-bottomed flask, indole **3** (4.27 mmol) was dissolved in dry DCM (42.5 mL) under an inert atmosphere. After cooling in an ice bath for 10 min, Et_2_AlCl (1 mol/L in hexane, 6.4 mL) was syringed into the flask via slow, dropwise addition. The mixture was stirred in the ice bath for 30 min before the slow dropwise addition of the corresponding acyl halide **4a–d** (6.4 mmol) diluted in 10 mL of DCM. The resulting mixture was stirred at room temperature overnight (16 h), and then quenched with 20 mL of sat. aq. NH_4_Cl.

An off-white suspended solid formed in the reaction mixture due to aluminum salts precipitating out of solution upon quenching. This was gravity filtered to produce a clear filtrate that was extracted with additional DCM (2 × 35 mL). The aqueous layer containing the majority of the aluminum salts was discarded. The DCM layer was washed with distilled water (3 × 25 mL), dried (MgSO_4_), and evaporated to afford the crude product. This was purified using silica gel column chromatography (petroleum ether: ethyl acetate 4:1, *R*_f_ = 0.18) to produce **1a–d**, which was characterized with NMR and IR spectroscopy, melting point, and mass spectrometry (see Supporting Information).

### Synthesis of Acetyl Chloride **6a–d**

A mixture of 2-halogenated acetic acid **5a–d** (10 mmol) and SOCl_2_ (25 mL) was stirred at 100 °C for 3 h, under reflux. 10 mL of toluene was added, and any excess SOCl_2_ was removed via distillation at 105 °C. Toluene was then removed under reduced pressure to afford 2-halogenated acetyl chloride **6a–d**.

### Synthesis of Compounds **2a–d**

The same synthesis method, as was used for compounds **1a–d**, was undertaken using the corresponding phenylacetyl chloride **6a–d**. However, the crude product was instead purified via trituration with ethyl acetate and recrystallized from chloroform to give **2a–d**. This was characterized with NMR and IR spectroscopy, melting point, and mass spectrometry (see Supporting Information).

### Computational Study

To account for molecular flexibility, comprehensive conformational searches were performed for all eight compounds (**1a–d** and **2a–d**) using Schrödinger’s MacroModel (Ver 11.3).^14^ The OPLS3e force field and PRCG minimization method were chosen for conformational searches, and a mixed torsional/low-mode sampling approach was adopted. All structures were further optimized using DFT, with geometry optimizations being performed in Gaussian 16 (Rev. A.03).^15^ Calculations were completed at the B3LYP-D3(BJ)/def2svp level of theory. Grimme’s D3 dispersion correction with Becke–Johnson damping was included to better account for weak intermolecular interactions, as previously utilized in the literature. Implicit solvation using IEF-PCM was included in all calculations, with methanol as the chosen solvent (dielectric constant ε = 32.613). The temperature (298 K)- and concentration (1 mol dm^–3^)-corrected quasiharmonic free energy of each conformation was obtained using the GoodVibes.^16^

Excited state calculations were performed for the most stable conformers of **1c**, **2c**, and AM-694 molecules. We consider the molecules with conformations cis and trans with respect to the positions of the carbonyl and the amine groups (conformers bromomethanone_1, bromomethanone_2, bromoethanone_1, bromoethanone_2, and AM-694_1, see Table S2). The optimizations of ground and the S_1_, S_2_, and S_3_ states were performed at the TD-ωB97XD/6-311+G(d) level of theory in methanol with the IEFPCM model. Every stable geometry was tested as a true minimum by a vibrational frequency analysis, obtaining zero imaginary frequencies. The thermodynamic functions Δ*H*, Δ*S*, and Δ*G* were computed for every sable geometry obtained at 298 K within the harmonic oscillator and rigid rotor approximations, as implemented in Gaussian 16.^15^ The absorption and emission energies were computed as the vertical transition from the equilibrium structure of the electronic state from which the transition occurs.

### Absorption and Fluorescence Spectra and Photodegradation

Fluorescence readings were collected using a PerkinElmer LS50B luminescence spectrometer (PerkinElmer, Waltham, MA, USA) with an attached water bath for temperature regulation. Sample and background measurements were taken at 20 °C. The excitation and emission slit widths were varied between 2.5 and 12 nm depending on the signal. For each measurement, a corresponding background reading was directly subtracted, particularly to remove contributions from Raman scattering. The FSFs shown have had the signal contributions from excitation light and second order scattering removed.

Absorbance measurements were taken using a Varian Cary 50 Scan UV-vis photometer. Absorbance was measured from 800 to 200 nm at 1 nm intervals with a scan rate of 600 nm/min.

Sample degradation was carried out using a M300L4—300 nm, 26 mW Thor Labs LED. The LED was in a fixed position relative to the cuvette holder used in the irradiation step, keeping the intensity of the light delivered consistent. Samples were fully contained during degradation, so volume and sample concentration were unchanged.

The synthetic strategy and purification process of these samples are described above. Samples were dissolved in HPLC methanol >99.9% purity (Sigma-Aldrich, St. Louis, MO, USA).

Oral fluid samples were collected from volunteers who confirmed no legal or illegal drug use in the preceding month. Saliva samples were centrifuged for 15 min at 4 °C, to separate solid material before being passed through a 0.44 μm syringe-driven filter.

## Supporting Information Available

Parameters resulting from fitted FSFS and computational calculations; photodegradation fingerprints for **1a–d**; and analytical characterization from syntheses (PDF)
