# Supplementary material for: Photochemical Fingerprinting Is a Sensitive Probe for the Detection of Synthetic Cannabinoid Receptor Agonists; toward Robust Point-of-Care Detection
Source: Anal Chem. 2023 Jan 4;95(2):703–13. doi: 10.1021/acs.analchem.2c02529 (PMC9850351; doi:10.1021/acs.analchem.2c02529)
Supplement: Supplementary file 1 — ac2c02529_si_001.pdf [file ac2c02529_si_001.pdf]

Supporting Information

Photochemical fingerprinting is a sensitive probe for the detection of synthetic cannabinoid receptor agonists; towards robust point-of-care detection

Rachael C. Andrews,[1,2‡] Benedict May,[3‡] Federico J. Hernández,[4‡] Gyles E. Cozier,[3] Piers A. Townsend,[5] Oliver B. Sutcliffe,[6] Tom S. F. Haines,[7] Tom P. Freeman,[8] Jennifer Scott,[9] Stephen M. Husbands,[9] Ian S. Blagbrough,[9] Richard W. Bowman,[10] Simon E. Lewis,[1] Matthew N. Grayson,[1,2*] Rachel Crespo-Otero,[4*] David R. Carbery[1*] and Christopher R. Pudney[2,3,11*]

[1]Department of Chemistry, [2]Centre for Sustainable Chemical Technology, [3]Department of Biology and Biochemistry, University of Bath, Bath BA2 7AY, UK. [4] Department of Chemistry, Queen Mary University of London, London, E1 4NS, UK. [5]School of Applied Sciences, University of the West of England, Bristol, BS16 1QY, [6] MANchester DRug Analysis & Knowledge Exchange (MANDRAKE), Department of Natural Sciences, Manchester Metropolitan University, Manchester, M15 5GD. [7]Department of Computer Science, [8]Department of Psychology. [9]Department of Pharmacy and Pharmacology, [10]School of Physics and Astronomy, University of Glasgow, Glasgow, G12 8QQ, [11]Centre for Therapeutic Innovation, University of Bath, Bath BA2 7AY, UK.

Contents

Page S3: Supplementary materials and methods (analytical data for synthesised compounds **1a-d** and **2a-d**

Table S1. Mean ± Standard Deviation of the position parameters taken from the models fit to FSFs of the SCRA analogues (Eq1).

Table S2. Data calculated via DFT calculations for the lowest energy conformers of compounds **1a-d** and **2a-d**. Quasiharmonic energies have been calculated at a temperature of 298.15 K and a concentration of 1 moldm^{-3}. The Boltzmann weighting of each conformer in the population are shown, with the distance between the halogen atom and the hydrogen atom on carbon-2, and the angle between the two planar ring systems (for compounds **1a-d**). Highlighting shows the conformers with the lowest energy (orange), second lowest energy (yellow), and smallest distance between C-2 hydrogen and halogen atom (green).

Figure S1. FSFs for (A) MDMB-4en-PICA, (B) MDMB-4en-PINACA, (C) MDMB-FUBICA, (D) MDMB-FUBINACA and (E) MDA-19 shown prior and post degradation. Red colouration represents emission with a relative intensity of one, and blue represents an intensity of zero. Inserts for (D) are coloured 0 to 0.025 relative intensity (blue to red) to highlight the minor changes observed. Also shown is the Prior - Post difference heat map. Conditions: 250 ng/mL in methanol, 20 °C, 2 hours of irradiation with 300 nm LED.

Figure S2: Rate constants for the degradation of the maximum absorbance peak for **1a-d** (Meth 310 nm) and **2-d** (Eth 400 nm). Note that different spectral bands are selected to prevent spectral convolution in the kinetic data as described in the main text.

Figure S3: FSFs - **2a** for (Panels A-C), **2b** for (Panels D-F), **2c** for (Panels G-I) and **2d** for (Panels J-L) - collected pre and post sample photodegradation. Red coloration represents emission with a relative intensity of one, blue an intensity of zero, and a heat map showing the differences in emission intensity.

Scheme S1: Degradation processes for AM-694 in the ground and excited states. The absorption, emission energies and the nature of the corresponding electronic transitions are shown. The relative stabilities were calculated with respect to the most stable isomer. The ΔG values for each process connected with grey arrows are also displayed. The asterisk symbolises electronically excited species.

Figure S4. FSFs for (A) average of saliva/methanol samples used, (B-D) 10, 50 and 250 ng/mL MDMB-4en-PINACA in saliva/methanol and (E) 250 ng/mL MDMB-4en-PINACA in methanol only. Conditions: 1 mL cuvette, 20 °C, 1:2 v/v saliva: methanol. Red colouration represents emission with a relative intensity of one, and blue represents an intensity of zero. (F-H) Difference heat maps of 10, 50 and 250 ng/mL MDMB-4en-PINACA in saliva minus averaged saliva, coloured from -0.05 to 0.228 relative intensity (blue to red).

Figure S5-24: NMR spectra for **1a-d**, **2a-d** and **7**

Page S21-22: HRMS Analysis of Compounds **1a-d, 2a-d, 7**

Page S23-27: IR Confirmation of Compounds **1a-d, 2a-d, 7**

Page 28-88: Coordinates for **1a-d** and **2a-d** from DFT calculations (B3LYP-D3(BJ)/Def2SVP Molecular Geometry in Cartesian Coordinates)

Supplementary Materials and Methods

3-(2-fluorobenzoyl)-1H-indole ***1a***

Sand-coloured solid (0.3354 g, 1.40 mmol, 32.8 %); Mp 188.5 – 193.8 °C; ^{1}H NMR (500 MHz, DMSO-*d6*) δ 12.13 (s, 1H), 8.24 – 8.16 (m, 1H), 7.79 (s, 1H), 7.61 – 7.56 (m, 2H), 7.54 – 7.50 (m, 1H), 7.38 – 7.31 (m, 2H), 7.30 – 7.23 (m, 2H). ^{13}C NMR (126 MHz, DMSO-*d6*) δ 186.1, 158.7 (d, J = 247.4 Hz), 136.9, 131.8 (d, J = 8.3 Hz), 129.7, 129.2, 129.1, 125.5, 124.4, 124.4, 123.3, 122.2, 121.1, 116.2 (d, J = 14.7 Hz), 116.0, 112.4 ppm; ^{19}F NMR (376 MHz, DMSO-*d6*) δ -115.71 (dt, J= 12.3, 7.1 Hz); IR (ATR) 3178.03 (N-H), 1613.49 (C=O) cm^{-1}; *m/z:* $[M-H]^-$ Calculated for $C_{15}H_{10}NOF$ 239.0746; Found 239.0740.

3-(2-chlorobenzoyl)-1H-indole ***1b***

Sand-coloured solid (0.5325 g, 2.08 mmol, 48.8 %); Mp 178.1 – 182.8 °C ; ^{1}H NMR (500 MHz, DMSO-d_6) δ 12.11 (s, 1H), 8.16-8.12 (m, 1H), 7.64 (s, 1H), 7.59–7.44 (m, 5H), 7.30-7.21 (m, 2H) ppm; ^{13}C NMR (126 MHz, DMSO-d_6) δ 188.2, 140.3, 137.0, 136.9, 130.7, 129.7, 129.6, 128.7, 127.0, 125.4, 123.3, 122.2, 121.1, 116.0, 112.5 ppm; IR (ATR) 3226.72 (N-H), 1600.74 (C=O) cm^{-1}; *m/z*: $[M-H]^-$ Calculated for $C_{15}H_{10}NOCl$ 255.0451; Found 255.0446

3-(2-bromobenzoyl)-1H-indole ***1c***

Sand-coloured solid (0.8933 g, 2.98 mmol, 69.7 %); Mp 174.6 – 177.0 °C; ^{1}H NMR (500 MHz, DMSO-d_6) δ 12.11 (s, 1H), 8.13 (d, J = 7.2 Hz, 1H), 7.73 (d, J = 8.0 Hz, 1H), 7.62 (s, 1H), 7.53-7.47 (m, 3H), 7.46-7.42 (m, 1H), 7.26 (pd, J = 7.1, 1.3 Hz, 2H) ppm; ^{13}C NMR (126 MHz, DMSO-d_6) δ 189.0, 142.3, 137.0, 136.9, 132.8, 130.8, 128.6, 127.5, 125.4, 123.3, 122.2, 121.1, 118.6, 115.7, 112.5 ppm; IR (ATR) 3150.78 (N-H), 1597.66 (C=O) cm^{-1}; *m/z*: $[M-H]^-$ Calculated for $C_{15}H_{10}NOBr$ 298.9946; Found 298.9938.

3-(2-iodobenzoyl)-1H-indole ***1d***

Sand-coloured solid (0.2876 g, 0.83 mmol, 19.4 %); Mp 184.0 – 185.5 °C; ^{1}H NMR (500 MHz, DMSO-d_6) δ 12.09 (s, 1H), 8.12 (d, J = 7.1 Hz, 1H), 7.98-7.93 (m, 1H), 7.58 (d, J = 2.0 Hz, 1H), 7.56-7.48 (m, 2H), 7.43 (dd, J = 7.5, 1.6 Hz, 1H), 7.31-7.21 (m, 3H) ppm; ^{13}C NMR (126 MHz, DMSO-d_6) δ 191.0, 146.1, 139.1, 137.0, 136.9, 130.7, 127.9, 127.8, 125.5, 123.3, 122.2, 121.2, 115.2, 112.5, 93.1 ppm; IR (ATR) 3146.12 (N-H), 1595.99 (C=O) cm^{-1}; *m/z*: $[M-H]^-$ Calculated for $C_{15}H_{10}NOI$ 346.9807; Found 346.9796.

2-(2-fluorophenyl)-1-(1H-indol-3-yl)ethanone ***2a***

Pale pink solid (0.3442 g, 1.35 mmol, 31.9 %); Mp 194.5 – 195.0 °C; ^{1}H NMR (500 MHz, DMSO-d_6) δ 12.01 (s, 1H), 8.49 (s, 1H), 8.15 (dd, J=7.8, 1.3 Hz, 1H), 7.49 (d, J = 7.8 Hz, 1H), 7.40-7.26 (m, 2H), 7.26-7.12 (m, 4H), 4.28 (s, 2H); ^{13}C NMR (126 MHz, DMSO-d_6) δ 191.03, 161.72, 159.78, 136.61, 134.25, 132.29, 132.25, 128.52, 128.46, 125.44, 124.11, 124.08, 123.47, 123.35, 122.84, 121.77, 121.21, 115.80, 115.00, 114.83, 112.13, 39.07; ^{19}F NMR (376 MHz, DMSO-d_6) δ -116.97 (dt, J = 10.5, 6.8 Hz); IR (ATR) 3176.25 (N-H), 1628.18 (C=O) cm^{-1}; m/z: $[M-H]^-$ Calculated for $C_{16}H_{12}NOF$ 253.0903; Found 253.0902.

2-(2-chlorophenyl)-1-(1H-indol-3-yl)ethanone ***2b***

Sand coloured solid (0.1114 g, 0.41 mmol, 9.62 %); Mp 215.4 – 216.4 °C; ^{1}H NMR (500 MHz, DMSO-d_6) δ 12.00 (s, 1H), 8.51 (s, 1H), 8.14 (d, J = 7.8 Hz, 1H), 7.52-7.37 (m, 3H), 7.30 (qt, J = 7.5, 3.8 Hz, 2H), 7.20 (dt, J=22.7, 7.1 Hz, 2H), 4.40 (s, 3H); ^{13}C NMR (126 MHz, DMSO-d_6) δ 190.92, 136.58, 134.52, 134.09, 133.81, 132.50, 128.90, 128.31, 126.91, 125.43, 122.82, 121.75, 121.22, 115.95, 112.13, 43.48; IR (ATR) 3214.02 (N-H), 1630.46 (C=O) cm^{-1}; m/z: $[M-H]^-$ Calculated for $C_{16}H_{12}NOCl$ 269.0607; Found 269.0607.

2-(2-bromophenyl)-1-(1H-indol-3-yl)ethanone ***2c***

Sand coloured solid (0.0950 g, 0.30 mmol, 7.04 %); Mp 225.2 – 226.7 °C; ^{1}H NMR (500 MHz, DMSO-d_6) δ 12.01 (s, 1H), 8.51 (s, 1H), 8.14 (d, J = 7.6 Hz, 1H), 7.61 (dd, J = 8.0, 1.0 Hz, 1H), 7.49 (d, J = 8.0 Hz, 1H), 7.43-7.32 (m, 2H), 7.26-7.15 (m, 3H), 4.42 (s, 2H); ^{13}C NMR (126 MHz, DMSO-d_6) δ 190.85, 136.58, 136.31, 134.05, 132.55, 132.15, 128.51, 127.45, 125.43, 124.80, 122.80, 121.74, 121.22, 116.00, 112.13, 45.92 ; IR (ATR) 3213.34 (N-H), 1626.33 (C=O) cm^{-1}; m/z: $[M-H]^-$ Calculated for $C_{16}H_{12}NOBr$ 313.0102; Found 313.0101.

2-(2-iodophenyl)-1-(1H-indol-3-yl)ethanone ***2d***

Sand coloured solid (0.0780 g, 0.22 mmol, 5.07 %); Mp 228.7 – 229.9 °C ; ^{1}H NMR (500 MHz, $CDCl_3$) δ 12.00 (s, 1H), 8.52 (d, J = 3.1 Hz, 1H), 8.14 (d, J = 7.7 Hz, 1H), 7.88 – 7.83 (m, 1H), 7.49 (d, J = 8.1 Hz, 1H), 7.41 – 7.33 (m, 2H), 7.20 (dtd, J = 22.1, 7.2, 1.2 Hz, 2H), 7.02 (ddd, J = 8.0, 6.2, 2.9 Hz, 1H), 4.42 (s, 2H) ppm; ^{13}C NMR (126 MHz, DMSO-d_6) δ 191.40, 140.35, 139.16, 137.05, 134.51, 132.07, 128.91, 128.55, 125.91, 123.27, 122.21, 121.72, 116.68, 112.60, 102.60, 50.79 ppm; IR (ATR) 3212.62 (N-H), 1629.81 (C=O) cm^{-1}; *m/z*: $[M-H]^-$ Calculated for $C_{16}H_{12}NOI$ 360.9964; Found 360.9964.

Tables

Table S1. Mean ± Standard Deviation of the position parameters taken from the models fit to FSFs of the SCRA analogues (Eq1).

Molecule	$\lambda_{Em}1$ (nm)	$\lambda_{Ex}1$ (nm)	$\lambda_{Em}2$ (nm)	$\lambda_{Ex}2$ (nm)
**1a**	403 ± 0.9	291 ± 0.3	348 ±0.3	281 ± 0.1
**1b**	356 ± 2.5	280 ± 0.5	417 ± 14	360 ± 7
**1c**	347 ± 0.7	281 ± 0.3	410 ± 2.3	343 ± 3.3
**1d**	347 ± 1.8	279 ± 0.6	411 ± 9	263 ± 1.2
**2a**	453 ± 0.6	336 ± 0.6	453 ± 5.0	264 ± 1.0
**2b**	451 ± 1.4	323 ± 0.7	462 ± 0.5	255 ± 3.8
**2c**	448 ± 0.1	335 ± 0.7	-	-
**2d**	384 ± 0.3	282 ± 1.4	-	-

Table S2. Data calculated via DFT calculations for the lowest energy conformers of compounds **1a-d** and **2a-d**. Quasiharmonic energies have been calculated at a temperature of 298.15 K and a concentration of 1 moldm^{-3}. The Boltzmann weighting of each conformer in the population are shown, with the distance between the halogen atom and the hydrogen atom on carbon-2, and the angle between the two planar ring systems (for compounds **1a-d**). Highlighting shows the conformers with the lowest energy (orange), second lowest energy (yellow), and smallest distance between C-2 hydrogen and halogen atom (green).

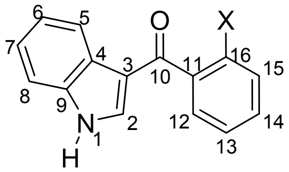

Structure	qh-G(T)_SPC / hartree	qh-G(T) / kcal mol^{-1}	Boltzmann Weight	Distance between H and X / Å	Dihedral / degrees
Fluoromethanone_1	-806.759	-506249	0.609	2.590	49.9
Fluoromethanone_2	-806.758	-506249	0.305	4.592	130.0
Fluoromethanone_3	-806.757	-506248	0.066	4.692	48.5
Fluoromethanone_4	-806.756	-506247	0.019	5.399	132.6
Chloromethanone_1	-1167.071	-732348	0.815	3.263	68.2
Chloromethanone_2	-1167.069	-732347	0.185	4.859	-67.6
Bromomethanone_1	-3281.958	-2059460	0.776	3.493	72.6
Bromomethanone_2	-3281.957	-2059459	0.224	4.904	71.1
Iodomethanone_1	-1005.578	-631010	0.770	3.741	-74.7
Iodomethanone_2	-1005.577	-631009	0.230	4.983	-73.6
Fluoroethanone_1	-846.028	-530890	0.580	2.306	-
Fluoroethanone_2	-846.024	-530888	0.020	6.732	-
Fluoroethanone_3	-846.026	-530889	0.099	4.049	-
Fluoroethanone_4	-846.027	-530890	0.273	5.032	-
Fluoroethanone_5	-846.025	-530889	0.026	5.366	-
Fluoroethanone_6	-846.022	-530887	0.002	3.936	-
Chloroethanone_1	-1206.340	-756990	0.377	2.675	-
Chloroethanone_2	-1206.340	-756990	0.442	4.713	-
Chloroethanone_3	-1206.337	-756988	0.019	7.162	-
Chloroethanone_4	-1206.339	-756989	0.112	4.241	-
Chloroethanone_5	-1206.337	-756988	0.033	5.479	-
Chloroethanone_6	-1206.337	-756988	0.013	6.067	-
Chloroethanone_7	-1206.336	-756987	0.005	5.415	-
Chloroethanone_8	-1206.334	-756986	0.001	3.899	-
Bromoethanone_1	-3320.142	-2083420	0.545	2.832	-
Bromoethanone_2	-3320.140	-2083419	0.036	7.322	-
Bromoethanone_3	-3320.141	-2083420	0.087	5.517	-
Bromoethanone_4	-3320.142	-2083420	0.204	4.347	-
Bromoethanone_5	-3320.140	-2083420	0.040	6.186	-
Bromoethanone_6	-3320.141	-2083420	0.087	5.517	-
Bromoethanone_7	-3320.136	-2083417	0.001	3.966	-
Bromoethanone_8	-3320.136	-2083417	0.001	3.967	-
Iodoethanone_1	-1044.064	-655160	0.179	3.046	-
Iodoethanone_2	-1044.061	-655158	0.015	7.522	-
Iodoethanone_3	-1044.063	-655159	0.052	5.580	-
Iodoethanone_4	-1044.063	-655160	0.088	4.459	-
Iodoethanone_5	-1044.065	-655161	0.664	4.592	-
Iodoethanone_6	-1044.057	-655156	0.000	4.029	-
Iodoethanone_7	-1044.057	-655156	0.000	4.029	-

Figures

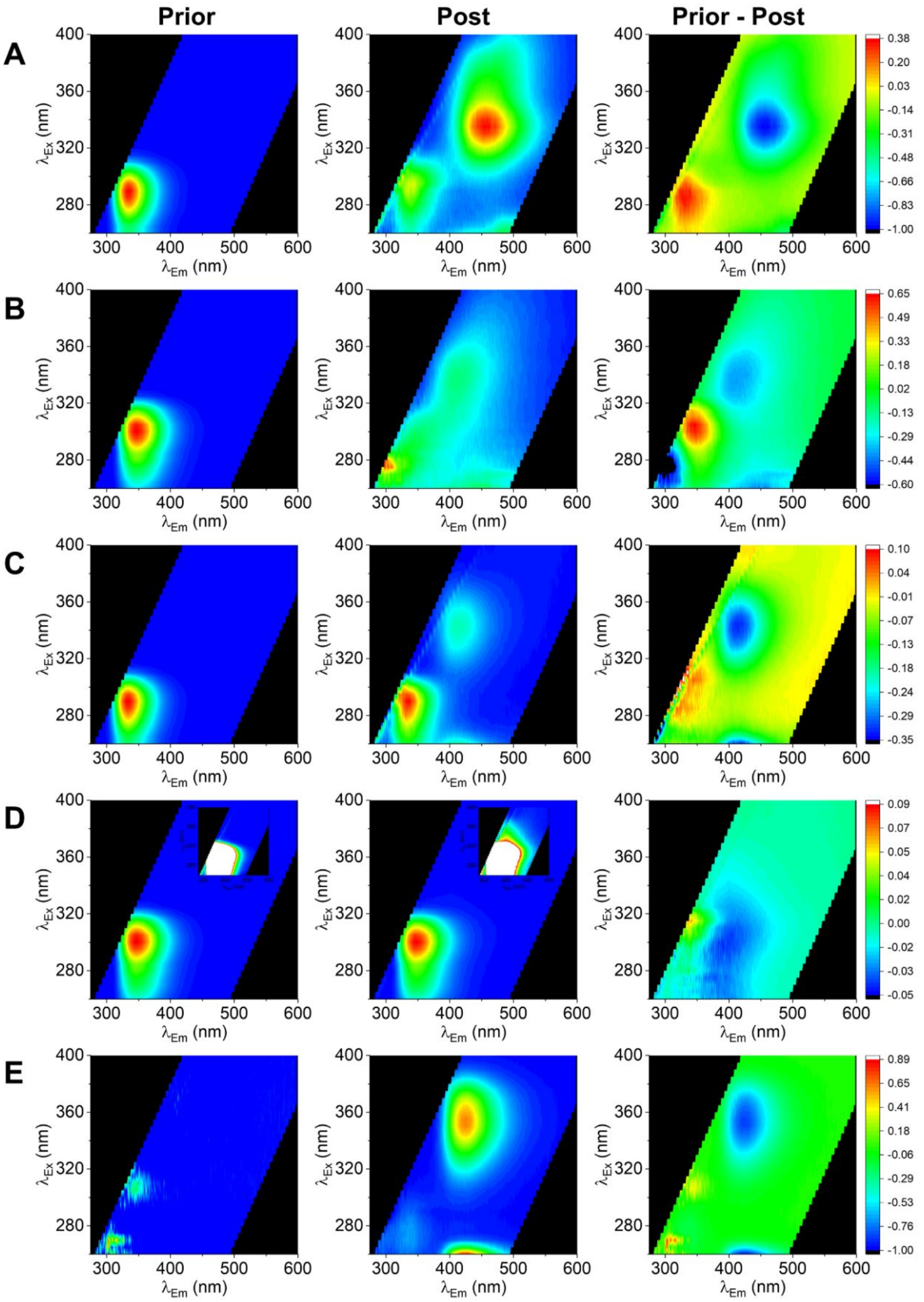

Figure S1. FSFs for (A) MDMB-4en-PICA, (B) MDMB-4en-PINACA, (C) MDMB-FUBICA, (D) MDMB-FUBINACA and (E) MDA-19 shown prior and post degradation. Red colouration represents emission with a relative intensity of one, and blue represents an intensity of zero. Inserts for (D) are coloured 0 to 0.025 relative intensity (blue to red) to highlight the minor changes observed. Also shown is the Prior - Post difference heat map. Conditions: 250 ng/mL in methanol, 20 °C, 2 hours of irradiation with 300 nm LED.

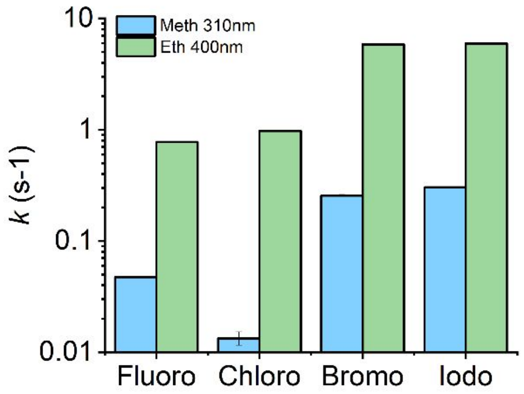

Figure S2: Rate constants for the degradation of the maximum absorbance peak for **1a-d** (Meth 310 nm) and **2-d** (Eth 400 nm). Note that different spectral bands are selected to prevent spectral convolution in the kinetic data as described in the main text.

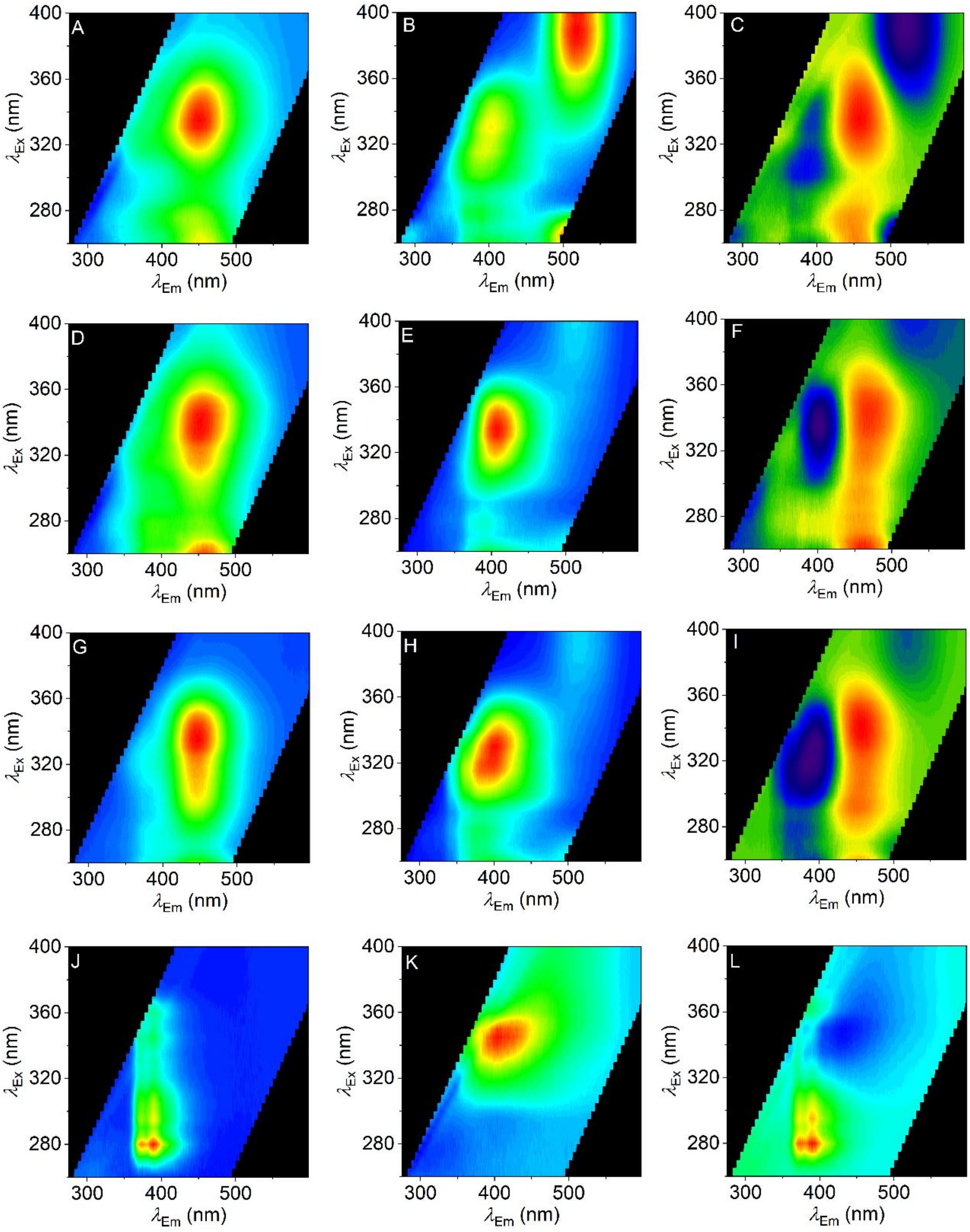

Figure S3: FSFs - **2a** for (Panels A-C), **2b** for (Panels D-F), **2c** for (Panels G-I) and **2d** for (Panels J-L) - collected pre and post sample photodegradation. Red coloration represents emission with a relative intensity of one, blue an intensity of zero, and a heat map showing the differences in emission intensity.

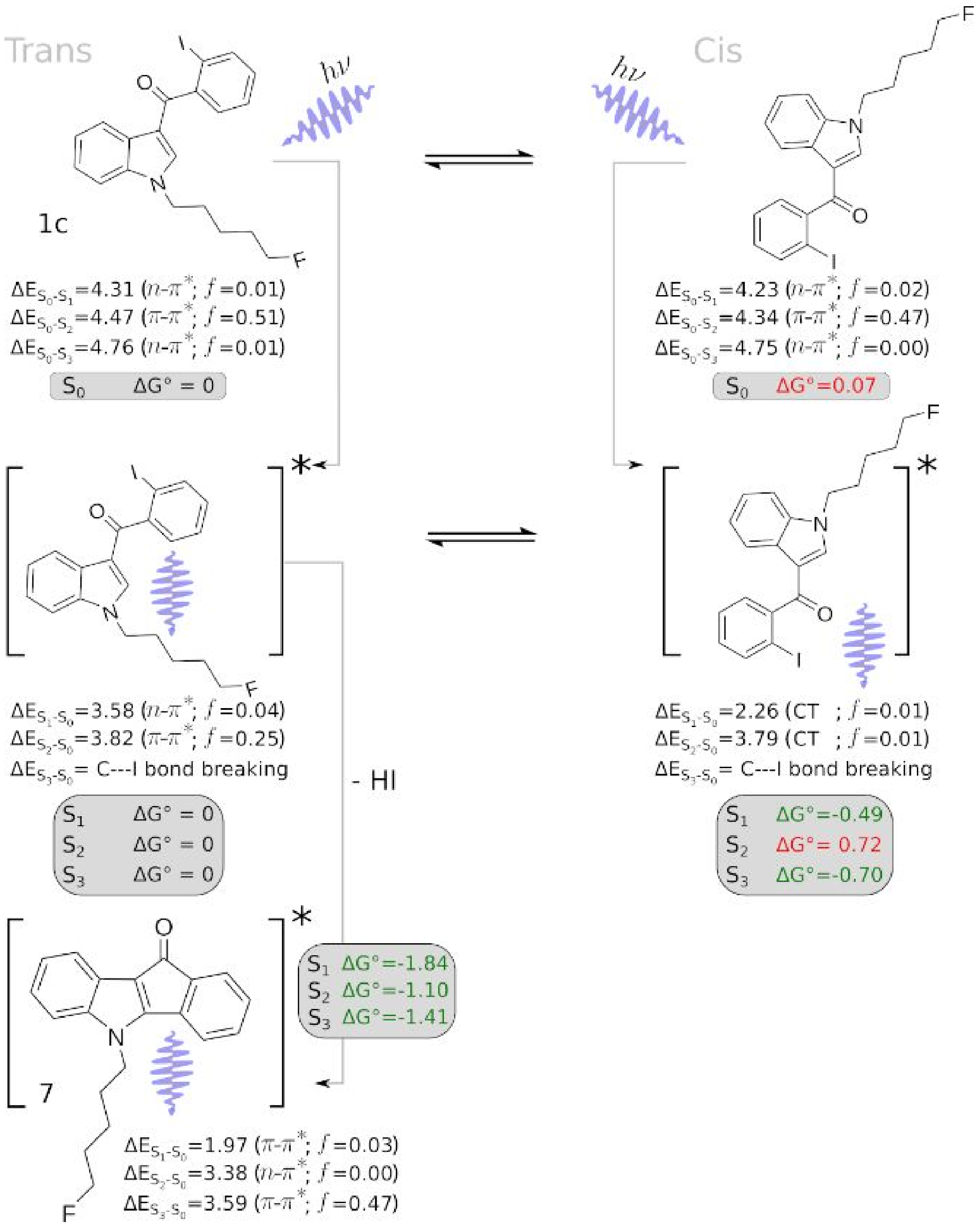

Scheme S1: Degradation processes for AM-694 in the ground and excited states. The absorption, emission energies and the nature of the corresponding electronic transitions are shown. The relative stabilities were calculated with respect to the most stable isomer. The ΔG values for each process connected with grey arrows are also displayed. The asterisk symbolises electronically excited species.

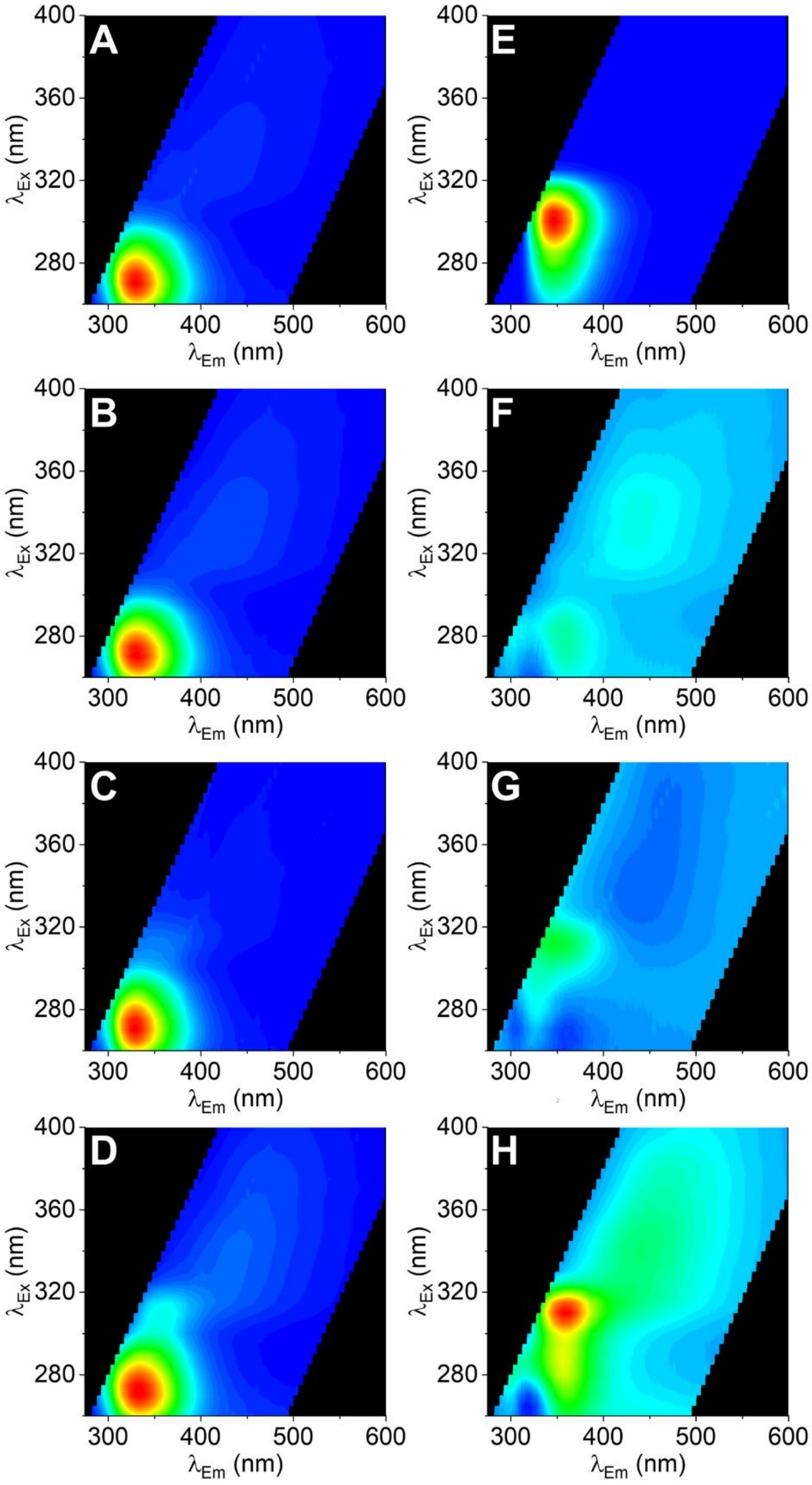
A
B
C
D
E
F
G
H
λ_{Ex} (nm)
λ_{Em} (nm)
400
360
320
280
300
400
500
600

Figure S4. FSFs for (A) average of saliva/methanol samples used, (B-D) 10, 50 and 250 ng/mL MDMB-4en-PINACA in saliva/methanol and (E) 250 ng/mL MDMB-4en-PINACA in methanol only. Conditions: 1 mL cuvette, 20 °C, 1:2 v/v saliva: methanol. Red colouration represents emission with a relative intensity of one, and blue represents an intensity of zero. (F-H) Difference heat maps of 10, 50 and 250 ng/mL MDMB-4en-PINACA in saliva minus averaged saliva, coloured from -0.05 to 0.228 relative intensity (blue to red).

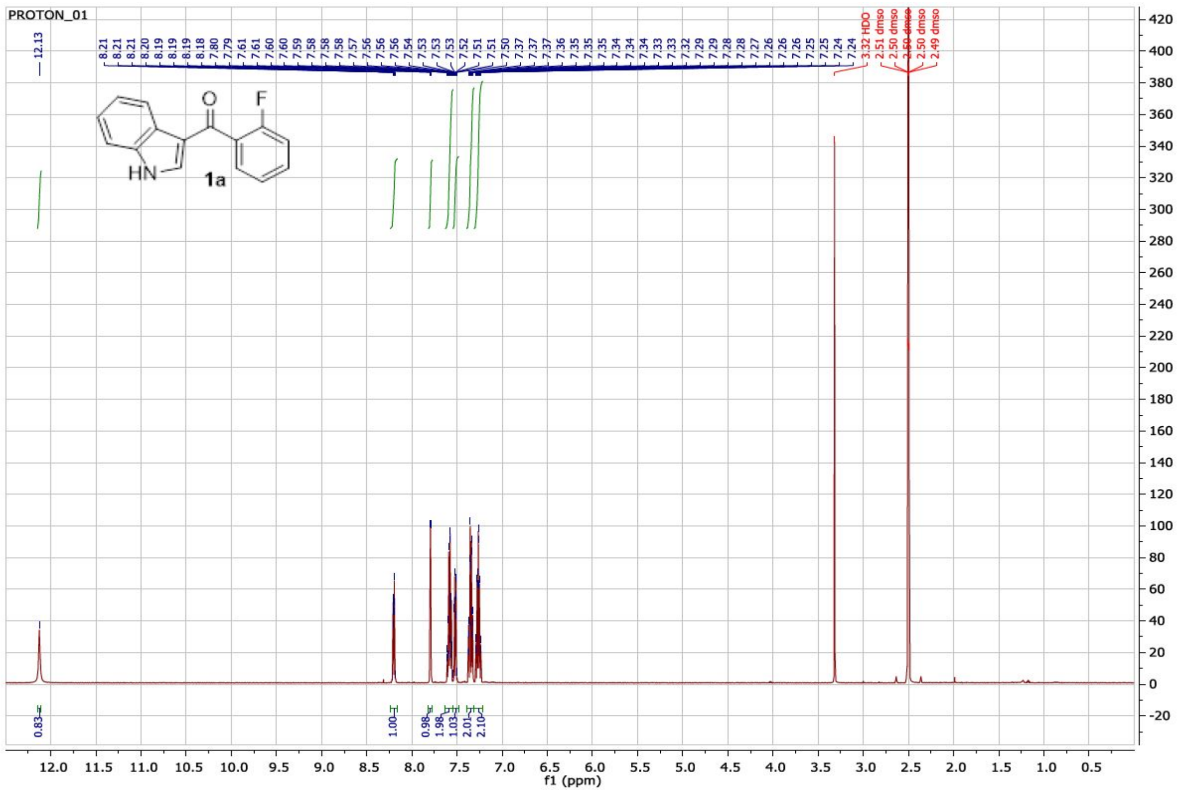

Figure S5: ^{1}H NMR spectrum for **1a**.

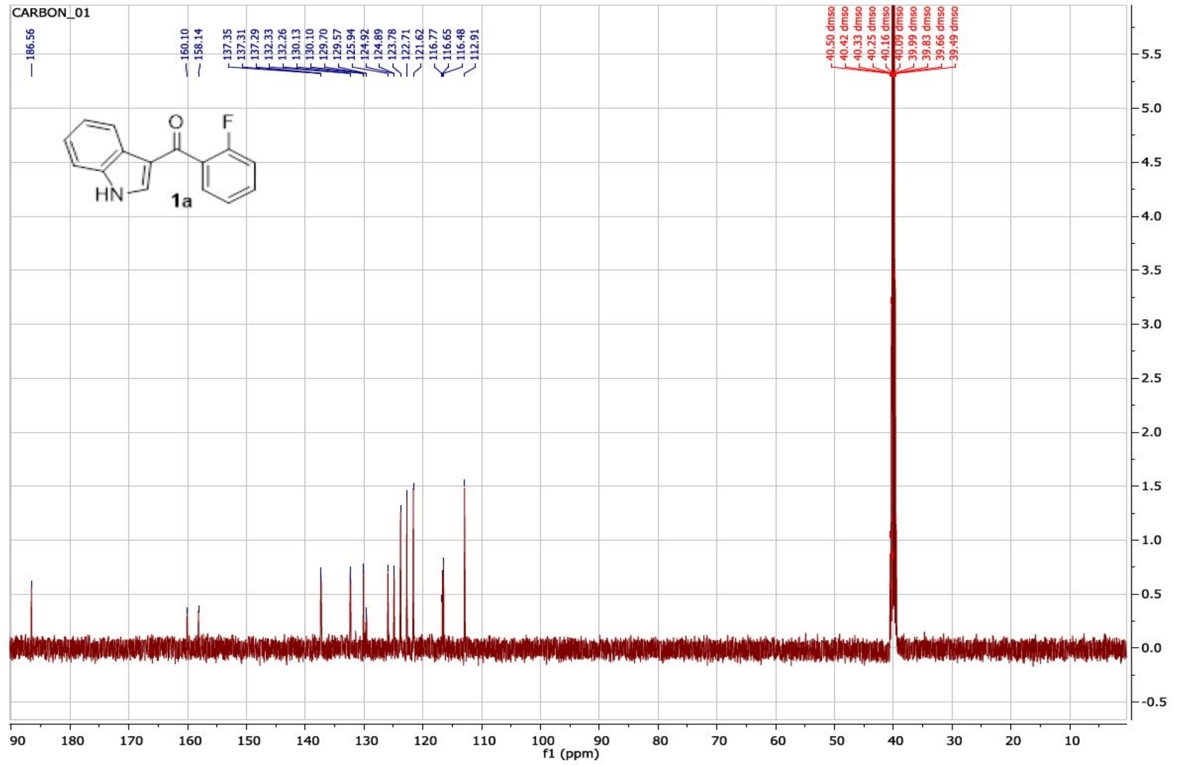

Figure S6: ^{13}C NMR spectrum for **1a**.

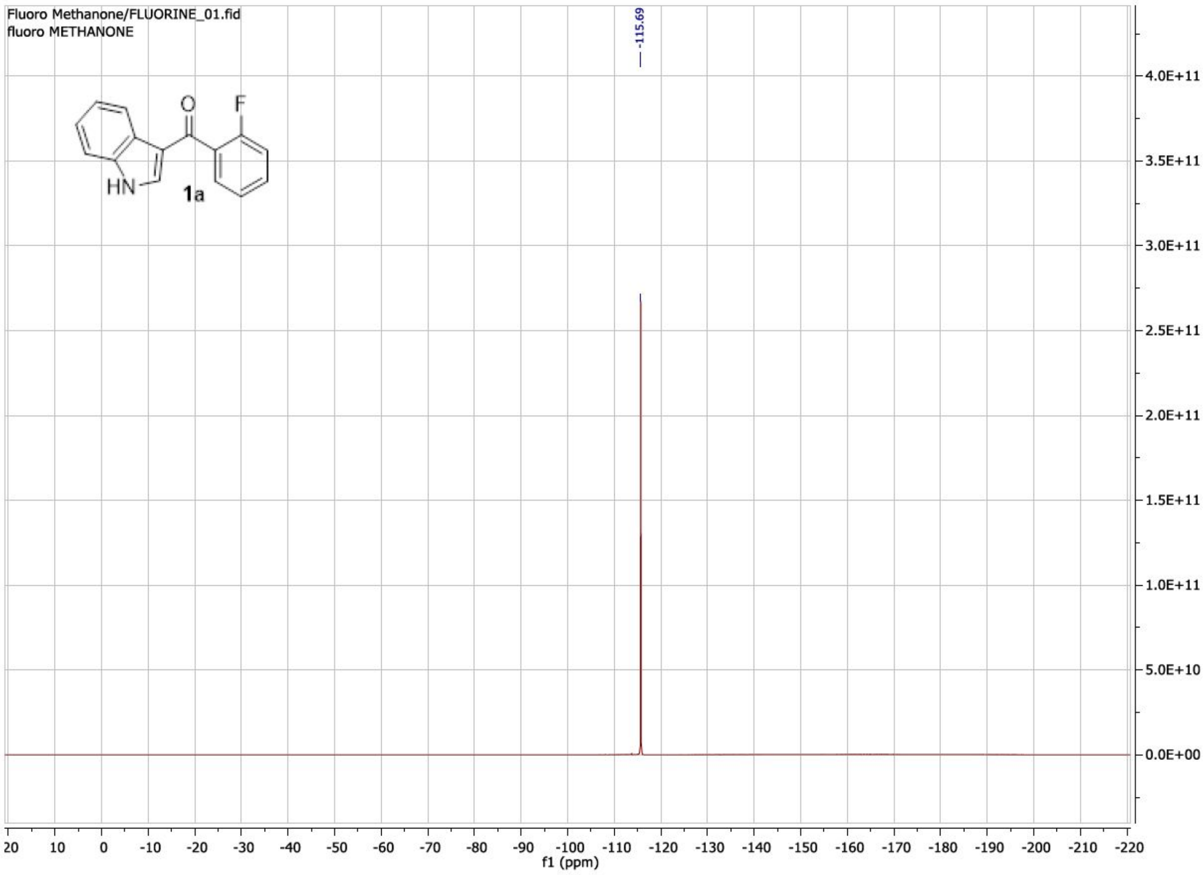

Figure S7: ^{19}F NMR spectrum for **1a**.

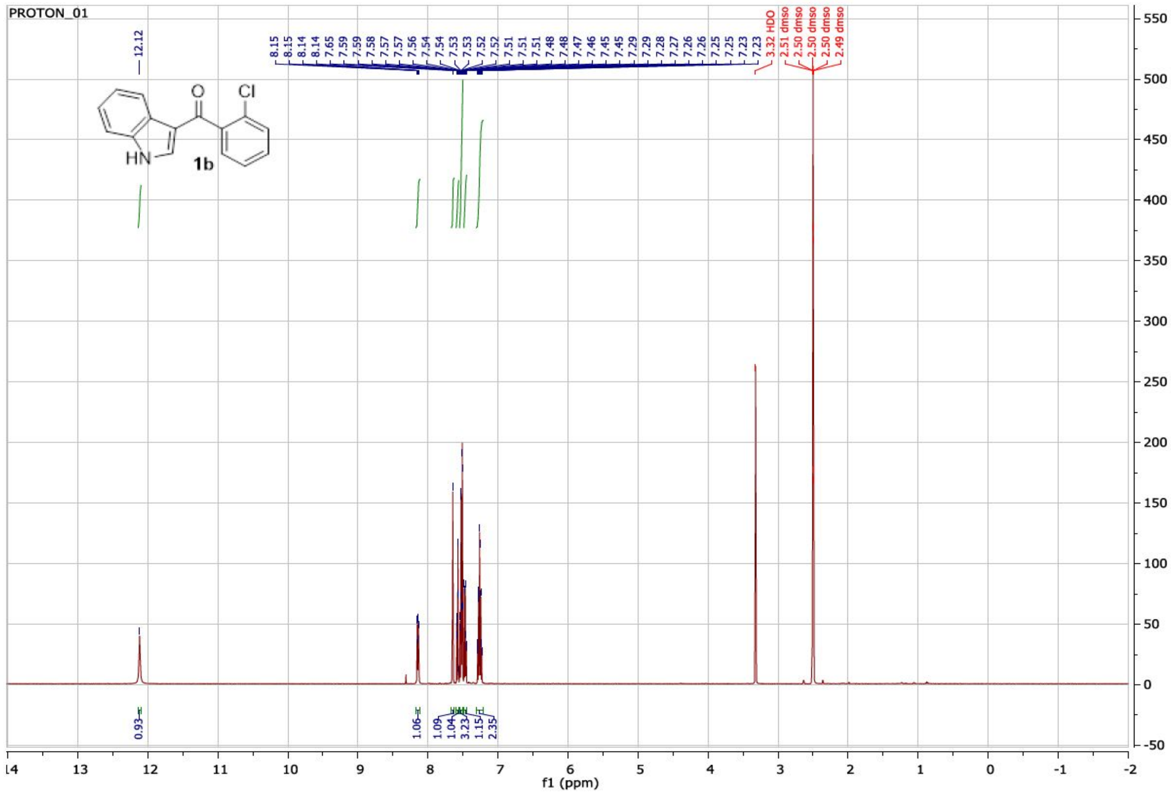

Figure S8: ^{1}H NMR spectrum for **1b**.

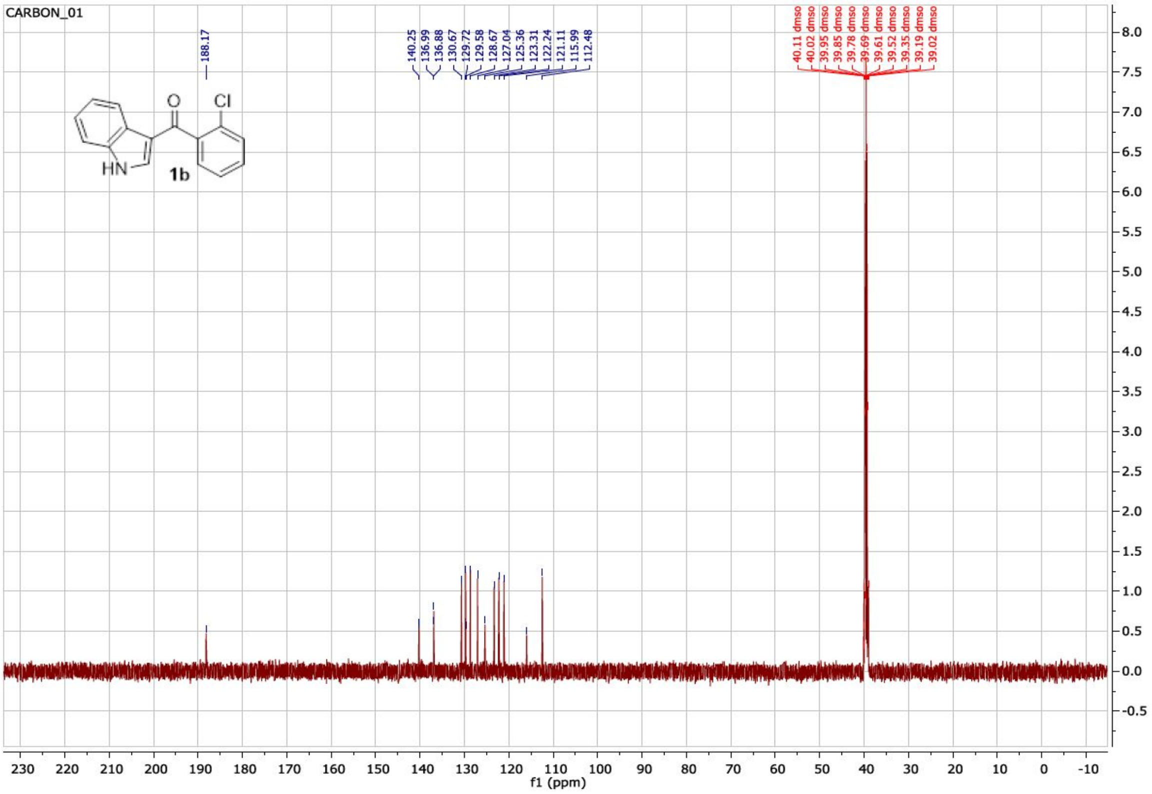

Figure S9: ^{13}C NMR spectrum for **1b**.

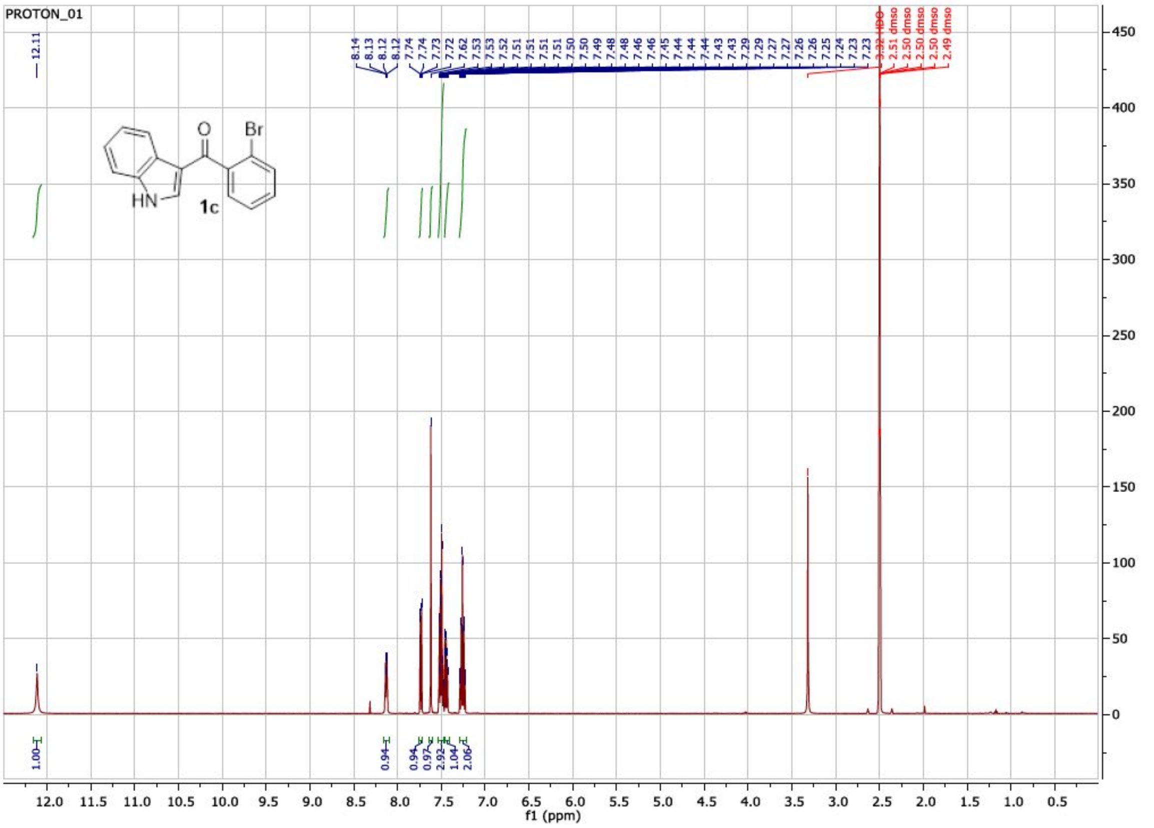

Figure S10: ^{1}H NMR spectrum for **1c**.

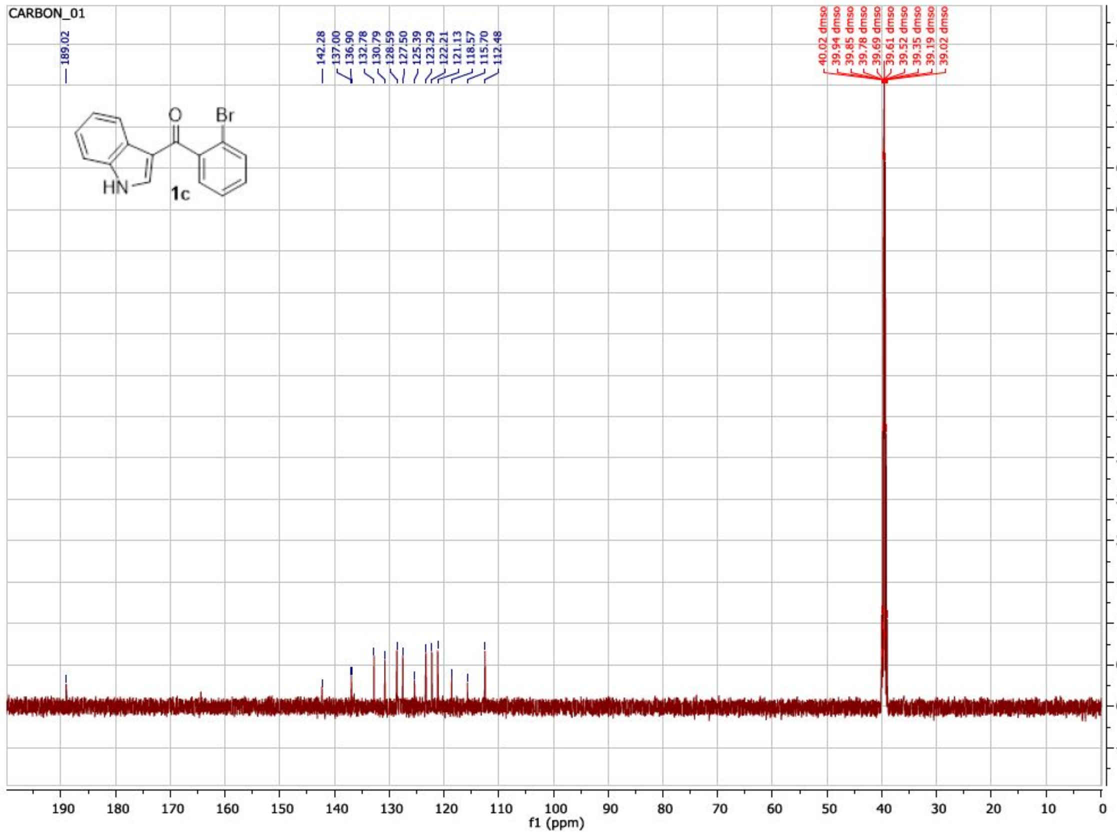

Figure S11: ^{13}C NMR spectrum for **1c**.

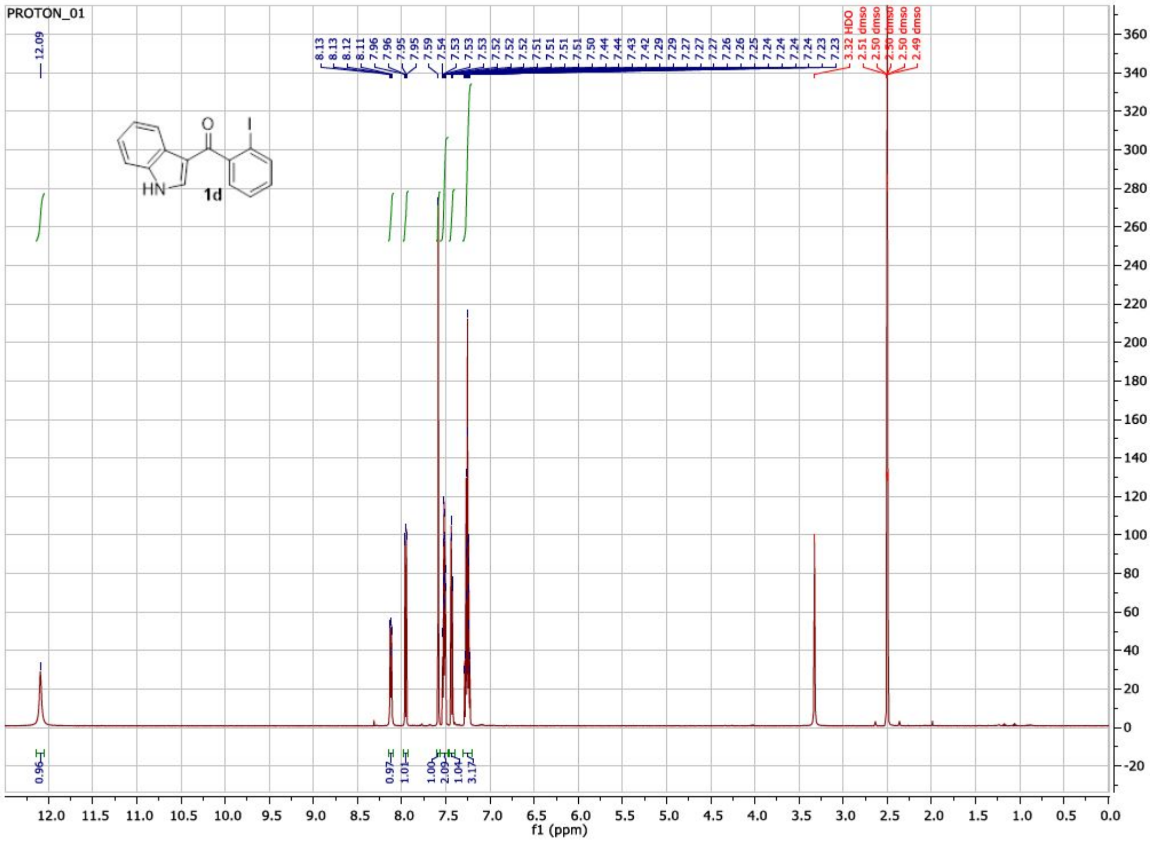

Figure S12: ^{1}H NMR spectrum for **1d**.

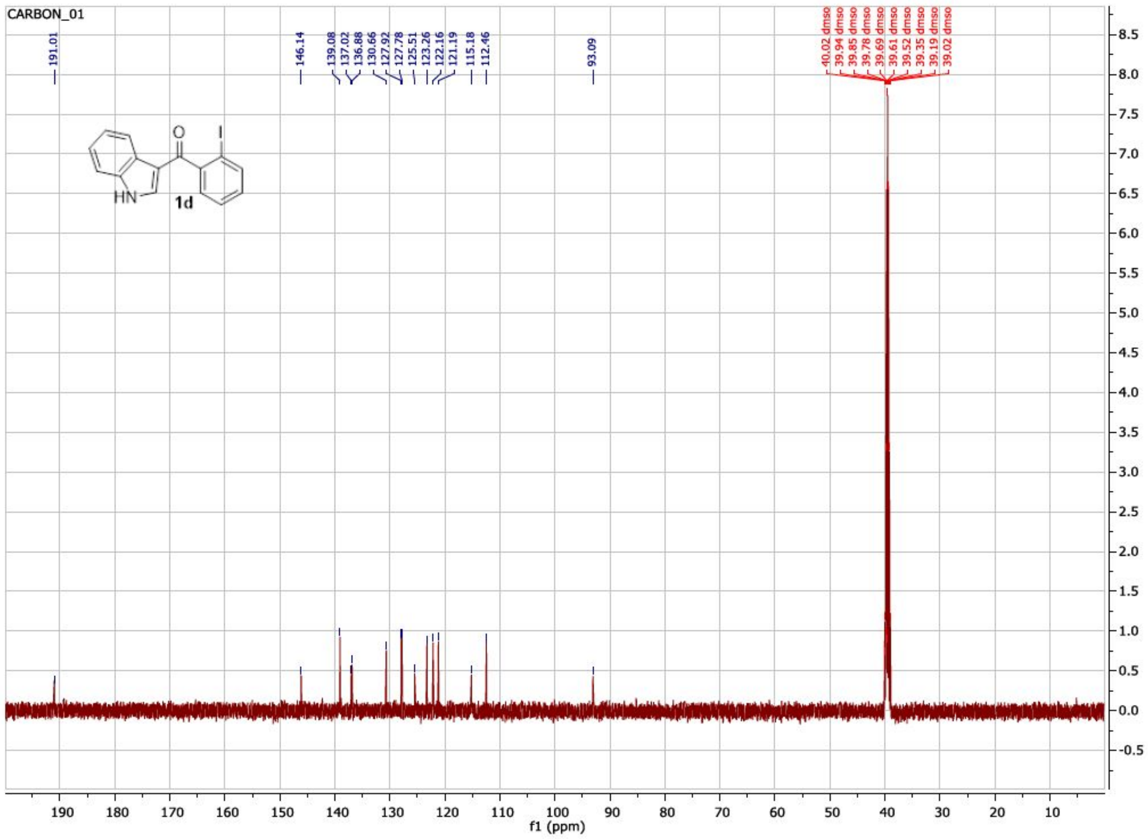

Figure S13: ^{13}C NMR spectrum for **1d**.

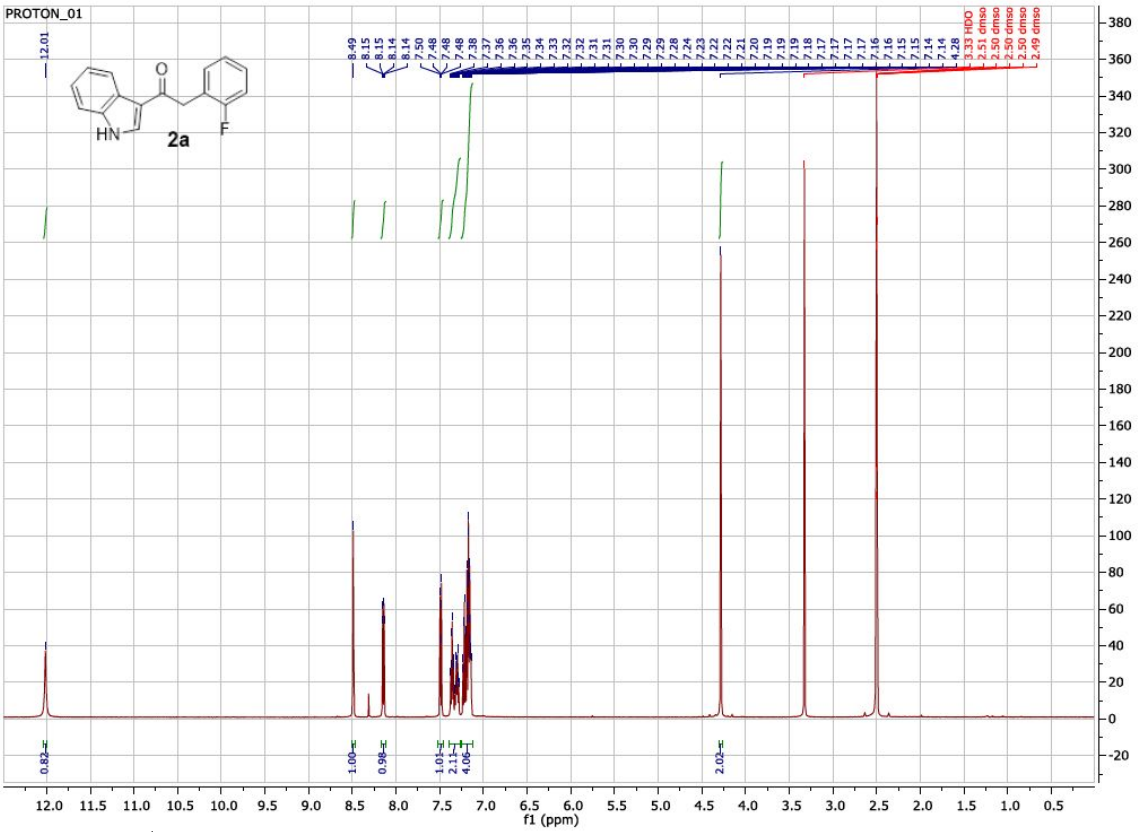

Figure S14: ^{1}H NMR spectrum for **2a**.

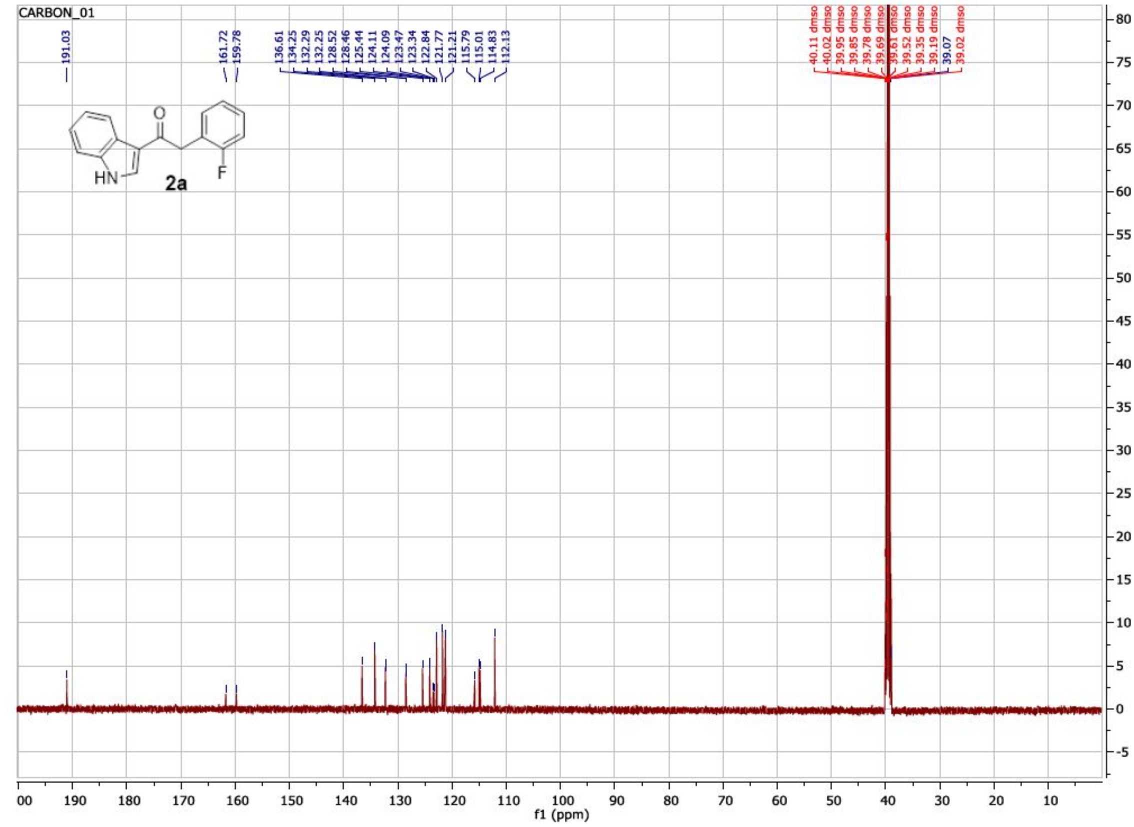

Figure S15: ^{13}C NMR spectrum for **2a**.

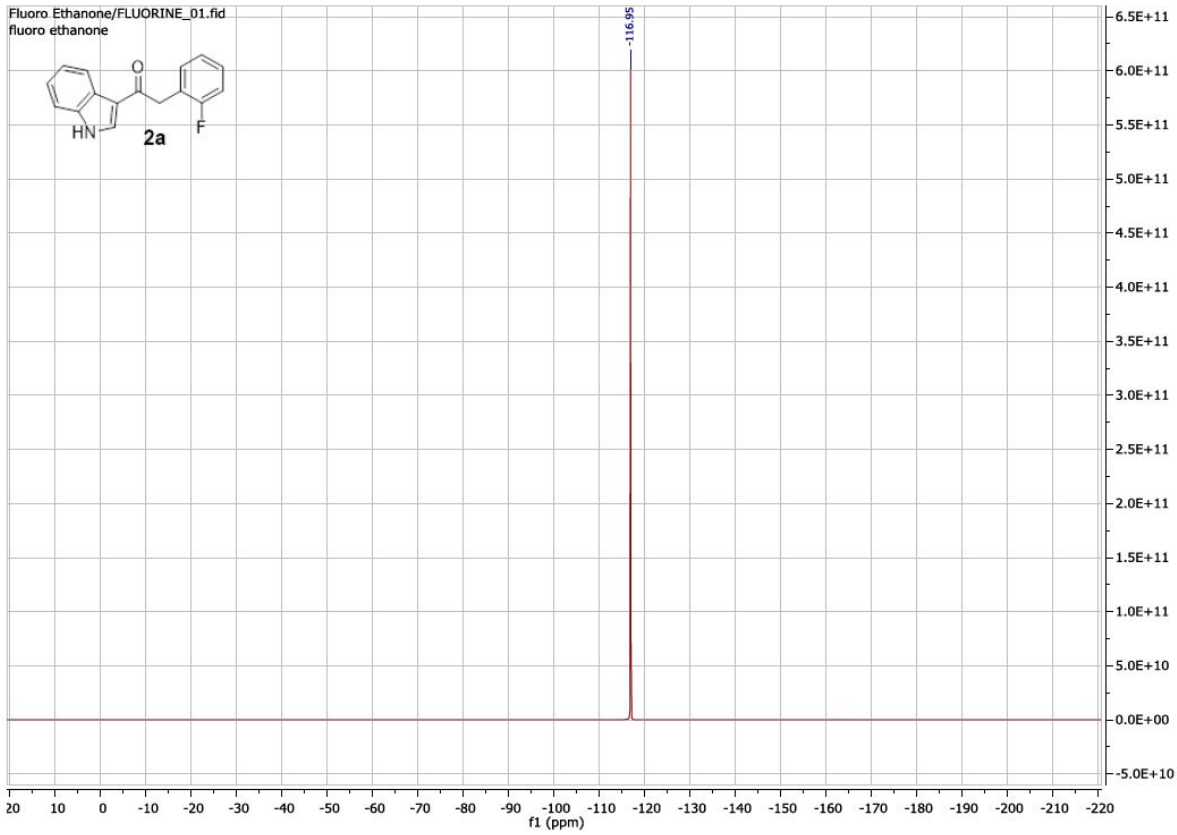

Figure S16: ^{19}F NMR spectrum for **2a**.

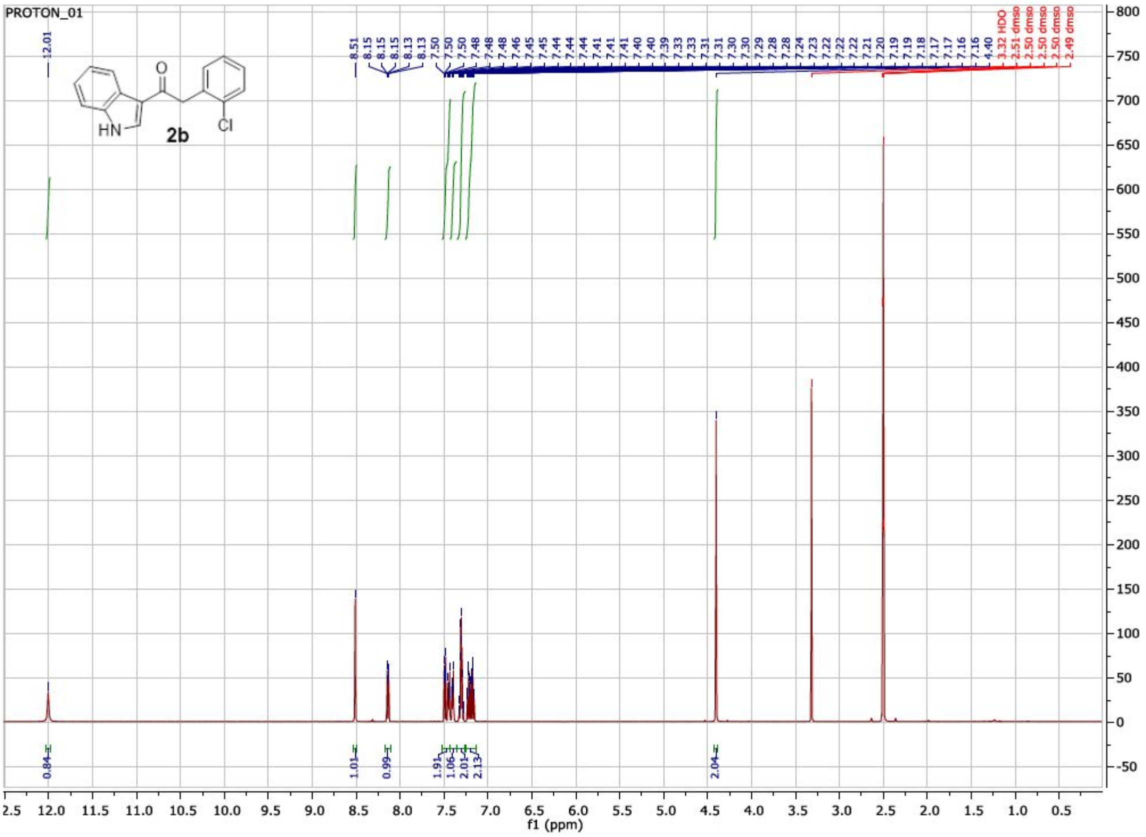

Figure S17: ^{1}H NMR spectrum for **2b**.

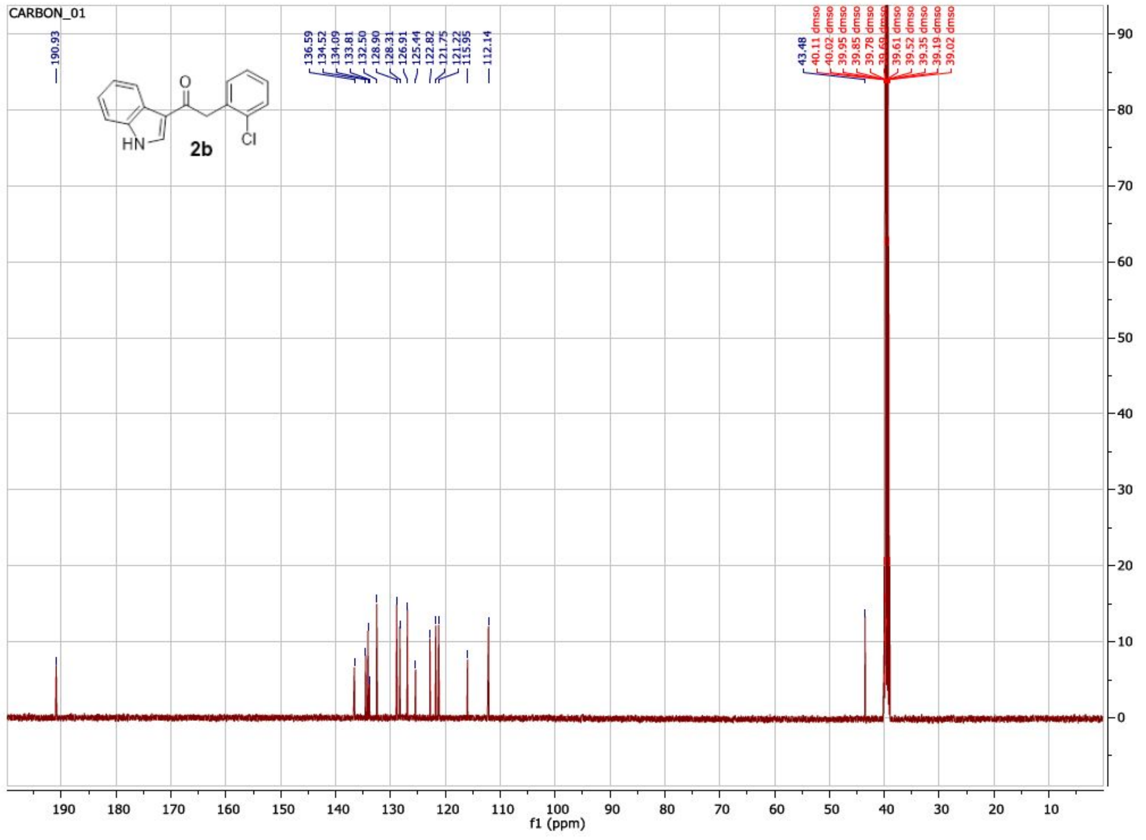

Figure S18: ^{13}C NMR spectrum for **2b**.

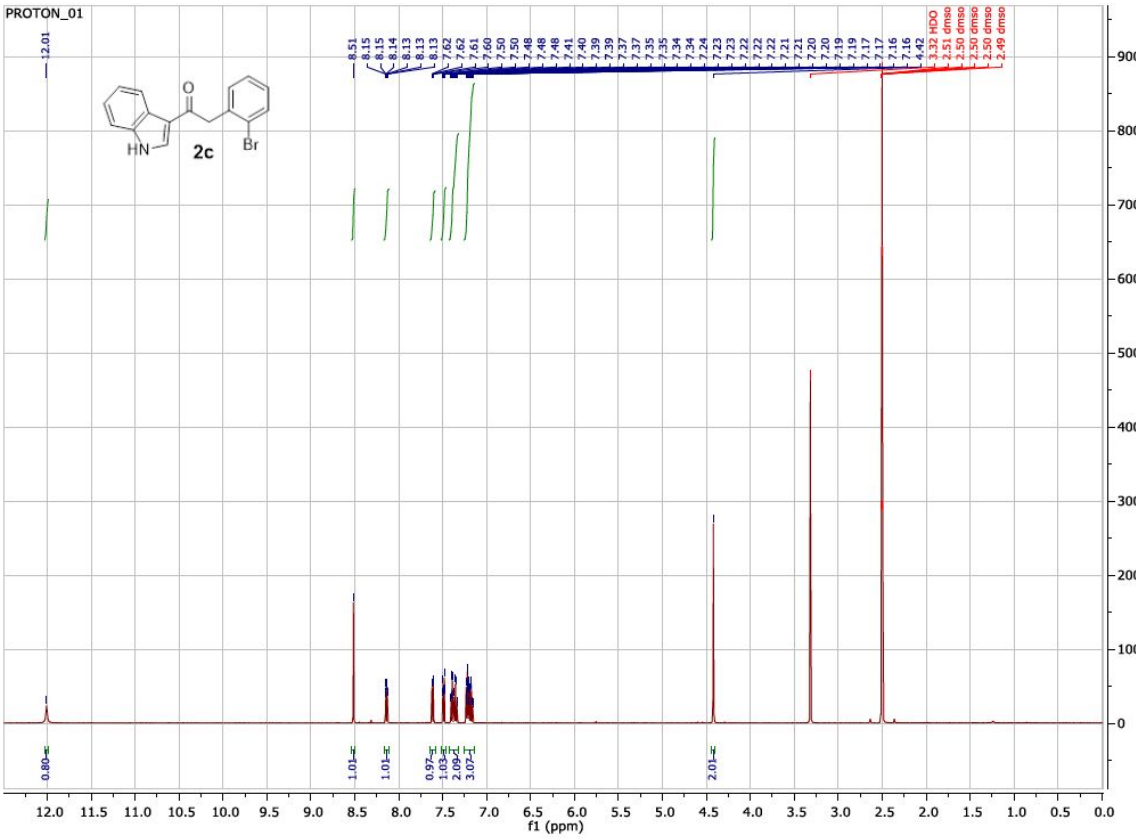

Figure S19: ^{1}H NMR spectrum for **2c**.

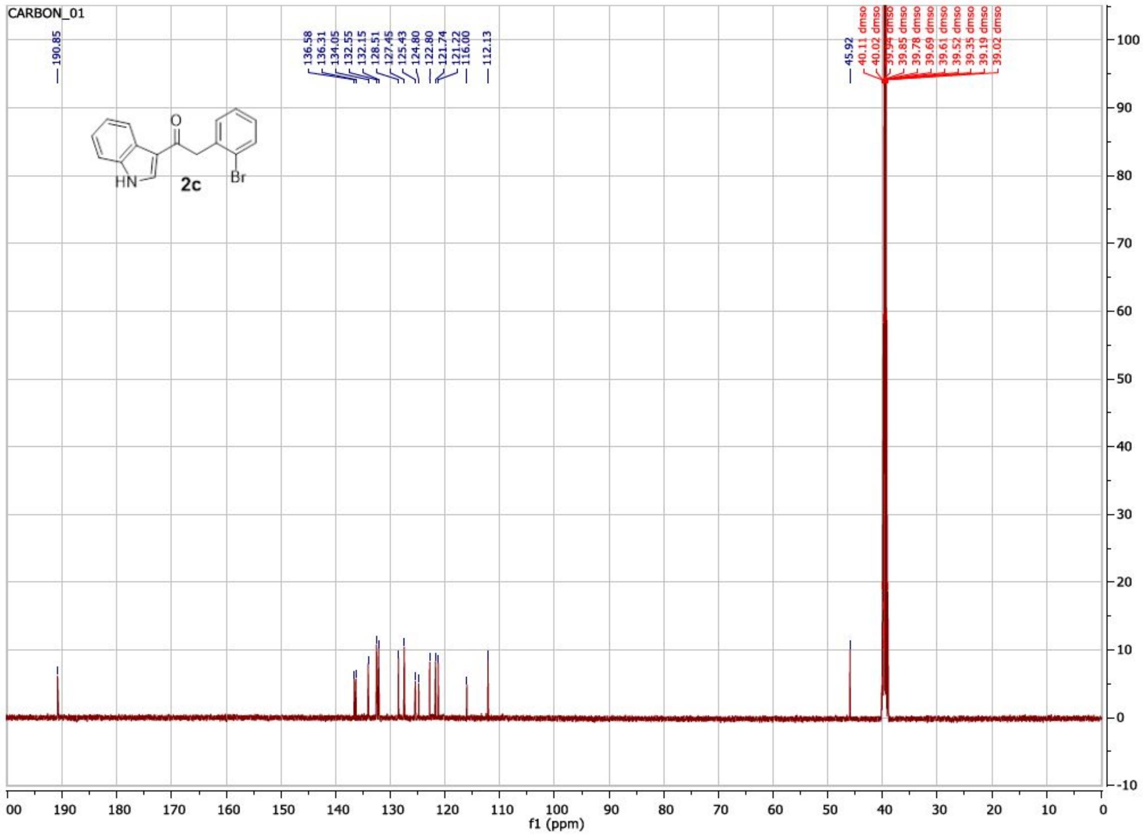

Figure S20: ^{13}C NMR spectrum for **2c**.

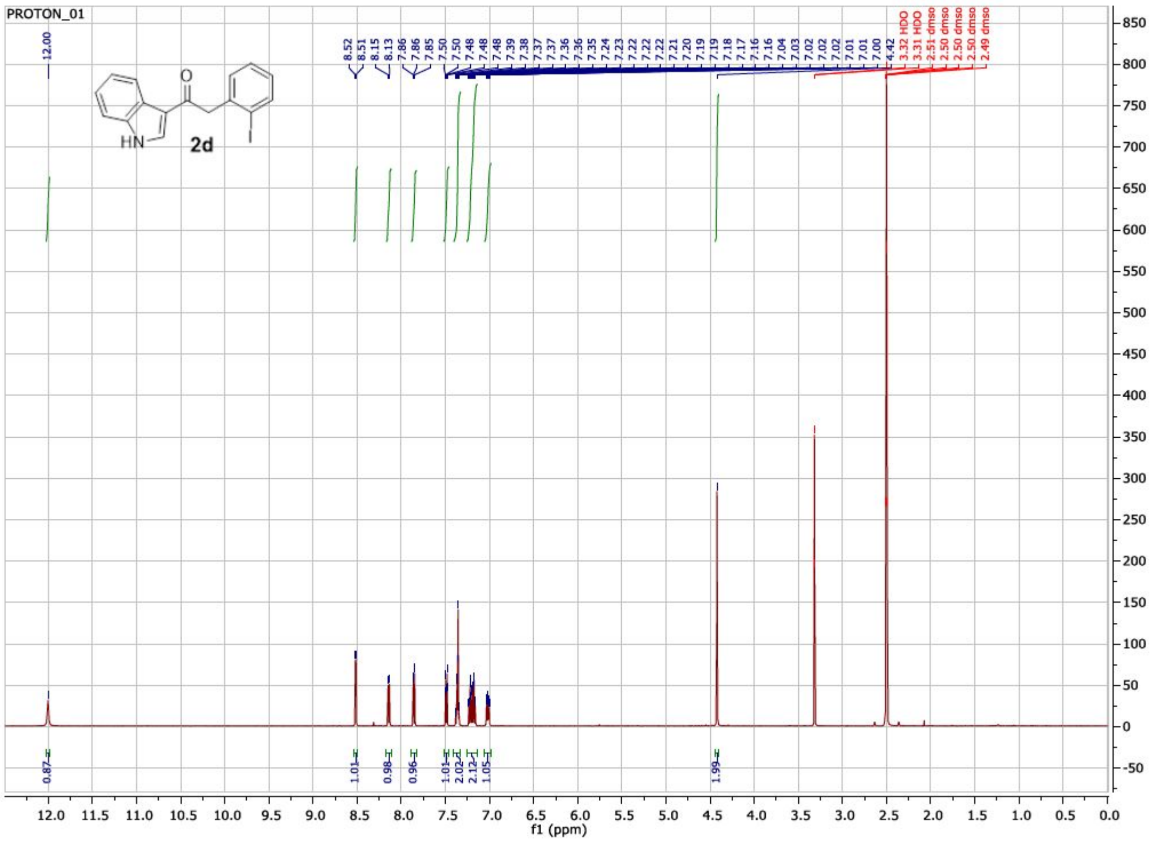

Figure S21: ^{1}H NMR spectrum for **2d**.

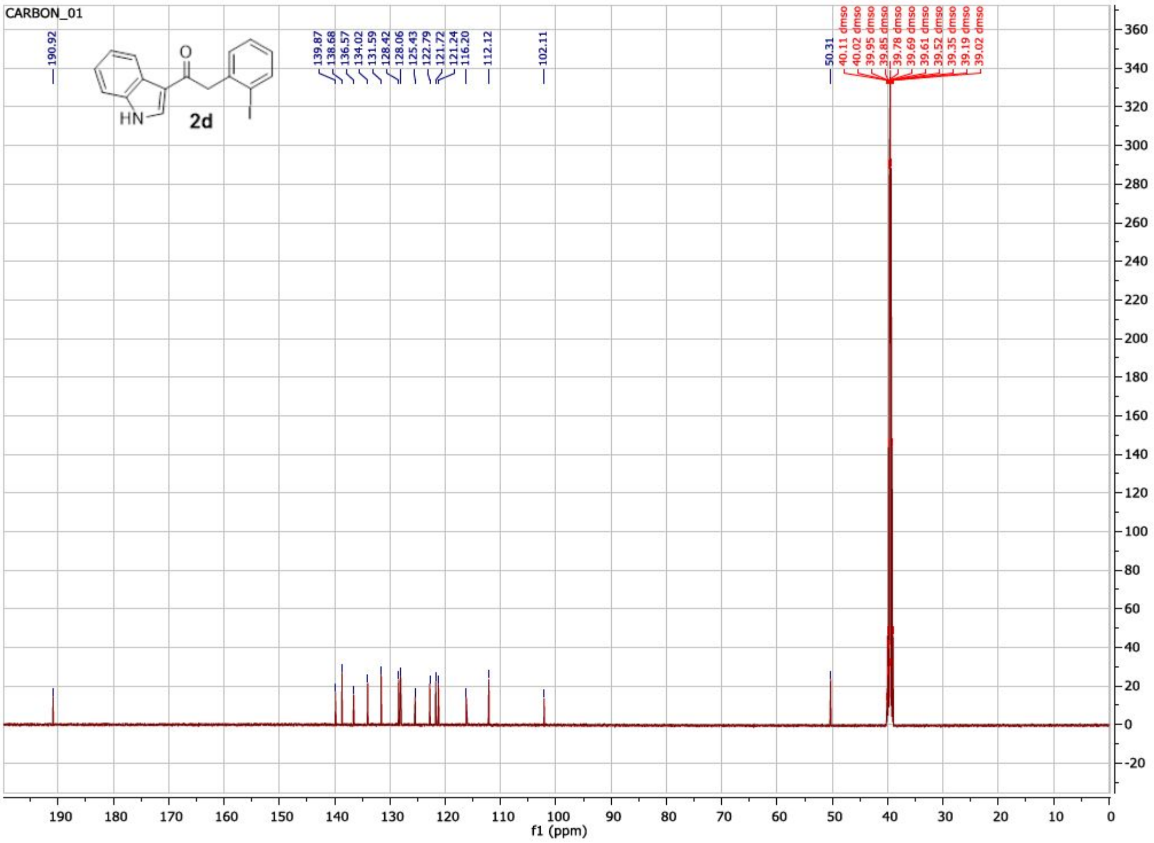

Figure S22: ^{13}C NMR spectrum for **2d**.

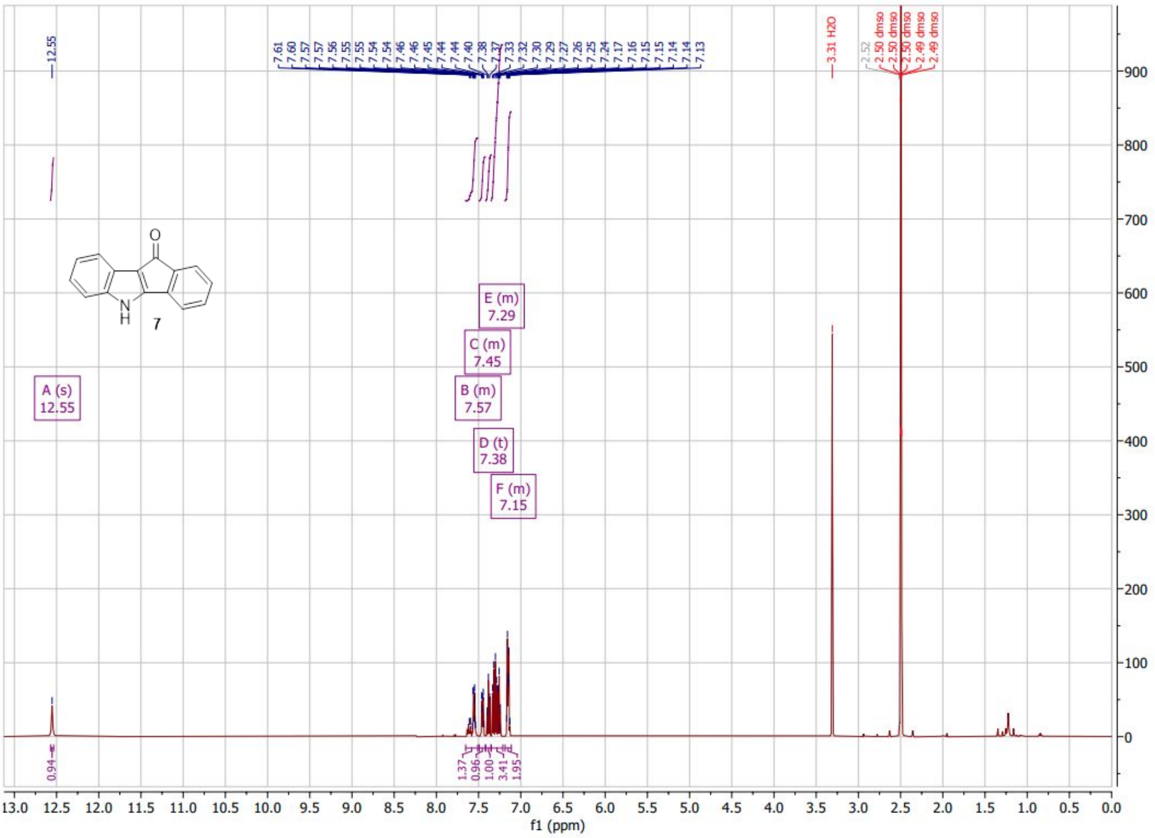

Figure S23: ^{1}H NMR spectrum for **7**.

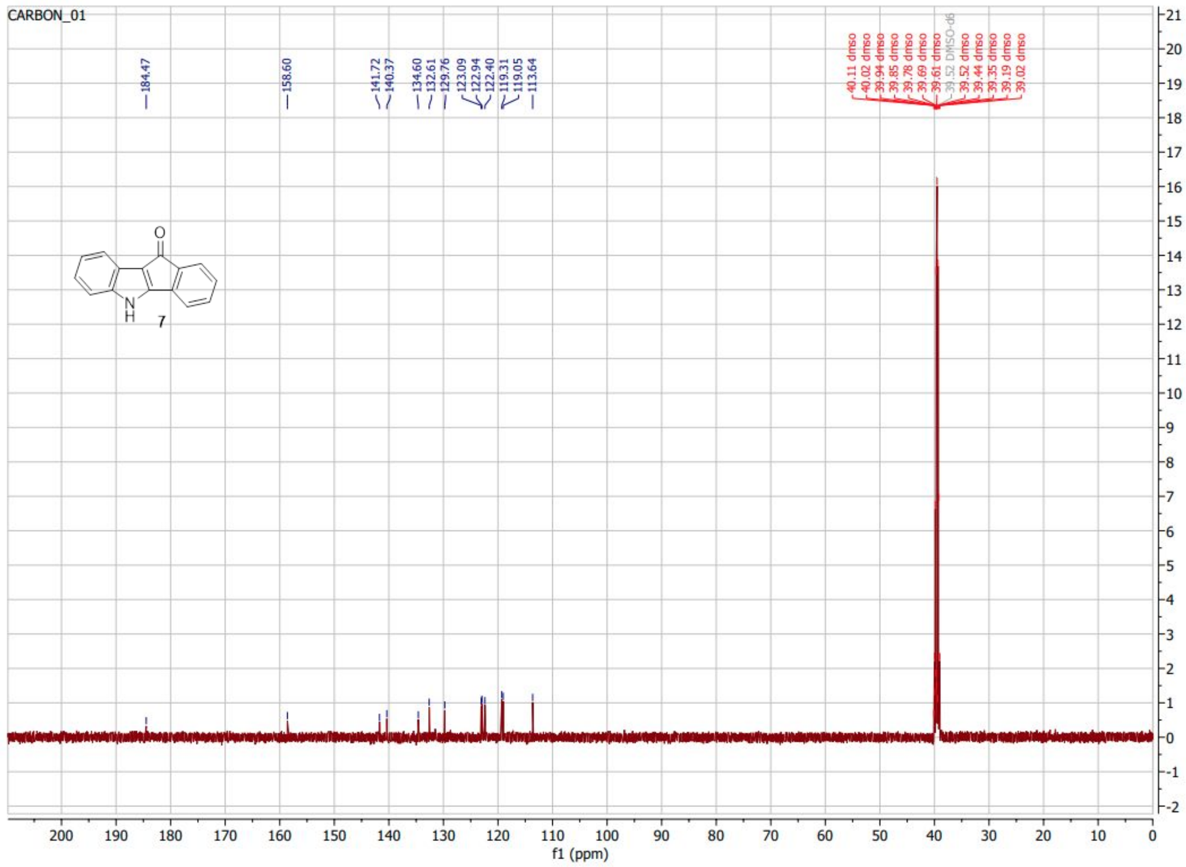

Figure S24: ^{13}C NMR spectrum for **7**.

HRMS Analysis of Compounds **1a-d, 2a-d, 7:**

Compound Table

Compound Label	RT (min)	Observed mass (m/z)	Neutral observed mass (Da)	Theoretical mass (Da)	Mass error (ppm)	Isotope match score (%)
Cpd 1: C15 H10 F N O	0.73	238.0668	239.0740	239.0746	-2.75	98.28

Mass errors of between -5.00 and 5.00 ppm with isotope match scores above 60% are considered confirmation of molecular formulae

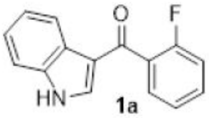

1a

Compound Table

Compound Label	RT (min)	Observed mass (m/z)	Neutral observed mass (Da)	Theoretical mass (Da)	Mass error (ppm)	Isotope match score (%)
Cpd 1: C15 H10 Cl N O	0.72	254.0374	255.0446	255.0451	-2.05	99.01

Mass errors of between -5.00 and 5.00 ppm with isotope match scores above 60% are considered confirmation of molecular formulae

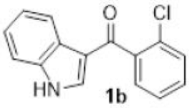

1b

Compound Table

Compound Label	RT (min)	Observed mass (m/z)	Neutral observed mass (Da)	Theoretical mass (Da)	Mass error (ppm)	Isotope match score (%)
Cpd 1: C15 H10 Br N O	0.69	297.9866	298.9938	298.9946	-2.65	99.62

Mass errors of between -5.00 and 5.00 ppm with isotope match scores above 60% are considered confirmation of molecular formulae

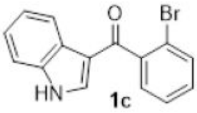

1c

Compound Table

Compound Label	RT (min)	Observed mass (m/z)	Neutral observed mass (Da)	Theoretical mass (Da)	Mass error (ppm)	Isotope match score (%)
Cpd 1: C15 H10 I N O	0.69	345.9724	346.9796	346.9807	-3.28	95.69

Mass errors of between -5.00 and 5.00 ppm with isotope match scores above 60% are considered confirmation of molecular formulae

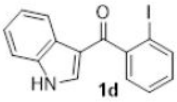

1d

Compound Table

Compound Label	RT (min)	Observed mass (m/z)	Neutral observed mass (Da)	Theoretical mass (Da)	Mass error (ppm)	Isotope match score (%)
Cpd 1: C16 H12 F N O	0.71	252.0829	253.0902	253.0903	-0.56	98.56

Mass errors of between -5.00 and 5.00 ppm with isotope match scores above 60% are considered confirmation of molecular formulae

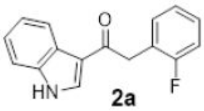

2a

Compound Table

Compound Label	RT (min)	Observed mass (m/z)	Neutral observed mass (Da)	Theoretical mass (Da)	Mass error (ppm)	Isotope match score (%)
Cpd 1: C16 H12 Cl N O	0.70	268.0536	269.0607	269.0607	-0.04	97.80

Mass errors of between -5.00 and 5.00 ppm with isotope match scores above 60% are considered confirmation of molecular formulae

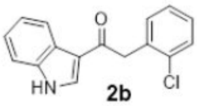

2b

Compound Table

Compound Label	RT (min)	Observed mass (m/z)	Neutral observed mass (Da)	Theoretical mass (Da)	Mass error (ppm)	Isotope match score (%)
Cpd 1: C16 H12 Br N O	0.68	312.0028	313.0101	313.0102	-0.56	98.80

Mass errors of between -5.00 and 5.00 ppm with isotope match scores above 60% are considered confirmation of molecular formulae

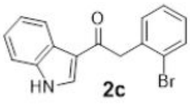

2c

Compound Table

Compound Label	RT (min)	Observed mass (m/z)	Neutral observed mass (Da)	Theoretical mass (Da)	Mass error (ppm)	Isotope match score (%)
Cpd 1: C16 H12 I N O	0.69	359.9891	360.9964	360.9964	0.05	98.43

Mass errors of between -5.00 and 5.00 ppm with isotope match scores above 60% are considered confirmation of molecular formulae

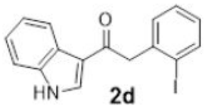

2d

Compound Table

Compound Label	RT (min)	Observed mass (m/z)	Neutral observed mass (Da)	Theoretical mass (Da)	Mass error (ppm)	Isotope match score (%)
Cpd 1: C15 H9 N O	0.74	218.0613	219.0686	219.0684	0.88	97.22

Mass errors of between -5.00 and 5.00 ppm with isotope match scores above 60% are considered confirmation of molecular formulae

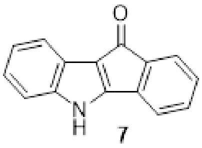

7

IR Confirmation of Compounds **1a-d, 2a-d, 7:**

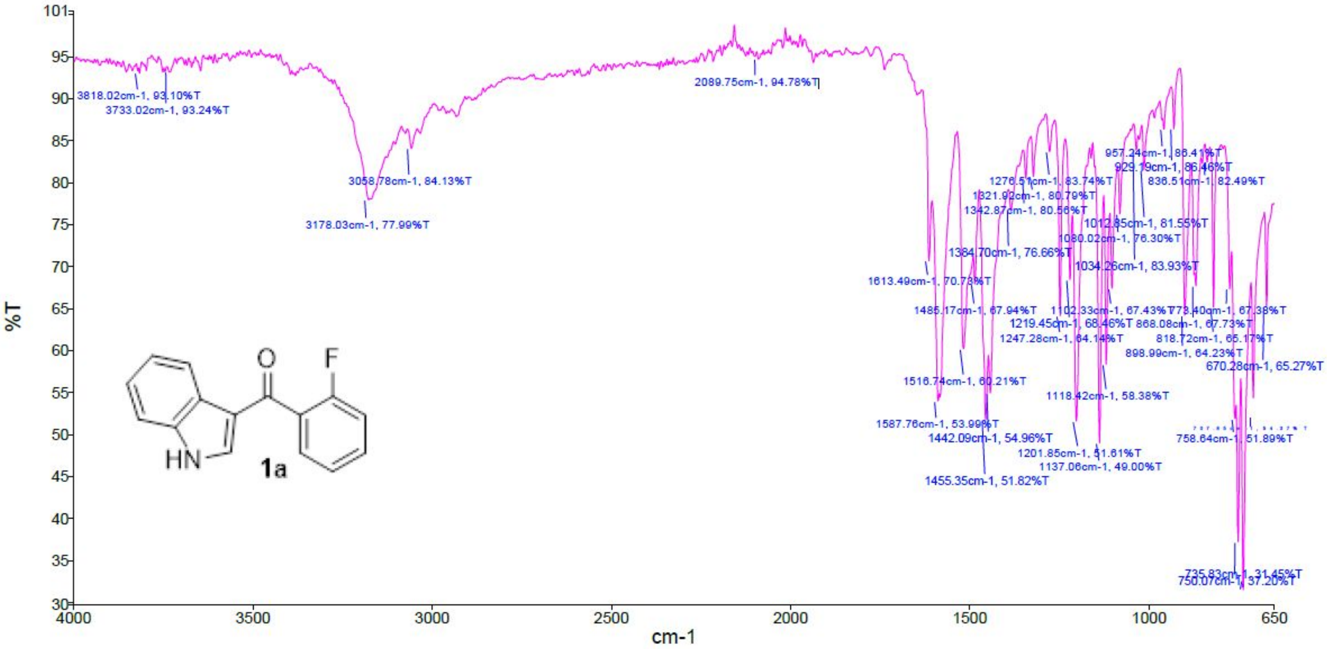
%T
cm-1
1a
HN
O
F
3818.02cm-1, 93.10%T
3733.02cm-1, 93.24%T
3058.78cm-1, 84.13%T
3178.03cm-1, 77.99%T
2089.75cm-1, 94.78%T
1613.49cm-1, 70.78%T
1485.17cm-1, 67.94%T
1516.74cm-1, 60.21%T
1587.76cm-1, 53.99%T
1442.09cm-1, 54.96%T
1455.35cm-1, 51.82%T
1276.51cm-1, 83.74%T
1321.92cm-1, 80.79%T
1342.87cm-1, 80.56%T
1384.70cm-1, 76.66%T
1219.45cm-1, 68.46%T
1247.28cm-1, 64.14%T
1201.85cm-1, 51.61%T
1137.06cm-1, 49.00%T
1118.42cm-1, 58.38%T
1102.33cm-1, 67.43%T
1080.02cm-1, 76.30%T
1034.26cm-1, 83.93%T
1012.85cm-1, 81.55%T
957.24cm-1, 86.41%T
929.79cm-1, 86.46%T
836.51cm-1, 82.49%T
773.40cm-1, 67.38%T
868.08cm-1, 67.73%T
818.72cm-1, 65.17%T
898.99cm-1, 64.23%T
670.28cm-1, 65.27%T
758.64cm-1, 51.89%T
735.83cm-1, 31.45%T
750.07cm-1, 37.20%T

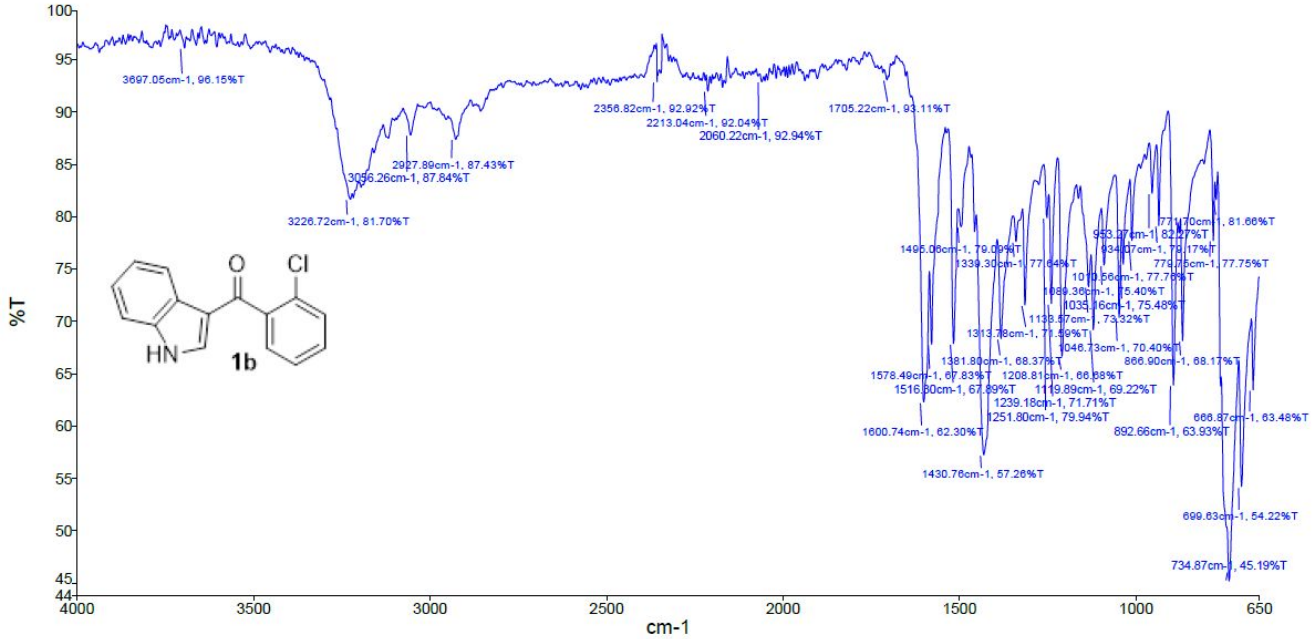
%T
cm-1
1b
HN
O
Cl
3697.05cm-1, 96.15%T
3226.72cm-1, 81.70%T
3056.26cm-1, 87.84%T
2927.89cm-1, 87.43%T
2356.82cm-1, 92.92%T
2213.04cm-1, 92.04%T
2060.22cm-1, 92.94%T
1705.22cm-1, 93.11%T
1495.06cm-1, 79.09%T
1339.30cm-1, 77.64%T
1313.78cm-1, 71.59%T
1381.80cm-1, 68.37%T
1578.49cm-1, 67.83%T
1516.80cm-1, 67.89%T
1600.74cm-1, 62.30%T
1430.76cm-1, 57.26%T
1208.81cm-1, 66.68%T
1239.18cm-1, 71.71%T
1251.80cm-1, 79.94%T
1119.89cm-1, 69.22%T
1138.57cm-1, 73.32%T
1089.36cm-1, 75.40%T
1035.16cm-1, 75.48%T
1046.73cm-1, 70.40%T
1010.56cm-1, 77.78%T
953.37cm-1, 82.27%T
934.07cm-1, 79.17%T
866.90cm-1, 68.17%T
892.66cm-1, 63.93%T
771.70cm-1, 81.66%T
779.75cm-1, 77.75%T
666.87cm-1, 63.48%T
699.63cm-1, 54.22%T
734.87cm-1, 45.19%T

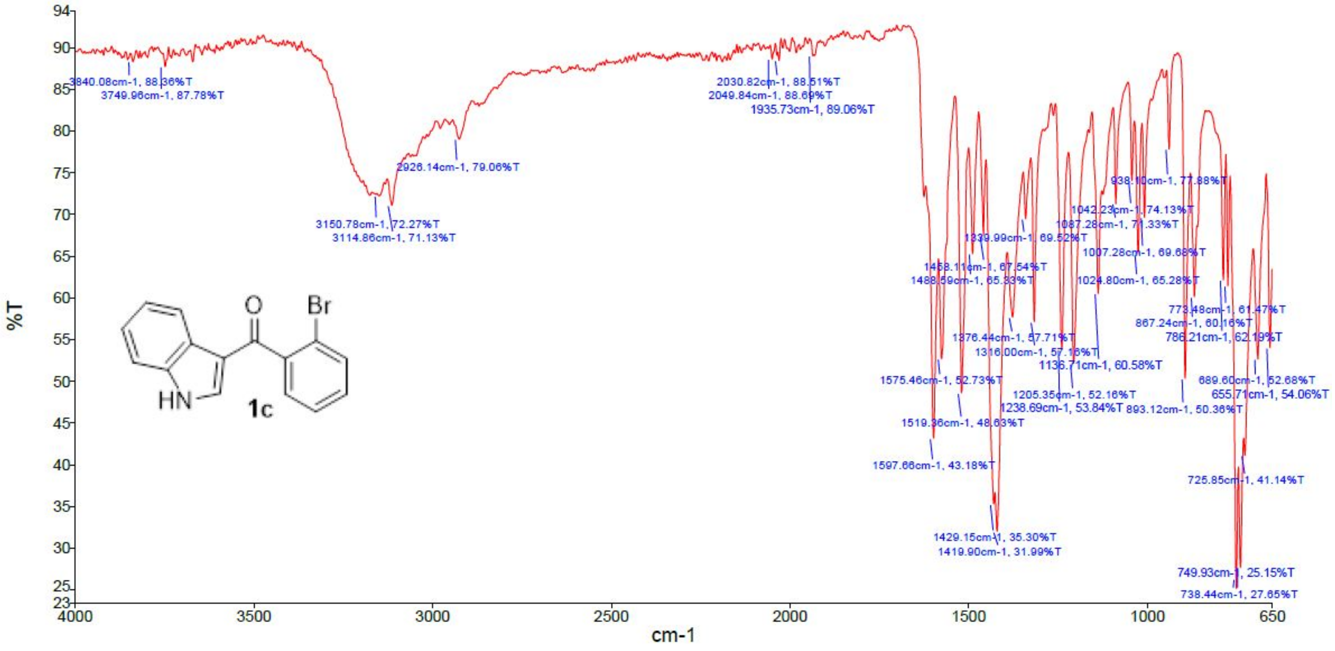
%T
cm-1
1c
3840.08cm-1, 88.36%T
3749.96cm-1, 87.78%T
3150.78cm-1, 72.27%T
3114.86cm-1, 71.13%T
2926.14cm-1, 79.06%T
2030.82cm-1, 88.51%T
2049.84cm-1, 88.69%T
1935.73cm-1, 89.06%T
1597.66cm-1, 43.18%T
1575.46cm-1, 52.73%T
1519.36cm-1, 48.63%T
1488.59cm-1, 65.33%T
1376.44cm-1, 57.71%T
1316.00cm-1, 57.16%T
1429.15cm-1, 35.30%T
1419.90cm-1, 31.99%T
1205.35cm-1, 52.16%T
1238.69cm-1, 53.84%T
1136.71cm-1, 60.58%T
1042.23cm-1, 74.13%T
1024.80cm-1, 65.28%T
1007.28cm-1, 69.68%T
893.12cm-1, 50.36%T
867.24cm-1, 60.16%T
786.21cm-1, 62.19%T
773.48cm-1, 61.47%T
689.60cm-1, 52.68%T
655.71cm-1, 54.06%T
725.85cm-1, 41.14%T
749.93cm-1, 25.15%T
738.44cm-1, 27.65%T

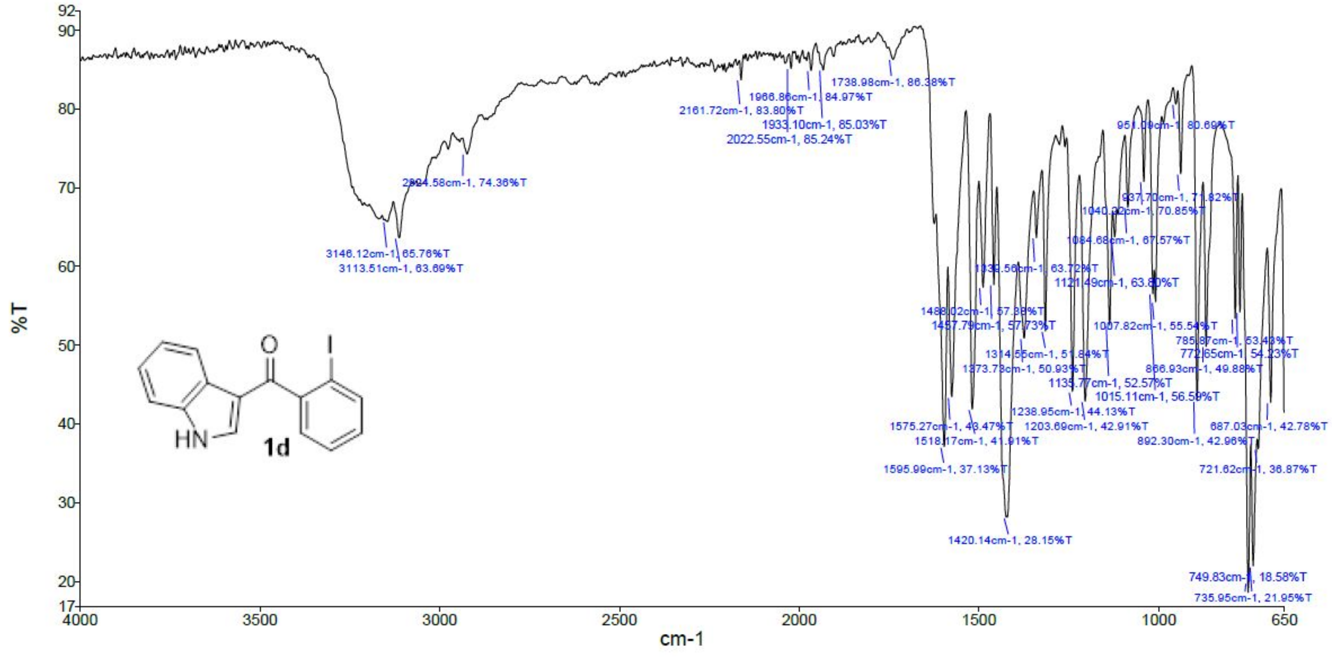
%T
cm-1
1d
3146.12cm-1, 65.76%T
3113.51cm-1, 63.69%T
2924.58cm-1, 74.36%T
2161.72cm-1, 83.80%T
1966.86cm-1, 84.97%T
1933.10cm-1, 85.03%T
2022.55cm-1, 85.24%T
1738.98cm-1, 86.38%T
1595.99cm-1, 37.13%T
1575.27cm-1, 48.47%T
1518.17cm-1, 41.91%T
1420.14cm-1, 28.15%T
1238.95cm-1, 44.13%T
1203.69cm-1, 42.91%T
1135.77cm-1, 52.57%T
1015.11cm-1, 56.59%T
1121.49cm-1, 63.80%T
1057.82cm-1, 55.54%T
951.09cm-1, 80.69%T
937.70cm-1, 71.82%T
866.93cm-1, 49.88%T
892.30cm-1, 42.96%T
772.65cm-1, 54.23%T
687.03cm-1, 42.78%T
721.62cm-1, 36.87%T
749.83cm-1, 18.58%T
735.95cm-1, 21.95%T

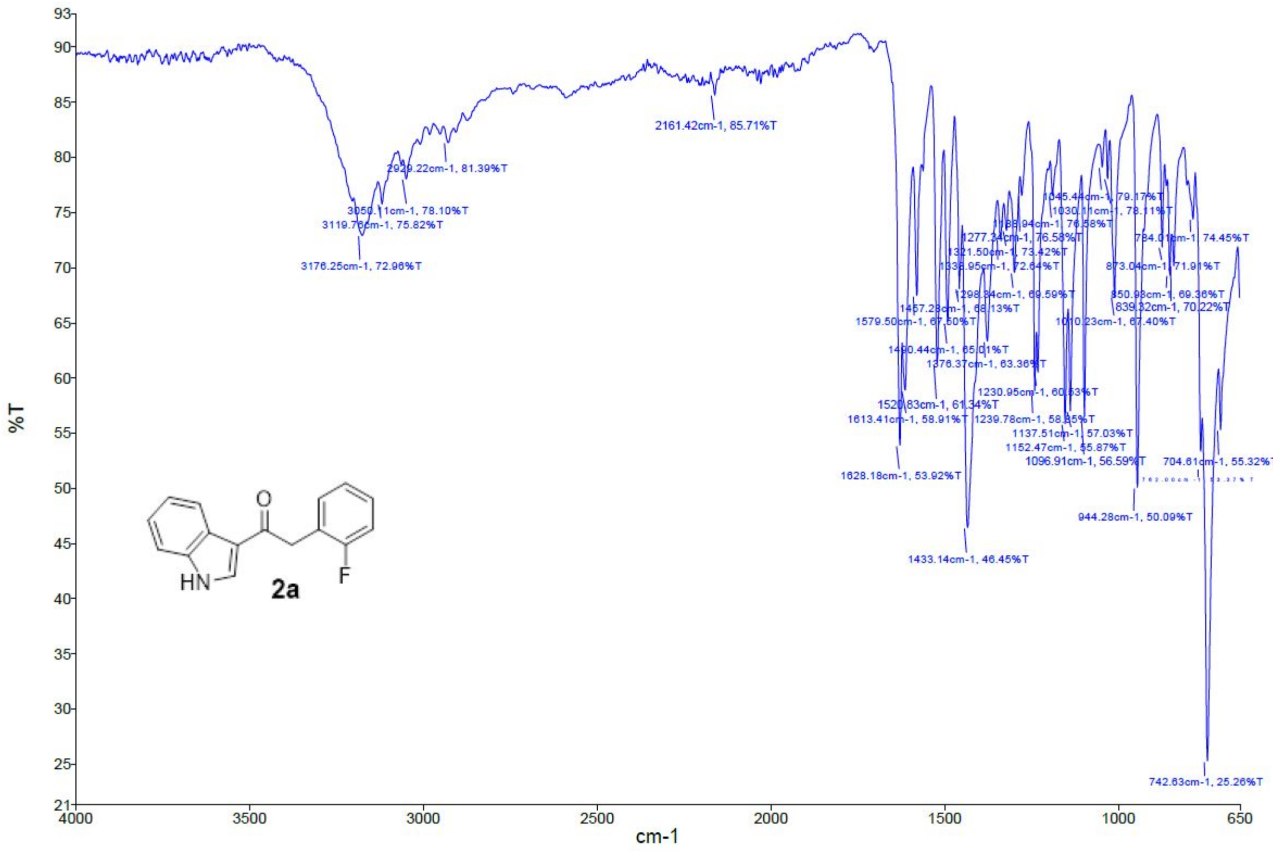

%T
cm-1
2a
HN
O
F
3176.25cm-1, 72.96%T
2161.42cm-1, 85.71%T
1433.14cm-1, 46.45%T
742.63cm-1, 25.26%T
944.28cm-1, 50.09%T
1628.18cm-1, 53.92%T
704.61cm-1, 55.32%T

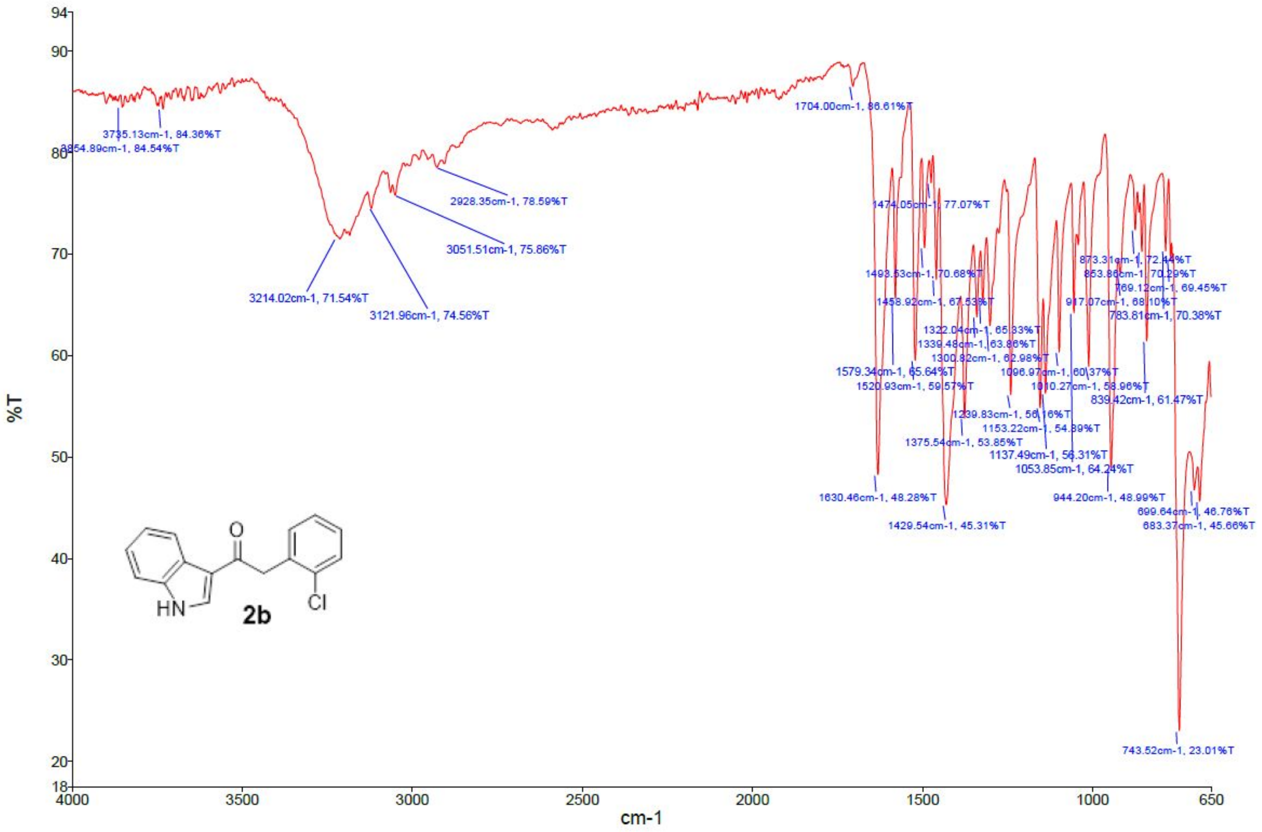
2b
%T
cm-1
1630.46cm-1, 48.28%T
1429.54cm-1, 45.31%T
743.52cm-1, 23.01%T
3214.02cm-1, 71.54%T
3121.96cm-1, 74.56%T
3051.51cm-1, 75.86%T
2928.35cm-1, 78.59%T
1704.00cm-1, 86.61%T

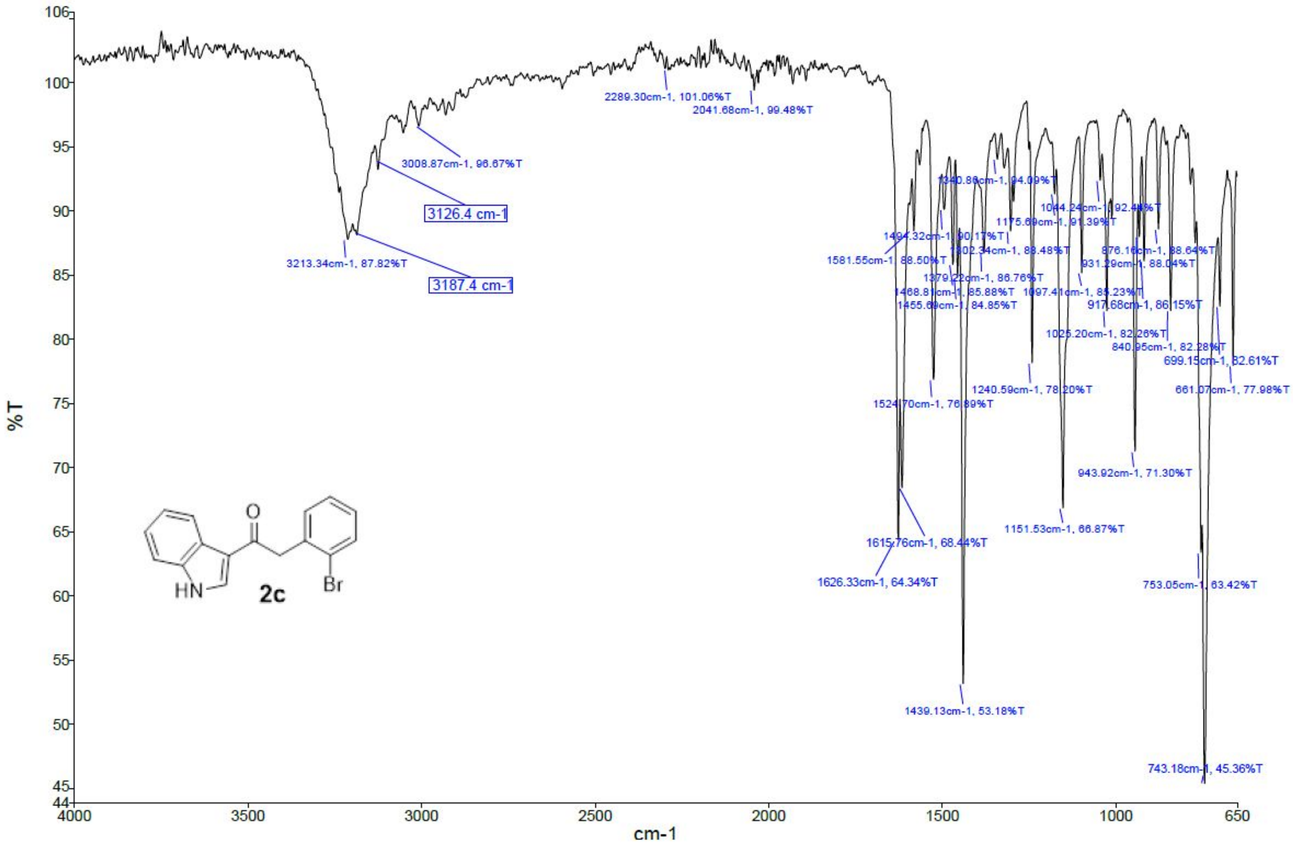
2c
%T
cm-1
3213.34cm-1, 87.82%T
3126.4 cm-1
3187.4 cm-1
3008.87cm-1, 96.67%T
2289.30cm-1, 101.06%T
2041.68cm-1, 99.48%T
1626.33cm-1, 64.34%T
1439.13cm-1, 53.18%T
1151.53cm-1, 66.87%T
943.92cm-1, 71.30%T
753.05cm-1, 63.42%T
743.18cm-1, 45.36%T

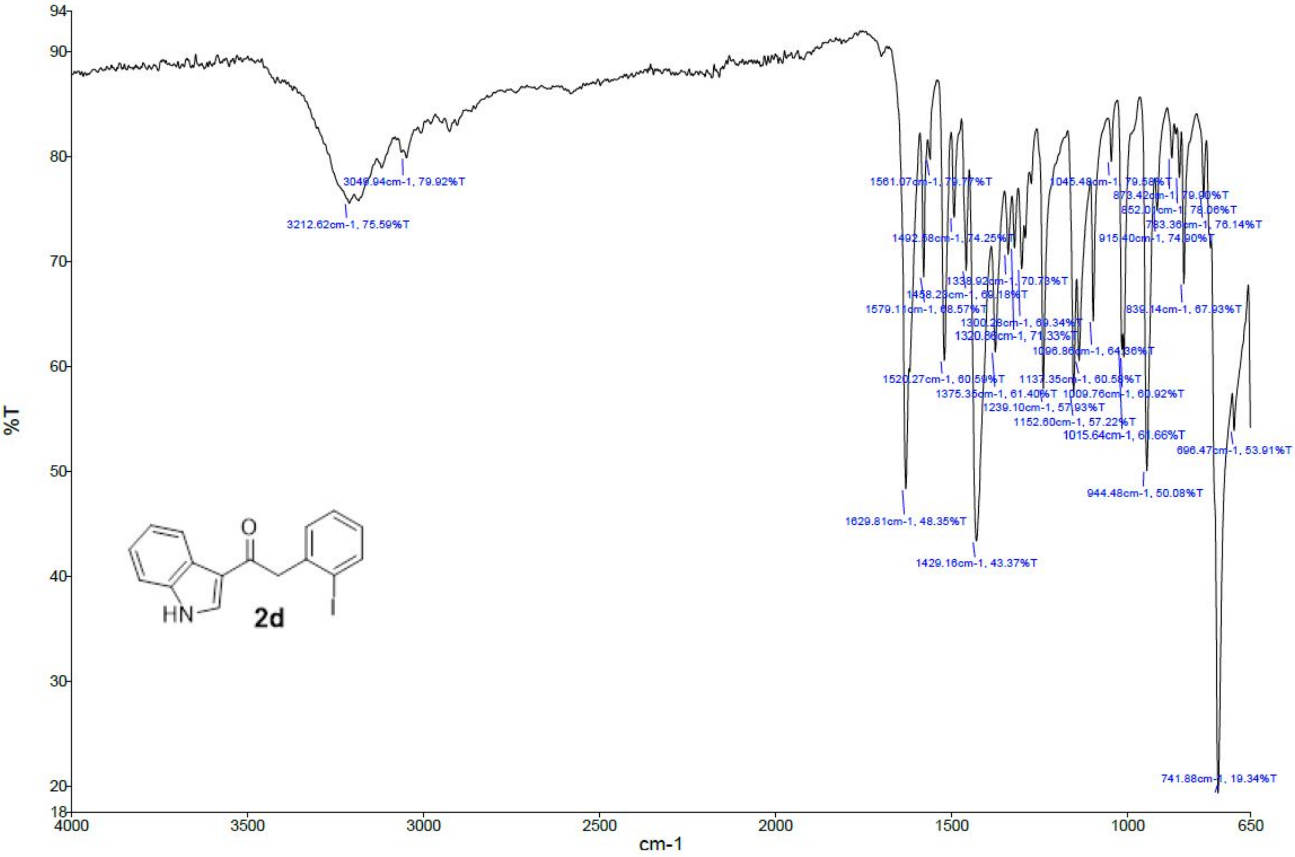
%T
cm-1
94
90
80
70
60
50
40
30
20
18
4000
3500
3000
2500
2000
1500
1000
650
3212.62cm-1, 75.59%T
1629.81cm-1, 48.35%T
1429.16cm-1, 43.37%T
1520.27cm-1, 60.59%T
1239.10cm-1, 57.93%T
1152.60cm-1, 57.22%T
944.48cm-1, 50.08%T
741.88cm-1, 19.34%T
2d

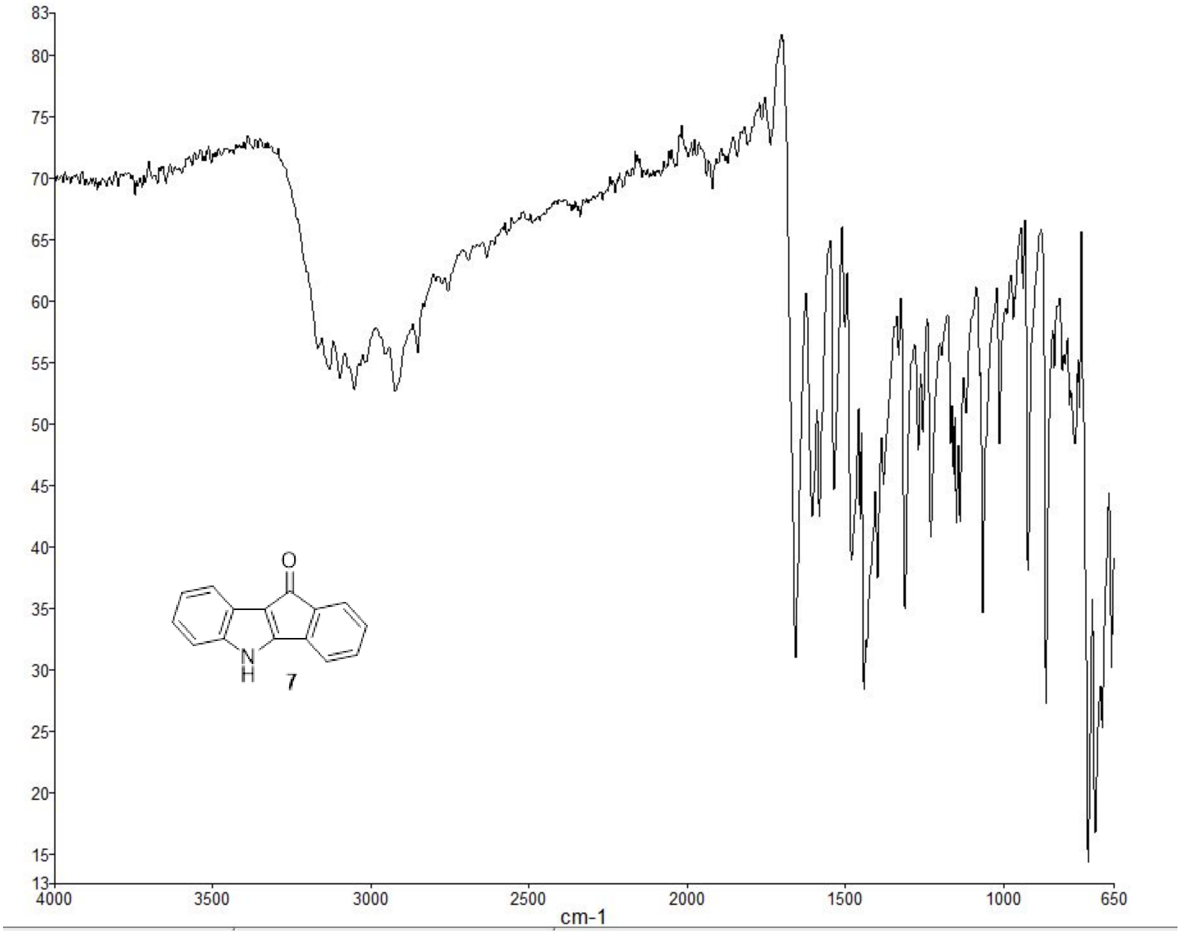

83
80
75
70
65
60
55
50
45
40
35
30
25
20
15
13
4000
3500
3000
2500
2000
1500
1000
650
cm-1
O
N
H
7

XYZ Coordinates: Created using ESIgen v0.0.5

REF: J. Pedregal, P. Gómez-Orellana, J-D. Maréchal, *J. Chem. Inf. Model.* 2018, 58, 3, 561−564

1_fluoromethanone_1

Datum	Value
B3LYP-D3(BJ)/Def2SVP Energy	-806.936073
B3LYP-D3(BJ)/Def2SVP Free Energy (Quasiharmonic)	-806.759006
Number of Imaginary Frequencies	0

Frequencies (Top 3 out of 78)

```
1    32.8989 cm-1
2    37.8854 cm-1
3    82.1722 cm-1
```

B3LYP-D3(BJ)/Def2SVP Molecular Geometry in Cartesian Coordinates

```
C    4.050289   -1.007143   -0.348527
C    4.764623    0.116734    0.063698
C    4.102926    1.306035    0.437624
C    2.712782    1.401950    0.409734
C    1.967719    0.281525    0.001228
C    2.655158   -0.905552   -0.374087
```

C	0.549836	0.003943	-0.144186
C	0.453964	-1.308141	-0.603794
N	1.695772	-1.840126	-0.736706
C	-0.535263	0.945271	0.087573
C	-1.965035	0.475735	-0.017568
O	-0.324663	2.135331	0.316283
C	-2.472806	-0.662000	0.620657
C	-3.813302	-1.029033	0.533788
C	-4.686180	-0.239401	-0.216771
C	-4.215710	0.914610	-0.854236
C	-2.872575	1.269762	-0.739922
F	-1.659465	-1.424204	1.377228
H	4.556770	-1.929240	-0.640523
H	5.855831	0.075796	0.096131
H	4.695693	2.168139	0.752782
H	2.197268	2.319851	0.690845
H	-0.427811	-1.893039	-0.852100
H	1.889769	-2.780676	-1.061469
H	-4.153210	-1.921563	1.061919
H	-5.737822	-0.523306	-0.295017
H	-4.898023	1.538342	-1.435024
H	-2.494927	2.176011	-1.217171

1_fluoromethanone_2

Datum	Value
B3LYP-D3(BJ)/Def2SVP Energy	-806.935478
B3LYP-D3(BJ)/Def2SVP Free Energy (Quasiharmonic)	-806.758353
Number of Imaginary Frequencies	0

Frequencies (Top 3 out of 78)

```
1    30.7237 cm-1
2    46.0113 cm-1
3    68.1674 cm-1
```

B3LYP-D3(BJ)/Def2SVP Molecular Geometry in Cartesian Coordinates

C	-4.137586	0.992461	0.365548
C	-4.813022	-0.149732	-0.061233
C	-4.111260	-1.294376	-0.496967
C	-2.718231	-1.326125	-0.517879
C	-2.012010	-0.186145	-0.094686
C	-2.738974	0.955252	0.342848
C	-0.604119	0.150264	0.016960
C	-0.551394	1.451360	0.516568
N	-1.811639	1.921009	0.705802
C	0.518125	-0.726673	-0.288999
C	1.905261	-0.140063	-0.239127
O	0.365475	-1.909192	-0.584082

C	2.923840	-0.779372	0.480018
C	4.229424	-0.300075	0.504884
C	4.543960	0.850328	-0.223908
C	3.553184	1.508603	-0.958460
C	2.245312	1.017849	-0.957614
F	2.632908	-1.876295	1.198738
H	-4.676121	1.879812	0.704348
H	-5.905443	-0.158750	-0.057078
H	-4.674829	-2.172162	-0.822162
H	-2.170992	-2.208790	-0.847886
H	0.313281	2.062896	0.762126
H	-2.036848	2.840364	1.068673
H	4.978940	-0.830487	1.094846
H	5.567655	1.230658	-0.214708
H	3.798171	2.403419	-1.533899
H	1.472626	1.524837	-1.538574

1_fluoromethanone_3

Datum	Value
B3LYP-D3(BJ)/Def2SVP Energy	-806.934136

B3LYP-D3(BJ)/Def2SVP Free Energy (Quasiharmonic)	-806.756907
Number of Imaginary Frequencies	0

Frequencies (Top 3 out of 78)

```
1    37.2461 cm-1
2    45.2458 cm-1
3    72.0706 cm-1
```

B3LYP-D3(BJ)/Def2SVP Molecular Geometry in Cartesian Coordinates

C	3.754551	-0.783579	-0.101460
C	3.324910	-2.069762	-0.417849
C	1.957850	-2.343643	-0.635583
C	0.991801	-1.346180	-0.531630
C	1.390012	-0.038110	-0.194964
C	2.779118	0.214935	0.000717
C	0.714884	1.237727	-0.018161
C	1.709839	2.173322	0.251557
N	2.922789	1.565768	0.275103
C	-0.693212	1.617146	-0.126139
C	-1.755615	0.552627	-0.092512
O	-1.039076	2.788045	-0.267652
C	-1.801363	-0.462215	0.870276
C	-2.822601	-1.407090	0.907190
C	-3.837396	-1.344662	-0.050288

C	-3.831270	-0.332348	-1.017159
C	-2.806251	0.612946	-1.023188
F	-0.843775	-0.524986	1.812379
H	4.811114	-0.558126	0.056646
H	4.057066	-2.875624	-0.506031
H	1.651942	-3.360069	-0.894281
H	-0.055759	-1.582405	-0.718697
H	1.601413	3.238233	0.442816
H	3.801754	2.031664	0.470288
H	-2.810356	-2.172044	1.685428
H	-4.639213	-2.085991	-0.034012
H	-4.627949	-0.279479	-1.761714
H	-2.794499	1.417299	-1.761196

1_fluoromethanone_4

Datum	Value
B3LYP-D3(BJ)/Def2SVP Energy	-806.932837
B3LYP-D3(BJ)/Def2SVP Free Energy (Quasiharmonic)	-806.755748
Number of Imaginary Frequencies	0

Frequencies (Top 3 out of 78)

1	33.2666 cm-1
2	40.8193 cm-1
3	62.2907 cm-1

B3LYP-D3(BJ)/Def2SVP Molecular Geometry in Cartesian Coordinates

C	3.902820	-0.680802	0.135151
C	3.523934	-1.971673	0.494273
C	2.163399	-2.308251	0.658324
C	1.153040	-1.370983	0.457537
C	1.500685	-0.059635	0.076826
C	2.883859	0.258011	-0.063177
C	0.771845	1.170845	-0.193321
C	1.731250	2.143352	-0.462436
N	2.972255	1.600106	-0.395818
C	-0.659196	1.477214	-0.178844
C	-1.630199	0.331396	-0.256579
O	-1.079151	2.629754	-0.142571
C	-2.721137	0.244377	0.616740
C	-3.666608	-0.772454	0.522045
C	-3.536943	-1.726255	-0.490948
C	-2.464362	-1.660628	-1.386594
C	-1.516298	-0.644325	-1.259488

F	-2.850665	1.147973	1.601765
H	4.953084	-0.406411	0.017197
H	4.290959	-2.732015	0.657850
H	1.897574	-3.326668	0.951204
H	0.110019	-1.653713	0.596723
H	1.579309	3.191086	-0.710683
H	3.835714	2.101984	-0.569831
H	-4.487640	-0.803794	1.240360
H	-4.277636	-2.524216	-0.576677
H	-2.364292	-2.403574	-2.180132
H	-0.670217	-0.594350	-1.947658

2_chloromethanone_1

Datum	Value
B3LYP-D3(BJ)/Def2SVP Energy	-1167.245257
B3LYP-D3(BJ)/Def2SVP Free Energy (Quasiharmonic)	-1167.070693
Number of Imaginary Frequencies	0

Frequencies (Top 3 out of 78)

```
1    30.1967 cm-1
2    34.1310 cm-1
3    65.8342 cm-1
```

B3LYP-D3(BJ)/Def2SVP Molecular Geometry in Cartesian Coordinates

C	4.102020	-0.875625	-0.739149
C	4.855568	0.025230	0.011970
C	4.238617	1.060728	0.746517
C	2.854300	1.222209	0.748851
C	2.070886	0.326075	0.000830
C	2.712853	-0.709865	-0.732646
C	0.646839	0.174662	-0.230924
C	0.500380	-0.919511	-1.081314
N	1.720216	-1.438178	-1.373677
C	-0.416920	1.008342	0.300249
C	-1.844456	0.631657	-0.032231
O	-0.204638	2.024019	0.956629
C	-2.476975	-0.521313	0.454922
C	-3.808357	-0.808703	0.140773
C	-4.526065	0.066959	-0.675696
C	-3.920251	1.230420	-1.160532
C	-2.594193	1.510015	-0.829835
Cl	-1.621182	-1.620702	1.524610
H	4.574721	-1.677757	-1.309228
H	5.943480	-0.072416	0.031478
H	4.862054	1.748647	1.322691
H	2.372673	2.022399	1.310627

H	-0.406757	-1.355878	-1.493010
H	1.879246	-2.237271	-1.976947
H	-4.273042	-1.709846	0.543178
H	-5.564849	-0.159956	-0.925114
H	-4.481985	1.921045	-1.792746
H	-2.115324	2.420382	-1.197293

2_chloromethanone_2

Datum	Value
B3LYP-D3(BJ)/Def2SVP Energy	-1167.244076
B3LYP-D3(BJ)/Def2SVP Free Energy (Quasiharmonic)	-1167.069291
Number of Imaginary Frequencies	0

Frequencies (Top 3 out of 78)

```
1    28.3152 cm-1
2    38.1223 cm-1
3    63.6238 cm-1
```

B3LYP-D3(BJ)/Def2SVP Molecular Geometry in Cartesian Coordinates

C	-3.765906	-0.909030	-0.141153
C	-3.276906	-2.200504	-0.321691
C	-1.895799	-2.435517	-0.488912
C	-0.973448	-1.392002	-0.473109

C	-1.433958	-0.074961	-0.282507
C	-2.835086	0.135774	-0.127139
C	-0.815927	1.239880	-0.216745
C	-1.851953	2.153346	-0.039125
N	-3.038447	1.499535	0.020092
C	0.579230	1.652070	-0.322513
C	1.643438	0.580910	-0.352464
O	0.917590	2.827190	-0.424886
C	1.936353	-0.237159	0.747493
C	2.942271	-1.204375	0.687296
C	3.669033	-1.364966	-0.494396
C	3.402583	-0.550787	-1.599577
C	2.404676	0.421584	-1.519535
Cl	1.045930	-0.047679	2.246693
H	-4.833289	-0.715759	-0.017337
H	-3.973008	-3.042171	-0.338101
H	-1.542790	-3.459077	-0.635022
H	0.087263	-1.599844	-0.610052
H	-1.790108	3.234680	0.057114
H	-3.940108	1.944131	0.152143
H	3.149273	-1.821147	1.562865
H	4.450611	-2.126072	-0.545421

H	3.974494	-0.670801	-2.521882
H	2.193658	1.066756	-2.375187

3_bromomethanone_1

Datum	Value
B3LYP-D3(BJ)/Def2SVP Energy	-3281.047184
B3LYP-D3(BJ)/Def2SVP Free Energy (Quasiharmonic)	-3280.874228
Number of Imaginary Frequencies	0

Frequencies (Top 3 out of 78)

```
1    23.8727 cm-1
2    29.1528 cm-1
3    61.1586 cm-1
```

B3LYP-D3(BJ)/Def2SVP Molecular Geometry in Cartesian Coordinates

C	-4.295030	-0.337541	1.121720
C	-5.055143	-0.058076	-0.013077
C	-4.457090	0.443788	-1.189069
C	-3.085217	0.678652	-1.260874
C	-2.295689	0.403627	-0.130841
C	-2.918299	-0.100868	1.044335
C	-0.879925	0.510734	0.165787
C	-0.718738	0.079438	1.481192

N	-1.922573	-0.280109	1.994938
C	0.166662	0.980832	-0.723515
C	1.588945	0.953475	-0.203368
O	-0.055795	1.435868	-1.841238
C	2.331072	-0.222734	-0.030266
C	3.649700	-0.190841	0.432736
C	4.242469	1.037828	0.731320
C	3.525522	2.224927	0.553235
C	2.213341	2.177967	0.081876
Br	1.576666	-1.925577	-0.479969
H	-4.753459	-0.725380	2.033598
H	-6.133677	-0.230409	0.009308
H	-5.085455	0.652092	-2.058327
H	-2.618034	1.069412	-2.164757
H	0.188921	0.009135	2.076311
H	-2.069017	-0.623905	2.937331
H	4.207643	-1.120669	0.551457
H	5.271066	1.062983	1.097616
H	3.989401	3.187233	0.779198
H	1.648161	3.101267	-0.064607

3_bromomethanone_2

Datum	Value
B3LYP-D3(BJ)/Def2SVP Energy	-3281.046092
B3LYP-D3(BJ)/Def2SVP Free Energy (Quasiharmonic)	-3280.872878
Number of Imaginary Frequencies	0

Frequencies (Top 3 out of 78)

```
1    22.3549 cm-1
2    34.2161 cm-1
3    59.1567 cm-1
```

B3LYP-D3(BJ)/Def2SVP Molecular Geometry in Cartesian Coordinates

C	-3.914618	0.919050	0.120719
C	-3.441029	2.226825	0.192377
C	-2.080554	2.518741	-0.041045
C	-1.163064	1.515783	-0.345058
C	-1.607982	0.181543	-0.416654
C	-2.988982	-0.084767	-0.185301
C	-0.989644	-1.103392	-0.702849
C	-2.006169	-2.053167	-0.638693
N	-3.180431	-1.450836	-0.327945
C	0.390865	-1.453204	-1.014973
C	1.437927	-0.368769	-0.904627
O	0.726519	-2.570550	-1.393473
C	1.873676	0.157853	0.318335

C	2.848994	1.156412	0.374850
C	3.398960	1.644957	-0.812869
C	2.988544	1.124196	-2.043707
C	2.022860	0.117418	-2.083400
Br	1.152668	-0.498363	1.964932
H	-4.966278	0.682975	0.294417
H	-4.133508	3.037698	0.429350
H	-1.739901	3.555151	0.017419
H	-0.118551	1.768062	-0.524553
H	-1.937730	-3.127792	-0.789911
H	-4.066678	-1.930500	-0.215454
H	3.174770	1.546238	1.340192
H	4.155645	2.431358	-0.770482
H	3.422445	1.500301	-2.972403
H	1.698577	-0.297409	-3.040429

4_iodomethanone_1

Datum	Value
B3LYP-D3(BJ)/Def2SVP Energy	-1004.968306
B3LYP-D3(BJ)/Def2SVP Free Energy (Quasiharmonic)	-1004.796457
Number of Imaginary Frequencies	0

Frequencies (Top 3 out of 78)

1	19.9721 cm-1
2	28.6901 cm-1
3	59.7306 cm-1

B3LYP-D3(BJ)/Def2SVP Molecular Geometry in Cartesian Coordinates

C	4.450247	-0.162178	1.173727
C	5.197872	-0.187354	-0.002763
C	4.609393	0.111406	-1.250566
C	3.259829	0.443545	-1.354121
C	2.483598	0.475876	-0.182761
C	3.095799	0.170616	1.064274
C	1.090075	0.760316	0.100558
C	0.930574	0.620291	1.478243
N	2.115542	0.273469	2.041964
C	0.057090	1.116127	-0.855123
C	-1.351203	1.279408	-0.321105
O	0.280835	1.322460	-2.043640
C	-2.173286	0.204944	0.045558
C	-3.473907	0.415520	0.515973
C	-3.963356	1.719501	0.625867
C	-3.163266	2.803790	0.253979
C	-1.871135	2.580132	-0.221800

I	-1.504569	-1.813056	-0.166109
H	4.901238	-0.393401	2.140796
H	6.258945	-0.443251	0.042742
H	5.227508	0.081591	-2.151043
H	2.800072	0.678155	-2.314096
H	0.036342	0.748186	2.083976
H	2.260869	0.118950	3.033253
H	-4.104259	-0.431620	0.790181
H	-4.977147	1.882498	0.998348
H	-3.546284	3.823335	0.332829
H	-1.240555	3.421920	-0.517493

4_iodomethanone_2

Datum	Value
B3LYP-D3(BJ)/Def2SVP Energy	-1004.967274
B3LYP-D3(BJ)/Def2SVP Free Energy (Quasiharmonic)	-1004.795176
Number of Imaginary Frequencies	0

Frequencies (Top 3 out of 78)

```
1    20.1432 cm-1
2    33.6159 cm-1
3    57.3719 cm-1
```

B3LYP-D3(BJ)/Def2SVP Molecular Geometry in Cartesian Coordinates

C	-4.066315	0.109278	-0.901534
C	-3.637333	0.725141	-2.074752
C	-2.325882	1.232602	-2.189073
C	-1.413447	1.134076	-1.141211
C	-1.813457	0.510161	0.056319
C	-3.145977	0.012789	0.147956
C	-1.184681	0.222283	1.336012
C	-2.149343	-0.410858	2.116121
N	-3.300677	-0.537071	1.411767
C	0.163525	0.490946	1.820837
C	1.163628	1.054206	0.836419
O	0.503578	0.312242	2.985382
C	1.753051	0.298350	-0.185551
C	2.672658	0.870292	-1.070119
C	3.006046	2.221066	-0.937594
C	2.437913	2.988242	0.083428
C	1.531163	2.402434	0.967591
I	1.293140	-1.771855	-0.419307
H	-5.079933	-0.283502	-0.800476
H	-4.326892	0.818628	-2.916737
H	-2.019787	1.714058	-3.120778
H	-0.407286	1.535918	-1.254290

H	-2.057528	-0.783452	3.133538
H	-4.148937	-0.970966	1.758498
H	3.127585	0.268044	-1.857795
H	3.717882	2.668772	-1.634627
H	2.702212	4.042098	0.192293
H	1.083093	2.994231	1.769117

5_fluoroethanone_1

Datum	Value
B3LYP-D3(BJ)/Def2SVP Energy	-846.23206
B3LYP-D3(BJ)/Def2SVP Free Energy (Quasiharmonic)	-846.027599
Number of Imaginary Frequencies	0

Frequencies (Top 3 out of 87)

```
1    24.3045 cm-1
2    32.4654 cm-1
3    62.7286 cm-1
```

B3LYP-D3(BJ)/Def2SVP Molecular Geometry in Cartesian Coordinates

C	-3.944536	-1.281921	0.703967
C	-4.725541	-0.161300	0.981129
C	-4.251940	1.142630	0.719392
C	-2.987896	1.358737	0.174123

C	-2.179234	0.245285	-0.116002
C	-2.675676	-1.059383	0.157017
C	-0.848482	0.076010	-0.670550
C	-0.610021	-1.297152	-0.703510
N	-1.690115	-1.961306	-0.214286
C	0.054954	1.138497	-1.092902
C	1.448879	0.746162	-1.594219
O	-0.252642	2.324401	-1.021091
C	2.371189	0.447843	-0.433751
C	2.894649	-0.829906	-0.218847
C	3.726848	-1.138064	0.853047
C	4.055460	-0.129087	1.761217
C	3.547404	1.162569	1.582560
C	2.714904	1.441175	0.497017
F	2.576112	-1.819659	-1.086973
H	-4.305407	-2.292478	0.905462
H	-5.721767	-0.295119	1.409089
H	-4.892165	1.997408	0.950480
H	-2.616488	2.362638	-0.030011
H	0.270794	-1.842251	-1.034113
H	-1.760100	-2.969318	-0.133961
H	1.395138	-0.117821	-2.267989

H	1.829792	1.606300	-2.162357
H	4.102701	-2.157006	0.960781
H	4.707759	-0.354728	2.607459
H	3.800411	1.954120	2.290916
H	2.310502	2.446547	0.357593

5_fluoroethanone_2

Datum	Value
B3LYP-D3(BJ)/Def2SVP Energy	-846.228909
B3LYP-D3(BJ)/Def2SVP Free Energy (Quasiharmonic)	-846.024425
Number of Imaginary Frequencies	0

Frequencies (Top 3 out of 87)

```
1    21.4258 cm-1
2    37.7720 cm-1
3    64.4287 cm-1
```

B3LYP-D3(BJ)/Def2SVP Molecular Geometry in Cartesian Coordinates

C	-3.547029	-1.297658	0.685728
C	-2.996877	-2.482819	0.204353
C	-1.764201	-2.479511	-0.480389
C	-1.053572	-1.300797	-0.697545
C	-1.577754	-0.082899	-0.220410
C	-2.829146	-0.117185	0.465173

C	-1.164333	1.313203	-0.240086
C	-2.165174	2.025230	0.416353
N	-3.142015	1.181941	0.830451
C	0.017811	1.989711	-0.778402
C	1.135662	1.151572	-1.385911
O	0.141069	3.209430	-0.714527
C	1.940731	0.430573	-0.324664
C	2.429565	-0.861534	-0.538847
C	3.178367	-1.556930	0.404876
C	3.467003	-0.937656	1.623298
C	3.003104	0.358021	1.872564
C	2.250278	1.029390	0.906485
F	2.154308	-1.471038	-1.714910
H	-4.501993	-1.285163	1.215127
H	-3.527298	-3.425279	0.357861
H	-1.355553	-3.423068	-0.849533
H	-0.107228	-1.344651	-1.231720
H	-2.214018	3.094672	0.606012
H	-3.974945	1.460318	1.336763
H	0.729670	0.429415	-2.106837
H	1.785684	1.845792	-1.940397
H	3.522558	-2.566419	0.173531
H	4.053885	-1.469528	2.375132

H	3.227257	0.847659	2.822469
H	1.892141	2.042288	1.103391

5_fluoroethanone_3

Datum	Value
B3LYP-D3(BJ)/Def2SVP Energy	-846.229862
B3LYP-D3(BJ)/Def2SVP Free Energy (Quasiharmonic)	-846.025928
Number of Imaginary Frequencies	0

Frequencies (Top 3 out of 87)

```
1     27.2045 cm-1
2     28.9313 cm-1
3     44.9618 cm-1
```

B3LYP-D3(BJ)/Def2SVP Molecular Geometry in Cartesian Coordinates

C	3.714131	-1.682033	0.182755
C	4.649618	-0.740271	0.607429
C	4.330287	0.633862	0.659438
C	3.070431	1.100080	0.289070
C	2.106277	0.171085	-0.142483
C	2.450328	-1.208420	-0.187941
C	0.728086	0.272720	-0.589965
C	0.317183	-1.026989	-0.880421
N	1.336416	-1.895376	-0.644026

C	-0.036696	1.510003	-0.722240
C	-1.495936	1.446229	-1.193382
O	0.472852	2.603245	-0.502428
C	-2.387744	0.461597	-0.478745
C	-2.444344	0.434909	0.918799
C	-3.247114	-0.450267	1.628839
C	-4.045810	-1.352030	0.919111
C	-4.025576	-1.350038	-0.478634
C	-3.203988	-0.449728	-1.163869
F	-1.670565	1.302028	1.604654
H	3.955411	-2.746077	0.139706
H	5.647252	-1.071976	0.904549
H	5.089142	1.343957	0.996981
H	2.819191	2.159577	0.325063
H	-0.641527	-1.388940	-1.240573
H	1.282769	-2.898538	-0.778134
H	-1.882924	2.471080	-1.085913
H	-1.494601	1.211191	-2.270096
H	-3.237029	-0.422740	2.719844
H	-4.681773	-2.054324	1.462162
H	-4.648673	-2.050973	-1.037777
H	-3.184679	-0.453063	-2.256644

5_fluoroethanone_4

Datum	Value
B3LYP-D3(BJ)/Def2SVP Energy	-846.230328
B3LYP-D3(BJ)/Def2SVP Free Energy (Quasiharmonic)	-846.026887
Number of Imaginary Frequencies	0

Frequencies (Top 3 out of 87)

```
1    19.5214 cm-1
2    27.9992 cm-1
3    54.4163 cm-1
```

B3LYP-D3(BJ)/Def2SVP Molecular Geometry in Cartesian Coordinates

C	4.860062	0.277876	0.039208
C	5.099436	-1.074620	-0.196250
C	4.035485	-1.983136	-0.386302
C	2.707784	-1.562691	-0.346047
C	2.437566	-0.202502	-0.110537
C	3.524632	0.695736	0.078601
C	1.223913	0.585502	-0.001557
C	1.629063	1.895225	0.243140
N	2.986952	1.955660	0.289821
C	-0.145051	0.094857	-0.125578
C	-1.271792	1.128152	0.025351

O	-0.394898	-1.084591	-0.340380
C	-2.642147	0.537241	-0.133438
C	-3.177021	-0.275428	0.871093
C	-4.429322	-0.871239	0.776238
C	-5.191451	-0.653858	-0.376295
C	-4.689505	0.150229	-1.403497
C	-3.425492	0.736048	-1.277343
F	-2.441737	-0.480267	1.983502
H	5.679489	0.983970	0.187456
H	6.129139	-1.437710	-0.234018
H	4.261558	-3.036561	-0.568373
H	1.882588	-2.259389	-0.491541
H	1.032334	2.792820	0.389093
H	3.522658	2.800273	0.454818
H	-1.163157	1.598003	1.016667
H	-1.114128	1.926114	-0.716537
H	-4.790008	-1.493299	1.597423
H	-6.177443	-1.114937	-0.466845
H	-5.281744	0.322737	-2.304601
H	-3.032084	1.363129	-2.081478

5_fluoroethanone_5

Datum	Value
B3LYP-D3(BJ)/Def2SVP Energy	-846.228332
B3LYP-D3(BJ)/Def2SVP Free Energy (Quasiharmonic)	-846.024675
Number of Imaginary Frequencies	0

Frequencies (Top 3 out of 87)

```
1    23.8612 cm-1
2    31.9306 cm-1
3    57.4462 cm-1
```

B3LYP-D3(BJ)/Def2SVP Molecular Geometry in Cartesian Coordinates

C	4.759038	-0.168686	0.036816
C	4.711223	-1.543270	0.254339
C	3.476351	-2.220438	0.326025
C	2.266340	-1.544912	0.182474
C	2.276364	-0.152796	-0.039928
C	3.541250	0.505052	-0.106673
C	1.270641	0.881377	-0.232511
C	1.965514	2.075567	-0.400721
N	3.301063	1.851168	-0.326849
C	-0.193403	0.830238	-0.270275
C	-0.852778	-0.539120	-0.091246
O	-0.868067	1.837993	-0.439251
C	-2.351077	-0.495836	-0.161667
C	-3.087307	0.055183	0.891642

C	-4.474512	0.143039	0.880646
C	-5.169501	-0.337640	-0.234021
C	-4.468060	-0.893771	-1.307518
C	-3.071752	-0.968135	-1.265717
F	-2.414618	0.512254	1.968076
H	5.708807	0.366938	-0.020086
H	5.641674	-2.103316	0.371358
H	3.468297	-3.299158	0.498562
H	1.332277	-2.101949	0.245496
H	1.561493	3.070736	-0.569927
H	4.016402	2.563754	-0.417523
H	-0.452471	-1.216754	-0.861215
H	-0.517914	-0.941340	0.879272
H	-4.990140	0.582599	1.736397
H	-6.259717	-0.276147	-0.258585
H	-5.007268	-1.271202	-2.178886
H	-2.522780	-1.401680	-2.105570

5_fluoroethanone_6

Datum	Value
B3LYP-D3(BJ)/Def2SVP Energy	-846.226189
B3LYP-D3(BJ)/Def2SVP Free Energy (Quasiharmonic)	-846.022129

Number of Imaginary Frequencies 0

Frequencies (Top 3 out of 87)

1	26.8917 cm-1
2	29.0627 cm-1
3	60.4351 cm-1

B3LYP-D3(BJ)/Def2SVP Molecular Geometry in Cartesian Coordinates

C	3.383365	-1.290572	0.682384
C	2.977042	-2.432323	-0.003147
C	1.855255	-2.400622	-0.858071
C	1.110574	-1.237502	-1.036107
C	1.478924	-0.068550	-0.342063
C	2.631625	-0.124581	0.497776
C	0.993476	1.300641	-0.276986
C	1.864755	1.982716	0.566272
N	2.822689	1.139439	1.029017
C	-0.158677	1.978775	-0.883459
C	-1.284837	1.144701	-1.495205
O	-0.235835	3.201182	-0.901766
C	-1.910899	0.104049	-0.594028
C	-2.152158	0.353623	0.761162
C	-2.738495	-0.577869	1.612124
C	-3.124995	-1.816698	1.095188

C	-2.920187	-2.097951	-0.258843
C	-2.320366	-1.144958	-1.085376
F	-1.798787	1.551435	1.273185
H	4.257428	-1.298285	1.336746
H	3.538610	-3.361611	0.116474
H	1.565348	-3.307681	-1.393547
H	0.256426	-1.245775	-1.709700
H	1.827597	3.026149	0.869568
H	3.561879	1.395607	1.673550
H	-2.043296	1.869183	-1.830408
H	-0.897236	0.645802	-2.398604
H	-2.886401	-0.318518	2.661913
H	-3.586367	-2.557539	1.751609
H	-3.223277	-3.061953	-0.672411
H	-2.160185	-1.371100	-2.142573

6_chloroethanone_1

Datum	Value
B3LYP-D3(BJ)/Def2SVP Energy	-1206.542059
B3LYP-D3(BJ)/Def2SVP Free Energy (Quasiharmonic)	-1206.339713
Number of Imaginary Frequencies	0

Frequencies (Top 3 out of 87)

1 22.5095 cm-1
2 30.4876 cm-1
3 57.1416 cm-1

B3LYP-D3(BJ)/Def2SVP Molecular Geometry in Cartesian Coordinates

C	4.088684	-1.193175	0.977616
C	4.944434	-0.094076	0.927806
C	4.536709	1.123779	0.341752
C	3.265494	1.273107	-0.209332
C	2.382115	0.179491	-0.172966
C	2.812955	-1.037711	0.423718
C	1.021363	-0.040093	-0.628734
C	0.702678	-1.354420	-0.291020
N	1.761815	-1.937357	0.329409
C	0.163914	0.932766	-1.292208
C	-1.272656	0.521758	-1.627870
O	0.546989	2.070538	-1.549514
C	-2.163584	0.608304	-0.406985
C	-2.914261	-0.470443	0.087952
C	-3.719858	-0.353085	1.225300
C	-3.786653	0.866341	1.898830
C	-3.050659	1.959378	1.430269
C	-2.253885	1.823466	0.294581

Cl	-2.878172	-2.035747	-0.719652
H	4.398915	-2.137097	1.430259
H	5.948455	-0.177308	1.350352
H	5.234720	1.964209	0.321182
H	2.945046	2.210196	-0.663900
H	-0.219523	-1.909573	-0.448499
H	1.776767	-2.891391	0.671526
H	-1.627542	1.227353	-2.393098
H	-1.303169	-0.487727	-2.053882
H	-4.286761	-1.217754	1.572747
H	-4.414902	0.959027	2.787272
H	-3.097631	2.918509	1.950236
H	-1.674807	2.674139	-0.072117

6_chloroethanone_2

Datum	Value
B3LYP-D3(BJ)/Def2SVP Energy	-1206.541041
B3LYP-D3(BJ)/Def2SVP Free Energy (Quasiharmonic)	-1206.339864
Number of Imaginary Frequencies	0

Frequencies (Top 3 out of 87)

1	12.3750 cm-1
2	24.5628 cm-1
3	48.8662 cm-1

B3LYP-D3(BJ)/Def2SVP Molecular Geometry in Cartesian Coordinates

C	4.939439	-0.313930	0.075873
C	5.202101	1.035371	0.304983
C	4.163064	1.991272	0.313441
C	2.837556	1.622722	0.093247
C	2.544162	0.267212	-0.141333
C	3.606367	-0.679255	-0.145376
C	1.324449	-0.475448	-0.400555
C	1.702598	-1.807865	-0.547167
N	3.049192	-1.923333	-0.396104
C	-0.021656	0.078221	-0.501774
C	-1.159463	-0.897225	-0.838214
O	-0.245229	1.273316	-0.354622
C	-2.524369	-0.316015	-0.604739
C	-3.036110	-0.141692	0.691825
C	-4.295802	0.412509	0.925557
C	-5.078169	0.814447	-0.159774
C	-4.595321	0.658086	-1.461535
C	-3.332460	0.099417	-1.672500

Cl	-2.068279	-0.632789	2.074647
H	5.739492	-1.056914	0.068664
H	6.230727	1.358465	0.481326
H	4.406972	3.040476	0.496885
H	2.031320	2.355782	0.099382
H	1.094950	-2.686359	-0.751956
H	3.564563	-2.794087	-0.455887
H	-1.046154	-1.176022	-1.898459
H	-1.027259	-1.819223	-0.252984
H	-4.653787	0.526589	1.949708
H	-6.064357	1.249111	0.017581
H	-5.201582	0.970746	-2.314359
H	-2.954973	-0.021309	-2.690949

6_chloroethanone_3

Datum	Value
B3LYP-D3(BJ)/Def2SVP Energy	-1206.539062
B3LYP-D3(BJ)/Def2SVP Free Energy (Quasiharmonic)	-1206.336867
Number of Imaginary Frequencies	0

Frequencies (Top 3 out of 87)

1	24.4495 cm-1
2	33.5904 cm-1
3	54.6387 cm-1

B3LYP-D3(BJ)/Def2SVP Molecular Geometry in Cartesian Coordinates

C	1.525538	-2.605452	-0.220371
C	0.955785	-1.367506	-0.509475
C	1.681962	-0.193047	-0.229157
C	2.985578	-0.331131	0.334772
C	1.453066	1.237013	-0.376861
C	2.603910	1.866500	0.090219
N	3.502977	0.942272	0.509216
C	0.309353	2.007724	-0.870689
C	-0.963886	1.263524	-1.248620
O	0.351725	3.231030	-0.959388
C	-1.696803	0.695114	-0.051363
C	-2.506727	-0.449842	-0.140545
C	-3.192064	-0.964503	0.962585
C	-3.081528	-0.326043	2.198547
C	-2.293030	0.821396	2.317657
C	-1.615246	1.319347	1.204163
Cl	-2.679963	-1.287840	-1.677557
H	4.562281	-1.639173	1.061028

H	3.229873	-3.697650	0.559735
H	0.960224	-3.514852	-0.437134
H	-0.038320	-1.332428	-0.949570
H	2.811647	2.932298	0.145271
H	4.415977	1.152967	0.896330
H	-1.612137	1.982253	-1.774112
H	-0.728358	0.460785	-1.961342
H	-3.803957	-1.859781	0.844922
H	-3.613993	-0.726851	3.063686
H	-2.204031	1.331026	3.279310
H	-1.005486	2.219446	1.305868

6_chloroethanone_4

Datum	Value
B3LYP-D3(BJ)/Def2SVP Energy	-1206.540403
B3LYP-D3(BJ)/Def2SVP Free Energy (Quasiharmonic)	-1206.338565
Number of Imaginary Frequencies	0

Frequencies (Top 3 out of 87)

```
1    21.9880 cm-1
2    30.7431 cm-1
3    46.1007 cm-1
```

B3LYP-D3(BJ)/Def2SVP Molecular Geometry in Cartesian Coordinates

C	3.812342	1.659688	0.512571
C	4.767147	0.656850	0.670795
C	4.462261	-0.691983	0.387438
C	3.198397	-1.071210	-0.060647
C	2.214749	-0.079514	-0.228408
C	2.544679	1.273019	0.062646
C	0.825474	-0.089283	-0.656080
C	0.395974	1.236152	-0.605386
N	1.413402	2.034007	-0.184422
C	0.082017	-1.277684	-1.067083
C	-1.395165	-1.171899	-1.479029
O	0.629570	-2.372076	-1.146078
C	-2.290033	-0.248964	-0.693299
C	-2.467291	-0.381875	0.694141
C	-3.286020	0.481899	1.424529
C	-3.962484	1.509393	0.763785
C	-3.820058	1.658618	-0.618350
C	-2.994638	0.785419	-1.329676
Cl	-1.637190	-1.667352	1.553700
H	4.041911	2.704750	0.730354
H	5.768364	0.920035	1.019932
H	5.235758	-1.451968	0.522353
H	2.958252	-2.110028	-0.283699

H	-0.572162	1.664443	-0.845044
H	1.346828	3.038405	-0.066569
H	-1.407998	-0.865285	-2.537828
H	-1.774600	-2.204295	-1.441490
H	-3.387854	0.343867	2.501798
H	-4.601866	2.188029	1.332377
H	-4.350058	2.455716	-1.143760
H	-2.878812	0.908112	-2.409424

6_chloroethanone_5

Datum	Value
B3LYP-D3(BJ)/Def2SVP Energy	-1206.539034
B3LYP-D3(BJ)/Def2SVP Free Energy (Quasiharmonic)	-1206.337413
Number of Imaginary Frequencies	0

Frequencies (Top 3 out of 87)

```
1    21.9787 cm-1
2    27.0236 cm-1
3    57.1083 cm-1
```

B3LYP-D3(BJ)/Def2SVP Molecular Geometry in Cartesian Coordinates

C	4.867635	-0.158596	0.113010
C	4.817300	-1.523436	0.384804
C	3.586449	-2.211525	0.401324

C	2.382876	-1.556524	0.149260
C	2.395293	-0.174275	-0.127705
C	3.656305	0.494366	-0.139682
C	1.396211	0.838779	-0.433053
C	2.090805	2.031634	-0.610563
N	3.420136	1.826388	-0.436215
C	-0.061940	0.771251	-0.561591
C	-0.724900	-0.588382	-0.328536
O	-0.728823	1.756495	-0.850126
C	-2.224284	-0.538048	-0.375507
C	-2.979857	-0.037418	0.697406
C	-4.373793	0.030842	0.655696
C	-5.046654	-0.405512	-0.487980
C	-4.322451	-0.905079	-1.573576
C	-2.928551	-0.966913	-1.509677
Cl	-2.160082	0.522165	2.148132
H	5.814356	0.385204	0.096393
H	5.742639	-2.067461	0.586992
H	3.576493	-3.282589	0.616285
H	1.451822	-2.121809	0.169128
H	1.690208	3.013503	-0.850689
H	4.133972	2.542307	-0.510156
H	-0.376934	-0.972937	0.642508

H	-0.341926	-1.281315	-1.094883
H	-4.920622	0.423844	1.513945
H	-6.137022	-0.353743	-0.525294
H	-4.842248	-1.248196	-2.470628
H	-2.362372	-1.357278	-2.359114

6_chloroethanone_6

Datum	Value
B3LYP-D3(BJ)/Def2SVP Energy	-1206.538431
B3LYP-D3(BJ)/Def2SVP Free Energy (Quasiharmonic)	-1206.336509
Number of Imaginary Frequencies	0

Frequencies (Top 3 out of 87)

1	15.8785 cm-1
2	31.1729 cm-1
3	40.2195 cm-1

B3LYP-D3(BJ)/Def2SVP Molecular Geometry in Cartesian Coordinates

C	4.795199	0.646254	0.178847
C	5.124612	-0.410203	1.026058
C	4.173517	-1.394712	1.371062
C	2.871089	-1.348602	0.877923
C	2.512048	-0.293410	0.020275

C	3.485604	0.687875	-0.313163
C	1.289327	0.079873	-0.669427
C	1.581759	1.248536	-1.372274
N	2.876215	1.601928	-1.159315
C	0.021214	-0.636271	-0.612433
C	-1.185065	-0.056285	-1.364611
O	-0.114378	-1.664414	0.041201
C	-2.205607	0.469484	-0.383510
C	-3.305387	-0.286206	0.056817
C	-4.208513	0.210043	1.002554
C	-4.018267	1.484485	1.537745
C	-2.930528	2.257637	1.121208
C	-2.041880	1.748603	0.174047
Cl	-3.604111	-1.892576	-0.589480
H	5.527131	1.410196	-0.090857
H	6.137395	-0.476848	1.430220
H	4.468101	-2.208240	2.038375
H	2.132223	-2.105245	1.139970
H	0.949415	1.853821	-2.017156
H	3.326569	2.414961	-1.564091
H	-0.886720	0.743921	-2.052321
H	-1.616412	-0.872949	-1.958177
H	-5.053545	-0.406774	1.311415

H	-4.723039	1.870401	2.277466
H	-2.774420	3.256941	1.532939
H	-1.190023	2.352832	-0.147727

6_chloroethanone_7

Datum	Value
B3LYP-D3(BJ)/Def2SVP Energy	-1206.537021
B3LYP-D3(BJ)/Def2SVP Free Energy (Quasiharmonic)	-1206.335559
Number of Imaginary Frequencies	0

Frequencies (Top 3 out of 87)

```
1     20.3172 cm-1
2     25.2485 cm-1
3     30.0066 cm-1
```

B3LYP-D3(BJ)/Def2SVP Molecular Geometry in Cartesian Coordinates

C	-4.588843	-0.361221	-0.094578
C	-4.430540	-1.620155	0.477678
C	-3.164126	-2.055492	0.919753
C	-2.036736	-1.245004	0.810775
C	-2.160081	0.044444	0.253870
C	-3.452083	0.447777	-0.201291
C	-1.260587	1.150446	-0.049927

C	-2.040137	2.127300	-0.664456
N	-3.329033	1.715619	-0.743968
C	0.171529	1.372359	0.170182
C	0.937185	0.471816	1.144876
O	0.761197	2.305613	-0.361964
C	2.323867	0.142334	0.662340
C	2.528878	-0.751054	-0.402262
C	3.803675	-1.064914	-0.875670
C	4.918365	-0.469245	-0.280012
C	4.746695	0.429817	0.775638
C	3.461358	0.727553	1.235298
Cl	1.136269	-1.492354	-1.179227
H	-5.560189	-0.015122	-0.453746
H	-5.294714	-2.280663	0.577787
H	-3.063117	-3.053373	1.352727
H	-1.073422	-1.630878	1.141895
H	-1.726904	3.101196	-1.032568
H	-4.090159	2.257534	-1.137281
H	1.002630	1.042286	2.085870
H	0.376486	-0.440933	1.368449
H	3.915251	-1.766592	-1.703435
H	5.918803	-0.710162	-0.646046
H	5.614137	0.900632	1.243064

H	3.327769	1.431275	2.060348

6_chloroethanone_8

Datum	Value
B3LYP-D3(BJ)/Def2SVP Energy	-1206.535545
B3LYP-D3(BJ)/Def2SVP Free Energy (Quasiharmonic)	-1206.333779
Number of Imaginary Frequencies	0

Frequencies (Top 3 out of 87)

```
1     18.8268 cm-1
2     29.9068 cm-1
3     61.4577 cm-1
```

B3LYP-D3(BJ)/Def2SVP Molecular Geometry in Cartesian Coordinates

C	-3.564969	0.679347	-0.965232
C	-3.502797	1.887146	-0.274927
C	-2.537280	2.096614	0.732649
C	-1.605721	1.114527	1.058286
C	-1.618295	-0.106923	0.358511
C	-2.625455	-0.303964	-0.633171
C	-0.862444	-1.344968	0.425275
C	-1.448376	-2.209635	-0.491904
N	-2.478251	-1.589022	-1.126038
C	0.318118	-1.749302	1.201112
C	1.290339	-0.680444	1.712694

O	0.549498	-2.928948	1.430206
C	1.686444	0.413387	0.747720
C	2.153553	0.153735	-0.552611
C	2.517412	1.178348	-1.430392
C	2.438436	2.506867	-1.010669
C	2.007220	2.797903	0.285773
C	1.640498	1.760244	1.143275
Cl	2.324300	-1.500221	-1.117710
H	-4.320912	0.499800	-1.732374
H	-4.219105	2.678207	-0.508191
H	-2.523896	3.046986	1.271277
H	-0.886881	1.295013	1.855053
H	-1.152497	-3.225082	-0.744031
H	-3.043201	-2.001978	-1.859325
H	0.840483	-0.218655	2.607164
H	2.177166	-1.235560	2.055555
H	2.865812	0.925698	-2.432739
H	2.721676	3.308619	-1.696006
H	1.952921	3.832579	0.630412
H	1.307021	1.995561	2.156560

7_bromoethanone_1

Datum	Value
B3LYP-D3(BJ)/Def2SVP Energy	-3320.343099
B3LYP-D3(BJ)/Def2SVP Free Energy (Quasiharmonic)	-3320.142456

Datum	Value
Number of Imaginary Frequencies	0

Frequencies (Top 3 out of 87)

1	17.0812 cm-1
2	28.1368 cm-1
3	45.0983 cm-1

B3LYP-D3(BJ)/Def2SVP Molecular Geometry in Cartesian Coordinates

C	-4.344495	-1.281453	1.115244
C	-5.313036	-0.306782	0.881235
C	-5.021000	0.846447	0.121170
C	-3.755344	1.053748	-0.423853
C	-2.759600	0.085484	-0.203497
C	-3.075091	-1.068453	0.565956
C	-1.367494	-0.043597	-0.594366
C	-0.918960	-1.247102	-0.052944
N	-1.929049	-1.846909	0.629972
C	-0.598597	0.910067	-1.382226
C	0.890112	0.629663	-1.604703
O	-1.098613	1.938172	-1.830118

C	1.706123	1.011338	-0.387152
C	2.596271	0.147514	0.271636
C	3.321171	0.548526	1.399313
C	3.164446	1.842763	1.895455
C	2.288220	2.727705	1.259682
C	1.574954	2.310423	0.137642
Br	2.883697	-1.646104	-0.351924
H	-4.565195	-2.174415	1.703472
H	-6.315885	-0.438291	1.294177
H	-5.805669	1.589329	-0.041230
H	-3.523265	1.941256	-1.012069
H	0.062600	-1.712997	-0.109142
H	-1.851873	-2.735470	1.111761
H	1.054856	-0.422287	-1.865700
H	1.199827	1.245271	-2.461992
H	4.003561	-0.151716	1.882727
H	3.729618	2.155300	2.776135
H	2.160381	3.743990	1.638328
H	0.887821	2.998927	-0.359537

7_bromoethanone_2

Datum	Value
B3LYP-D3(BJ)/Def2SVP Energy	-3320.340489

B3LYP-D3(BJ)/Def2SVP Free Energy (Quasiharmonic)	-3320.139892
Number of Imaginary Frequencies	0

Frequencies (Top 3 out of 87)

```
1     22.4556 cm-1
2     29.6520 cm-1
3     47.1146 cm-1
```

B3LYP-D3(BJ)/Def2SVP Molecular Geometry in Cartesian Coordinates

C	3.780206	1.804251	0.646062
C	2.862198	2.850876	0.678828
C	1.527414	2.653117	0.268668
C	1.079016	1.412148	-0.177781
C	1.978753	0.328819	-0.218893
C	3.324007	0.560064	0.196885
C	1.909001	-1.072371	-0.606990
C	3.184489	-1.595461	-0.411367
N	4.014317	-0.633692	0.062424
C	0.808611	-1.905298	-1.097745
C	-0.583989	-1.295889	-1.182811
O	0.984932	-3.076444	-1.418862
C	-1.212498	-1.039125	0.171130
C	-2.203006	-0.062740	0.374714
C	-2.785449	0.158071	1.625506

C	-2.382899	-0.613590	2.716856
C	-1.409637	-1.600900	2.545222
C	-0.839499	-1.805541	1.288460
Br	-2.808061	1.024664	-1.084636
H	4.817344	1.944476	0.957522
H	3.180700	3.836885	1.024503
H	0.827826	3.491587	0.301189
H	0.041939	1.302491	-0.487487
H	3.527413	-2.612410	-0.585514
H	4.993357	-0.768864	0.287696
H	-0.540772	-0.361356	-1.760564
H	-1.209475	-1.998935	-1.755098
H	-3.547349	0.929954	1.741420
H	-2.834146	-0.441034	3.696283
H	-1.091685	-2.214210	3.390916
H	-0.083004	-2.583038	1.164848

7_bromoethanone_3

Datum	Value
B3LYP-D3(BJ)/Def2SVP Energy	-3320.340918

B3LYP-D3(BJ)/Def2SVP Free Energy (Quasiharmonic)	-3320.140721
Number of Imaginary Frequencies	0

Frequencies (Top 3 out of 87)

```
1    17.2257 cm-1
2    25.3536 cm-1
3    56.2062 cm-1
```

B3LYP-D3(BJ)/Def2SVP Molecular Geometry in Cartesian Coordinates

C	5.110882	0.082515	0.205937
C	5.067367	-0.347004	1.529868
C	3.851045	-0.750115	2.118701
C	2.655332	-0.732860	1.403788
C	2.660950	-0.301053	0.061797
C	3.907421	0.098396	-0.507452
C	1.666370	-0.143520	-0.988740
C	2.349294	0.327909	-2.106070
N	3.667329	0.470838	-1.819623
C	0.221438	-0.384664	-1.015617
C	-0.433976	-0.872135	0.278779
O	-0.441717	-0.211404	-2.029907
C	-1.928614	-0.982639	0.187781
C	-2.773253	0.137563	0.255500
C	-4.161748	0.029171	0.151321

C	-4.739223	-1.229664	-0.031720
C	-3.925986	-2.363173	-0.107242
C	-2.540311	-2.232126	0.002100
Br	-2.027892	1.886541	0.502553
H	6.046645	0.395227	-0.261709
H	5.986939	-0.372918	2.118768
H	3.846374	-1.084182	3.158823
H	1.735825	-1.054606	1.891486
H	1.948160	0.568681	-3.087578
H	4.370783	0.802877	-2.469673
H	0.004718	-1.853170	0.522535
H	-0.138548	-0.189491	1.090246
H	-4.784670	0.922616	0.212453
H	-5.824799	-1.318168	-0.113563
H	-4.369874	-3.350747	-0.249763
H	-1.904830	-3.119306	-0.057145

7_bromoethanone_4

Datum	Value
B3LYP-D3(BJ)/Def2SVP Energy	-3320.342136
B3LYP-D3(BJ)/Def2SVP Free Energy (Quasiharmonic)	-3320.141529

Number of Imaginary Frequencies 0

Frequencies (Top 3 out of 87)

1	19.7126 cm-1
2	29.2589 cm-1
3	45.7734 cm-1

B3LYP-D3(BJ)/Def2SVP Molecular Geometry in Cartesian Coordinates

C	-4.072677	1.369130	-1.026252
C	-4.966398	0.312141	-0.864246
C	-4.606315	-0.842437	-0.136316
C	-3.347093	-0.968880	0.447135
C	-2.424434	0.082777	0.298694
C	-2.809299	1.236296	-0.438918
C	-1.057036	0.304546	0.740336
C	-0.694196	1.561491	0.257527
N	-1.730831	2.106236	-0.433032
C	-0.273909	-0.632988	1.541039
C	1.181401	-0.317520	1.925370
O	-0.771841	-1.667537	1.972115
C	2.057271	0.388046	0.922953
C	2.334436	-0.136423	-0.350991
C	3.134364	0.539618	-1.275274
C	3.689926	1.773947	-0.931330

C	3.448129	2.315288	0.333833
C	2.644289	1.624383	1.241963
Br	1.609043	-1.830116	-0.869026
H	-4.344544	2.264827	-1.588409
H	-5.962370	0.379140	-1.308256
H	-5.332646	-1.651765	-0.029110
H	-3.065281	-1.856168	1.012639
H	0.238073	2.106729	0.364028
H	-1.710617	3.015785	-0.879730
H	1.136133	0.287800	2.845701
H	1.616782	-1.288032	2.207581
H	3.320411	0.100800	-2.256465
H	4.313638	2.305169	-1.653458
H	3.883638	3.276496	0.614474
H	2.450074	2.053454	2.228074

7_bromoethanone_5

Datum	Value
B3LYP-D3(BJ)/Def2SVP Energy	-3320.340282
B3LYP-D3(BJ)/Def2SVP Free Energy (Quasiharmonic)	-3320.139998
Number of Imaginary Frequencies	0

Frequencies (Top 3 out of 87)

1	13.6189 cm-1
2	28.7065 cm-1
3	40.9796 cm-1

B3LYP-D3(BJ)/Def2SVP Molecular Geometry in Cartesian Coordinates

C	-5.264298	0.411126	-0.099866
C	-5.518740	-0.604768	-1.019420
C	-4.494146	-1.476109	-1.448406
C	-3.191336	-1.354622	-0.969283
C	-2.906937	-0.337994	-0.040013
C	-3.954023	0.528807	0.377437
C	-1.707953	0.087850	0.660807
C	-2.086027	1.171834	1.452833
N	-3.409345	1.427309	1.282069
C	-0.384112	-0.509095	0.535461
C	0.781179	0.122356	1.310970
O	-0.172704	-1.472536	-0.192194
C	1.707546	0.847690	0.363345
C	2.860503	0.270682	-0.195643
C	3.665557	0.964567	-1.105311
C	3.321495	2.263188	-1.483345
C	2.178591	2.862356	-0.946429
C	1.389855	2.157309	-0.037946

Br	3.409947	-1.502356	0.281778
H	-6.052732	1.087812	0.235582
H	-6.529530	-0.728473	-1.415048
H	-4.731125	-2.260955	-2.170739
H	-2.396236	-2.024096	-1.295958
H	-1.495812	1.779498	2.134303
H	-3.919749	2.167126	1.750645
H	0.423288	0.818561	2.078686
H	1.312618	-0.695213	1.815441
H	4.557462	0.487203	-1.513400
H	3.950534	2.802850	-2.194641
H	1.901783	3.878972	-1.233534
H	0.495467	2.627256	0.378335

7_bromoethanone_6

Datum	Value
B3LYP-D3(BJ)/Def2SVP Energy	-3320.340918
B3LYP-D3(BJ)/Def2SVP Free Energy (Quasiharmonic)	-3320.14072
Number of Imaginary Frequencies	0

Frequencies (Top 3 out of 87)

```
1	17.2163 cm-1
2	25.3826 cm-1
3	56.1915 cm-1
```

B3LYP-D3(BJ)/Def2SVP Molecular Geometry in Cartesian Coordinates

C	5.110910	-0.082178	0.205921
C	5.067385	0.347888	1.529667
C	3.851036	0.751124	2.118362
C	2.655312	0.733434	1.403480
C	2.660932	0.301040	0.061683
C	3.907425	-0.098536	-0.507421
C	1.666326	0.142833	-0.988730
C	2.349265	-0.329001	-2.105871
N	3.667374	-0.471386	-1.819471
C	0.221364	0.383815	-1.015746
C	-0.433998	0.872329	0.278285
O	-0.441819	0.209707	-2.029856
C	-1.928626	0.982777	0.187315
C	-2.773244	-0.137378	0.255514
C	-4.161750	-0.029062	0.151304
C	-4.739252	1.229679	-0.032221
C	-3.926027	2.363179	-0.108217
C	-2.540355	2.232195	0.001100

Br	-2.027825	-1.886216	0.503376
H	6.046701	-0.394947	-0.261630
H	5.986972	0.374144	2.118524
H	3.846341	1.085623	3.158340
H	1.735800	1.055306	1.891083
H	1.948176	-0.570263	-3.087277
H	4.370668	-0.804576	-2.469102
H	-0.138518	0.190289	1.090256
H	0.004727	1.853535	0.521224
H	-4.784626	-0.922509	0.212804
H	-5.824823	1.318152	-0.114108
H	-4.369960	3.350673	-0.251130
H	-1.904867	3.119338	-0.058549

7_bromoethanone_7

Datum	Value
B3LYP-D3(BJ)/Def2SVP Energy	-3320.33687
B3LYP-D3(BJ)/Def2SVP Free Energy (Quasiharmonic)	-3320.136429
Number of Imaginary Frequencies	0

Frequencies (Top 3 out of 87)

1	19.4036 cm-1
2	28.7654 cm-1
3	55.7363 cm-1

B3LYP-D3(BJ)/Def2SVP Molecular Geometry in Cartesian Coordinates

C	-3.854349	-0.109159	-1.194210
C	-4.132900	1.129623	-0.622351
C	-3.342215	1.634788	0.431639
C	-2.249809	0.924523	0.922236
C	-1.918012	-0.315824	0.345005
C	-2.754527	-0.817897	-0.696609
C	-0.914119	-1.336098	0.590670
C	-1.201381	-2.382736	-0.278244
N	-2.277172	-2.068620	-1.047373
C	0.244716	-1.398278	1.492497
C	0.919792	-0.101744	1.955837
O	0.695739	-2.472435	1.866846
C	1.145254	0.978372	0.922591
C	1.795602	0.759056	-0.304754
C	1.983593	1.780606	-1.240276
C	1.535486	3.071073	-0.953947
C	0.916759	3.331254	0.270769
C	0.731110	2.295540	1.186141

Br	2.509033	-0.963474	-0.742674
H	-4.472757	-0.519467	-1.995128
H	-4.982732	1.711822	-0.985833
H	-3.595898	2.600463	0.875069
H	-1.672254	1.327523	1.751656
H	-0.663624	-3.319068	-0.405988
H	-2.660913	-2.655983	-1.778724
H	0.309251	0.314405	2.774397
H	1.874229	-0.417421	2.404628
H	2.486282	1.564263	-2.183882
H	1.681508	3.868822	-1.685416
H	0.575265	4.339408	0.514323
H	0.249766	2.508035	2.143366

7_bromoethanone_8

**Datum**	**Value**
B3LYP-D3(BJ)/Def2SVP Energy	-3320.33687
B3LYP-D3(BJ)/Def2SVP Free Energy (Quasiharmonic)	-3320.13643
Number of Imaginary Frequencies	0

Frequencies (Top 3 out of 87)

```
1	19.3729 cm-1
2	28.7228 cm-1
3	55.7104 cm-1
```

B3LYP-D3(BJ)/Def2SVP Molecular Geometry in Cartesian Coordinates

C	3.854952	-0.109049	-1.193728
C	4.133238	1.129848	-0.621961
C	3.342267	1.635061	0.431763
C	2.249784	0.924769	0.922187
C	1.918251	-0.315690	0.345063
C	2.755110	-0.817831	-0.696256
C	0.914328	-1.335972	0.590499
C	1.201987	-2.382757	-0.278103
N	2.277833	-2.068582	-1.047116
C	-0.244674	-1.398203	1.492077
C	-0.919651	-0.101714	1.955716
O	-0.695914	-2.472419	1.866032
C	-1.145457	0.978385	0.922458
C	-1.795861	0.758936	-0.304811
C	-1.983947	1.780414	-1.240403
C	-1.535908	3.070923	-0.954183
C	-0.917173	3.331241	0.270509
C	-0.731407	2.295602	1.185940

Br	-2.509301	-0.963655	-0.742509
H	4.473515	-0.519341	-1.994527
H	4.983081	1.712082	-0.985343
H	3.595715	2.600815	0.875136
H	1.672007	1.327865	1.751392
H	0.664270	-3.319115	-0.405816
H	2.661779	-2.655959	-1.778344
H	-1.873980	-0.417410	2.404676
H	-0.308921	0.314439	2.774105
H	-2.486655	1.563957	-2.183965
H	-1.681988	3.868607	-1.685701
H	-0.575754	4.339431	0.513987
H	-0.250093	2.508197	2.143157

8_iodoethanone_1

Datum	Value
B3LYP-D3(BJ)/Def2SVP Energy	-1044.263172
B3LYP-D3(BJ)/Def2SVP Free Energy (Quasiharmonic)	-1044.063749
Number of Imaginary Frequencies	0

Frequencies (Top 3 out of 87)

```
1      16.6893 cm-1
2      28.2695 cm-1
3      38.5437 cm-1
```

B3LYP-D3(BJ)/Def2SVP Molecular Geometry in Cartesian Coordinates

C	-4.593871	-1.430196	-1.201551
C	-5.653784	-0.601809	-0.836625
C	-5.462078	0.487199	0.041021
C	-4.207741	0.775077	0.575437
C	-3.120846	-0.045099	0.223881
C	-3.336224	-1.136615	-0.662444
C	-1.709634	-0.058181	0.563891
C	-1.152664	-1.137534	-0.119582
N	-2.115155	-1.769603	-0.840876
C	-1.019771	0.884610	1.433883
C	0.497626	0.750292	1.581816
O	-1.611755	1.793056	2.009991
C	1.225610	1.317406	0.379942
C	2.230791	0.645577	-0.336264
C	2.863021	1.227341	-1.442460
C	2.495672	2.508361	-1.856154
C	1.502895	3.203272	-1.159484
C	0.883978	2.610394	-0.061242

I	2.894003	-1.312288	0.219616
H	-4.737334	-2.272990	-1.880684
H	-6.650645	-0.799546	-1.237513
H	-6.316793	1.114860	0.304560
H	-4.053168	1.613472	1.254208
H	-0.125825	-1.496415	-0.137124
H	-1.958912	-2.585945	-1.421178
H	0.777235	1.316678	2.482697
H	0.777864	-0.297730	1.744452
H	3.640440	0.682226	-1.979359
H	2.989298	2.958530	-2.720240
H	1.210107	4.208014	-1.471688
H	0.107821	3.152312	0.484019

8_iodoethanone_2

Datum	Value
B3LYP-D3(BJ)/Def2SVP Energy	-1044.260896
B3LYP-D3(BJ)/Def2SVP Free Energy (Quasiharmonic)	-1044.061429
Number of Imaginary Frequencies	0

Frequencies (Top 3 out of 87)

1 21.1835 cm-1
2 27.9634 cm-1
3 44.1563 cm-1

B3LYP-D3(BJ)/Def2SVP Molecular Geometry in Cartesian Coordinates

C	-4.025848	1.966081	0.681712
C	-3.024605	2.891282	0.965880
C	-1.675010	2.607610	0.670818
C	-1.295153	1.399471	0.090984
C	-2.280911	0.438399	-0.207627
C	-3.638106	0.754703	0.098721
C	-2.296853	-0.893912	-0.793652
C	-3.629508	-1.297330	-0.809417
N	-4.415907	-0.326236	-0.283557
C	-1.229054	-1.761854	-1.295400
C	0.212433	-1.286403	-1.176087
O	-1.472888	-2.856327	-1.793788
C	0.736300	-1.267448	0.245234
C	1.817100	-0.463758	0.650968
C	2.292220	-0.469820	1.966339
C	1.687723	-1.297250	2.915016
C	0.621424	-2.116850	2.538964
C	0.160326	-2.098169	1.222661

I	2.816876	0.819456	-0.735888
H	-5.074673	2.173504	0.903559
H	-3.288296	3.848197	1.421867
H	-0.908844	3.351156	0.902376
H	-0.243372	1.218606	-0.119452
H	-4.043268	-2.235798	-1.170172
H	-5.423278	-0.382388	-0.185461
H	0.826813	-1.964723	-1.788906
H	0.300185	-0.284310	-1.621862
H	3.129533	0.168684	2.251070
H	2.056120	-1.298537	3.943215
H	0.145389	-2.773103	3.270583
H	-0.671148	-2.746705	0.939776

8_iodoethanone_3

Datum	Value
B3LYP-D3(BJ)/Def2SVP Energy	-1044.261577
B3LYP-D3(BJ)/Def2SVP Free Energy (Quasiharmonic)	-1044.062575
Number of Imaginary Frequencies	0

Frequencies (Top 3 out of 87)

1	15.0700 cm-1
2	23.3469 cm-1
3	53.7203 cm-1

B3LYP-D3(BJ)/Def2SVP Molecular Geometry in Cartesian Coordinates

C	-5.290199	-0.216438	0.193649
C	-5.250138	-0.047925	1.575380
C	-4.054105	0.324570	2.223010
C	-2.875436	0.534640	1.510435
C	-2.877677	0.369048	0.110471
C	-4.103962	-0.004350	-0.517186
C	-1.896552	0.490877	-0.957295
C	-2.567278	0.195004	-2.140450
N	-3.865419	-0.097525	-1.878355
C	-0.472550	0.833617	-0.945984
C	0.177112	1.093109	0.415484
O	0.179149	0.915733	-1.979152
C	1.663361	1.297325	0.341468
C	2.576664	0.237521	0.209002
C	3.954880	0.455124	0.122754
C	4.449723	1.761336	0.163010
C	3.565911	2.835585	0.289088
C	2.193584	2.597621	0.376620

I	1.893371	-1.786103	0.129234
H	-6.210335	-0.502503	-0.319857
H	-6.156628	-0.204887	2.164206
H	-4.051917	0.452300	3.307968
H	-1.971945	0.825611	2.045106
H	-2.169863	0.177471	-3.152372
H	-4.556783	-0.350943	-2.575182
H	-0.306903	1.985291	0.845016
H	-0.073831	0.255180	1.083583
H	4.640902	-0.387369	0.024353
H	5.526607	1.931919	0.096331
H	3.944233	3.859595	0.321147
H	1.502513	3.438626	0.475262

8_iodoethanone_4

Datum	Value
B3LYP-D3(BJ)/Def2SVP Energy	-1044.262621
B3LYP-D3(BJ)/Def2SVP Free Energy (Quasiharmonic)	-1044.063081
Number of Imaginary Frequencies	0

Frequencies (Top 3 out of 87)

1	19.6659 cm-1
2	28.2419 cm-1
3	45.6145 cm-1

B3LYP-D3(BJ)/Def2SVP Molecular Geometry in Cartesian Coordinates

C	-4.302291	1.038434	-1.293590
C	-5.139495	-0.011654	-0.921071
C	-4.736506	-0.957577	0.045937
C	-3.489949	-0.877617	0.663896
C	-2.624001	0.171895	0.306373
C	-3.051498	1.113143	-0.670236
C	-1.284540	0.567154	0.712486
C	-0.979429	1.710728	-0.025945
N	-2.024543	2.028731	-0.834710
C	-0.476322	-0.133844	1.706661
C	0.951122	0.328836	2.046795
O	-0.932518	-1.081538	2.337778
C	1.800826	0.946009	0.966741
C	2.187693	0.263161	-0.199974
C	2.955687	0.876087	-1.194787
C	3.364994	2.202085	-1.033980
C	3.012724	2.901168	0.122975
C	2.244605	2.273615	1.104295

I	1.616948	-1.768241	-0.516825
H	-4.607586	1.773502	-2.041062
H	-6.124094	-0.103258	-1.385566
H	-5.418884	-1.767774	0.314318
H	-3.175469	-1.602882	1.413140
H	-0.084384	2.323925	-0.025045
H	-2.041070	2.819288	-1.468767
H	0.846362	1.051131	2.873241
H	1.443120	-0.559354	2.471687
H	3.235405	0.322390	-2.092120
H	3.962570	2.680337	-1.813156
H	3.333893	3.935562	0.261989
H	1.962103	2.825368	2.004342

8_iodoethanone_5

Datum	Value
B3LYP-D3(BJ)/Def2SVP Energy	-1044.263697
B3LYP-D3(BJ)/Def2SVP Free Energy (Quasiharmonic)	-1044.064986
Number of Imaginary Frequencies	0

Frequencies (Top 3 out of 87)

```
1      11.7857 cm-1
2      18.0651 cm-1
3      43.7459 cm-1
```

B3LYP-D3(BJ)/Def2SVP Molecular Geometry in Cartesian Coordinates

C	-5.290048	-0.319026	0.230443
C	-5.602115	0.103688	-1.060343
C	-4.629620	0.706588	-1.887398
C	-3.322701	0.901566	-1.445394
C	-2.980753	0.484649	-0.146348
C	-3.976069	-0.120609	0.670140
C	-1.760926	0.516702	0.639580
C	-2.073551	-0.059126	1.868802
N	-3.380873	-0.433043	1.882541
C	-0.474328	1.054914	0.214017
C	0.668980	1.057100	1.239698
O	-0.302965	1.523243	-0.904966
C	2.026527	1.192974	0.608715
C	2.665481	0.132905	-0.057314
C	3.919994	0.282264	-0.654969
C	4.565083	1.520811	-0.599677
C	3.952326	2.594929	0.049563
C	2.700260	2.424694	0.642563

I	1.725869	-1.781888	-0.197517
H	-6.038614	-0.786059	0.873510
H	-6.617768	-0.034570	-1.438342
H	-4.910866	1.024881	-2.894148
H	-2.567057	1.364955	-2.079174
H	-1.444441	-0.228954	2.739475
H	-3.845860	-0.879216	2.665010
H	0.485126	1.910363	1.913188
H	0.618117	0.152108	1.861661
H	4.393128	-0.559757	-1.162183
H	5.545696	1.638858	-1.066250
H	4.448825	3.566591	0.095593
H	2.221247	3.266055	1.149542

8_iodoethanone_6

Datum	Value
B3LYP-D3(BJ)/Def2SVP Energy	-1044.256773
B3LYP-D3(BJ)/Def2SVP Free Energy (Quasiharmonic)	-1044.057468
Number of Imaginary Frequencies	0

Frequencies (Top 3 out of 87)

1	17.3772 cm-1
2	29.9341 cm-1
3	52.5407 cm-1

B3LYP-D3(BJ)/Def2SVP Molecular Geometry in Cartesian Coordinates

C	-4.012915	-0.642355	-1.350747
C	-4.513547	0.562648	-0.864112
C	-3.889896	1.223009	0.215811
C	-2.745048	0.706429	0.816570
C	-2.190037	-0.490783	0.326847
C	-2.862325	-1.156862	-0.741402
C	-1.068675	-1.336543	0.695132
C	-1.133744	-2.452150	-0.132100
N	-2.181904	-2.336343	-0.989684
C	0.018353	-1.185029	1.672498
C	0.489333	0.215752	2.084215
O	0.576634	-2.163508	2.150205
C	0.602967	1.270877	1.007080
C	1.354957	1.121041	-0.172809
C	1.414071	2.126366	-1.144536
C	0.733131	3.328601	-0.943702
C	0.011962	3.524251	0.235568
C	-0.044019	2.507054	1.187260

I	2.531347	-0.623063	-0.541852
H	-4.501273	-1.172586	-2.171002
H	-5.409423	0.994985	-1.315518
H	-4.317367	2.155651	0.591230
H	-2.298900	1.223099	1.663809
H	-0.456064	-3.301474	-0.171673
H	-2.418649	-3.006618	-1.711996
H	-0.207267	0.577381	2.859059
H	1.457686	0.059641	2.584297
H	1.998959	1.975421	-2.052865
H	0.780974	4.110259	-1.705098
H	-0.510109	4.466305	0.415467
H	-0.607141	2.668895	2.109147

8_iodoethanone_7

Datum	Value
B3LYP-D3(BJ)/Def2SVP Energy	-1044.256773
B3LYP-D3(BJ)/Def2SVP Free Energy (Quasiharmonic)	-1044.057467
Number of Imaginary Frequencies	0

Frequencies (Top 3 out of 87)

1	17.3918 cm-1
2	29.9799 cm-1
3	52.5576 cm-1

B3LYP-D3(BJ)/Def2SVP Molecular Geometry in Cartesian Coordinates

C	4.012962	-0.642282	1.350709
C	4.513655	0.562599	0.863817
C	3.890017	1.222780	-0.216229
C	2.745102	0.706177	-0.816838
C	2.189977	-0.490866	-0.326805
C	2.862301	-1.156808	0.741528
C	1.068500	-1.336564	-0.694819
C	1.133512	-2.452017	0.132649
N	2.181853	-2.336219	0.990008
C	-0.018501	-1.185202	-1.672251
C	-0.489305	0.215511	-2.084270
O	-0.576810	-2.163768	-2.149790
C	-0.602943	1.270786	-1.007250
C	-1.354958	1.121082	0.172652
C	-1.414030	2.126548	1.144256
C	-0.733023	3.328715	0.943304
C	-0.011833	3.524203	-0.235981
C	0.044083	2.506910	-1.187584
I	-2.531324	-0.622953	0.541948

H	4.501332	-1.172377	2.171049
H	5.409590	0.994966	1.315102
H	4.317581	2.155288	-0.591900
H	2.299022	1.222643	-1.664246
H	0.455806	-3.301310	0.172429
H	2.417956	-3.005935	1.713056
H	0.207348	0.576913	-2.859179
H	-1.457678	0.059426	-2.584349
H	-1.998959	1.975726	2.052594
H	-0.780824	4.110489	1.704596
H	0.510268	4.466239	-0.415954
H	0.607155	2.668666	-2.109514
